# Dynamic covalent chemistry with azines†

**DOI:** 10.1039/d2cc03523e

**Published:** 2022-09-08

**Authors:** Anca-Elena Dascalu, Lau Halgreen, Aaron Torres-Huerta, Hennie Valkenier

**Affiliations:** Université libre de Bruxelles (ULB), Ecole polytechnique de Bruxelles, Engineering Molecular NanoSystems, Avenue Franklin Roosevelt 50 1050 Brussels Belgium hennie.valkenier@ulb.be

## Abstract

Dynamic covalent chemistry is used in many applications that require both the stability of covalent bonds and the possibility to exchange building blocks. Here we present azines as a dynamic covalent functional group that combines the best characteristics of imines and acylhydrazones. We show that azines are stable in the presence of water and that dynamic combinatorial libraries of azines and aldehydes equilibrate in less than an hour.

## Abstract 2

Azines provide an attractive balance in dynamic covalent chemistry, with rapid exchange in acidic conditions and good stability in the presence of water.

## Graphical abstract

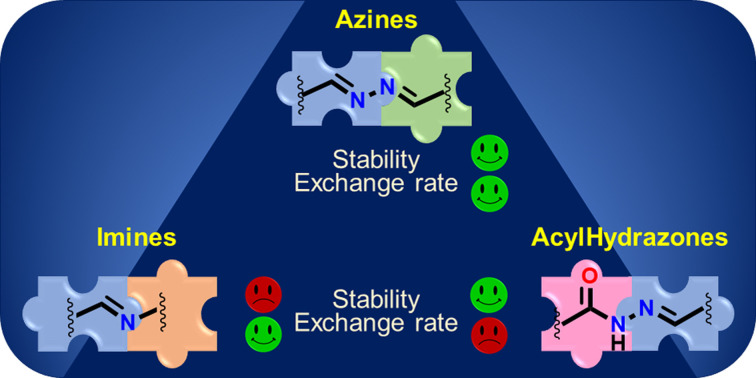

Dynamic covalent chemistry (DCC) is a powerful tool^1–3^ that is widely used to develop functional molecular systems and dynamic materials.^4–7^ Central to DCC is the reversible nature of the linkage, which allows the exchange of the building blocks. The formation of many molecular systems and materials requires a balance between stability and exchange of the dynamic bond. Inadequacies of either of these features have limited the applications of many DCC groups. For example, imines are commonly used to form molecular structures,^8,9^ however, their applications in biomaterials are limited due to their low stability in the presence of water.^10^ In contrast, hydrazones^11^ and acylhydrazones, which are structurally similar to imines, are much slower to hydrolyse, even in pure water.^3,12^ However, hydrazones are generally too stable for use in DCC and the exchange reactions of acylhydrazones are often very slow and usually require an excess of strong acids or nucleophilic catalysts.^13–17^

Functional requirements drive the development of new dynamic functional groups, such as ortho-esters^18^ and tetrazines.^19^ The drawbacks listed above motivated us to explore alternative motifs for DCC that would provide rapid exchange, activated by mild stimuli, in combination with high stability in the presence of water. Azines are readily synthesized through the condensation of hydrazine with aldehydes or ketones.^20^ The high electron density on the C=N–N=C unit provides azines with interesting optoelectronic properties and makes them suitable ligands to coordinate metal ions,^21^ which is attractive for sensors and catalysts. Nevertheless, the dynamic character of azines in solution has not been explored,^22–27^ even though their formation has been known for over a century.^28^ Here, we present the ability of azines to exchange and reach equilibrium rapidly in dynamic combinatorial libraries under various conditions. Combined with their stability, this shows azines to be a promising motif for DCC (Fig. 1).

**Fig. 1:**
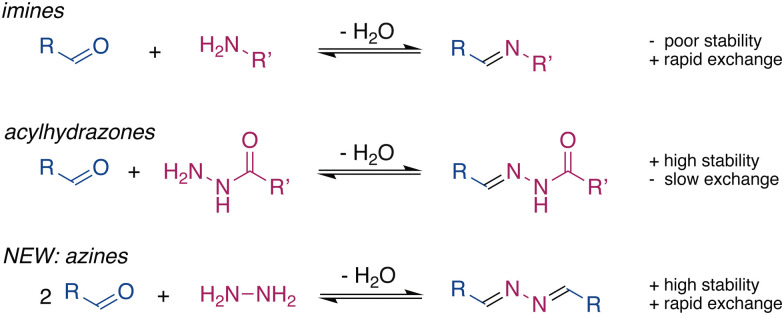
Comparison between imines, *N*-acylhydrazones, and azines.

A series of azines was synthesized from 2 equivalents of benzaldehyde (A) or substituted analogues (B–G, Fig. 2) and 1 equivalent of hydrazine monohydrate in ethanol in 82–98% isolated yield. The obtained azines AA–GG were readily soluble in a wide range of organic solvents (see below).

**Fig. 2:**
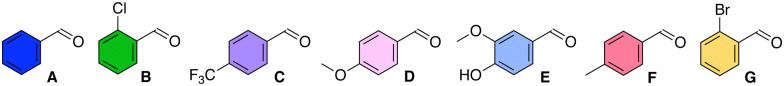
Structures of the aldehydes A–G that were used to prepare azines AA–GG.

The stability of the benzaldehyde-based azine AA was analysed under neutral and acidic conditions in DMSO-d_6_ with D_2_O as co-solvent by ^1^H NMR spectroscopy. No hydrolysis was observed in absence of acid, even in 40% D_2_O in DMSO-d_6_ (Fig S14, ESI†). In acidic conditions (1 eq. trifluoroacetic acid, TFA), an equilibrium was established within one hour, showing 7% or 16% aldehyde A in presence of 5% or 20% D_2_O respectively (Fig. S15–20, ESI†). These percentages did not change over the next 3 days and similar aldehyde/azine equilibria were obtained when starting from benzaldehyde and hydrazine (Table S1, ESI†). Azine CC showed similar results, while the electron donating methoxy group on DD gave rise to a bit more hydrolysis (Table S2, ESI†). Encouragingly, >90% of the azine persisted in all these conditions. Experiments with EE in a mixture of 80% buffered D_2_O and 20% DMSO-d_6_ at pD 5–8 showed that hydrolysis is the lowest at pD 8, while at pD 6 and 7 ∼65% of original azine remains after six days, and 50% at pD 5 (Table S3, ESI†). These various azines demonstrate still much higher aqueous stability compared to most imines. For instance, the imine of benzaldehyde and aniline is fully hydrolysed after one day in presence of 20% buffer (at pD 7.6) in DMSO-d_6_ (Fig. S27, ESI†).

To prove that azines are a dynamic functional group in solution, we have tested the exchange of azine AA with 2-chlorobenzaldehyde B in presence of 1 eq. TFA in chloroform at room temperature. After 1 hour, the ^1^H NMR spectrum showed a library of compounds, consisting of aldehydes A and B, and azines AA, BB, and AB, of which the aldehyde or azomethine protons all showed distinct ^1^H NMR signals (Fig. 3). An identical library was obtained from azine BB and aldehyde A under the same conditions and no changes in the distribution of species were observed during the following days (Fig. S28 and S29, ESI†). These experiments show, firstly, that azines can exchange with aldehydes, a reaction that we refer to as transazination. Secondly, the identical distribution of species in both libraries shows that a thermodynamic equilibrium was obtained, despite the short equilibration time of 1 hour. This implies that the exchange of azines happens much faster than the exchange of acylhydrazones, which requires at least several days to reach equilibrium under such conditions (Fig. S30, ESI†).

**Fig. 3:**
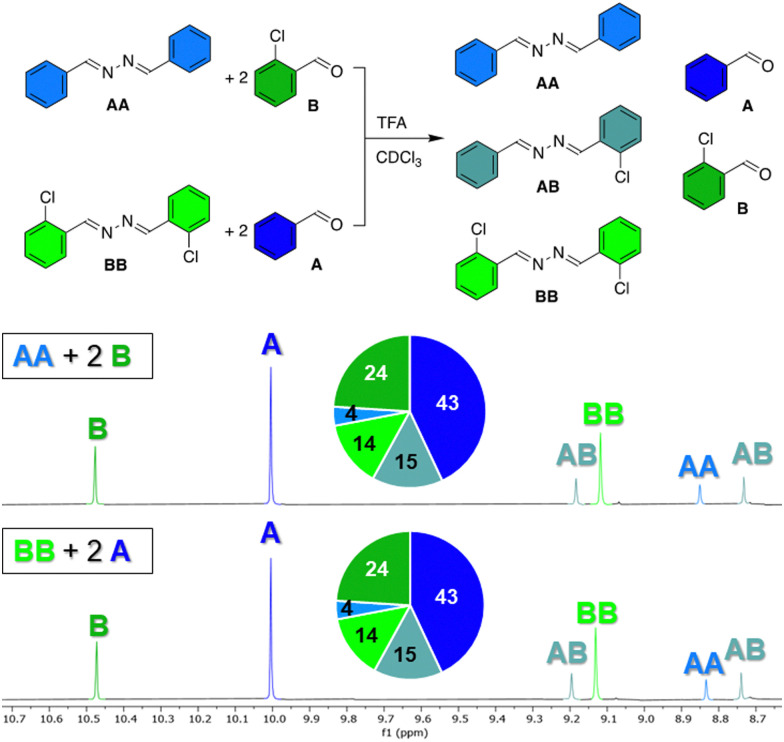
Formation of identical libraries from azine AA + aldehyde B and from azine BB + aldehyde A, as shown by the ^1^H NMR spectra recorded after 1 hour. The pie chart indicates the distribution of the library in mol%. Reaction conditions: azine (7 mM), aldehyde (14 mM), TFA (7 mM) in CDCl_3_.

Encouraged by these results, we continued our investigations regarding conditions that favour the transazination reaction, starting from identical concentrations of azine AA and aldehyde B (14 mM each) and exploring different acids and solvents (Table 1). The use of strong Brønsted acids, such as TFA and *p*-toluenesulfonic acid (PTSA) in chloroform, leads to the equilibration of azines within one hour, giving identical distributions of species (Table 1, entries 1–2). However, when weaker Brønsted acids, such as acetic acid and formic acid, were used, no exchange was visible after a day. After 10 days, traces of exchange products were observed with acetic acid while with formic acid, which has a slightly lower p*K*_a_ compared to acetic acid, the library had reached a distribution nearly identical to that obtained with the stronger acids TFA and PTSA (entries 3–4).

**Table 1:** Scope of azine exchange conditions

Entry	Additive (eq.)	Solvent	Time	A/B/AA/BB/AB	EQ
Effect of acids					
1	TFA (1)	CDCl_3_	1 h	42/10/16/9/23	√
			24 h	43/8/16/10/23	√
2	PTSA (1)	CDCl_3_	1 h	43/8/16/9/23	√
			24 h	44/8/16/9/23	√
3	Acetic acid (1)	CDCl_3_	24 h	0/50/50/0/0	X
			10 d	6/46/46/2/0	X
4	Formic acid (1)	CDCl_3_	24 h	0/50/50/0/0	X
			10 d	36/17/18/6/24	√
5	TFA (1)	CD_3_CN	1 h	43/9/15/10/24	√
			24 h	43/8/15/9/24	√
6	TFA (1)	Toluene-d_8_	1 h	46/10/13/10/21	√
			24 h	46/8/15/9/22	√
Effect of water content					
7	TFA (1)	Dry DMSO-d_6_	1 h	0/50/50/0/0	X
			7 d	9/44/38/1/8	X
8	TFA (1)	DMSO-d_6_ + 1% D_2_O	1 h	3/49/45/0/4	X
			7 d	44/9/13/10/24	√
9	TFA (1)	DMSO-d_6_ + 5% D_2_O	1 h	15/26/43/1/14	X
			24 h	38/5/24/6/27	√
10	TFA (1)	DMSO-d_6_ + 20% D_2_O	1 h	42/7/16/9/25	√
			24 h	43/6/19/7/24	√
11	TFA (0.1)	DMSO-d_6_ + 20% D_2_O	1 h	8/39/45/0/7	X
			24 h	42/7/18/8/25	√
12	TFA (0.1)	CD_3_CN	1 h	9/34/47/1/9	X
			24 h	38/6/25/6/25	√
13	TFA (0.1)	CD_3_CN + 5% D_2_O	1 h	30/14/30/4/22	∼
			24 h	40/5/22/7/26	√
14	TFA (0.1)	CD_3_CN + 10% D_2_O	1 h	32/10/29/5/24	∼
			24 h	39/5/25/6/25	√
15	None	CD_3_CN + 10% D_2_O	1 h	2/44/1/53/0	X
			5 d	37/7/25/6/25	√
16	None	CD_3_CN	4 d	0/45/54/0/0	X
17	None	DMSO-d_6_ + 5% D_2_O	10 d	0/49/51/0/0	X

When testing the transazination in different solvents with 1 eq. TFA as catalyst, rapid equilibration (within 1 hour) was observed in acetonitrile and toluene, similar to the results obtained in chloroform (Table 1, entries 5–6). In DMSO, the rate of azine exchange was found to be strongly dependent on the water content (entries 7–10). In dried DMSO with 1 eq. of TFA, only traces of exchange products were visible after one week, while the addition of 1% of water allowed the library to fully equilibrate. The addition of 20% of water as co-solvent in DMSO led to full equilibration within an hour, restoring the fast exchange observed in other solvents.

To open further opportunities for the applications of azines in DCC, we have explored the possibility to use milder and truly catalytic exchange conditions (entries 11–14). With 0.1 eq. of TFA in 20% D_2_O in DMSO-d_6_, the exchange took more time than when 1 eq. was used, but equilibration was still achieved within 24 hours. In acetonitrile with 5–10% D_2_O and 0.1 eq. TFA, equilibration was faster and nearly completed in 1 h. Based on these results, we have repeated the experiments in 10% D_2_O in acetonitrile in absence of acid and found that equilibration is slow but possible, with the equilibrium reached in 5 days (entry 15). When much lower concentrations of azines and aldehydes were used (0.4 mM), libraries were still found to equilibrate within 24 h (Table S4, ESI†).

The strong impact of the water content on the transazination reaction in both DMSO and acetonitrile and the absence of exchange products in dried DMSO indicate that water plays an important role in the exchange mechanism (see Fig. S51, ESI†). The wide range of solvents in which azines can exchange opens the way to various applications. Furthermore, water could be used to tune the rate of exchange of azines in DMSO or acetonitrile from very fast (<1 h) to rather slow (>1 week), allowing control over the dynamic covalent chemistry.

Having shown that azines are dynamic in the presence of water and/or strong acids, we have explored the freezing of libraries upon the addition of a base (Fig. 4). Azine AA and aldehyde B (14 mM) were combined with 1 eq. TFA in DMSO-d_6_/D_2_O (90/10) and K_2_CO_3_ (2 eq.) was added after different times. All samples were analysed after 5 hours and compared to a sample without base added. Azines AB and BB are not visible in the ^1^H NMR spectrum of the sample that was basified after 3 minutes and only small amounts are visible in the samples to which K_2_CO_3_ was added after 20 or 40 min, while the library without K_2_CO_3_ has equilibrated as expected. Similarly, the addition of K_2_CO_3_ prevented any transazination in acetonitrile/D_2_O (90/10) (Fig. S53 and S54, ESI†). These experiments show that, analogously to acylhydrazones and disulfides, it is possible to use the pH to switch transazination on or off. Furthermore, it opens up the possibility of utilising azines in combination with base catalysed dynamic bonds, such as disulfides, thioesters, or boronic esters, in orthogonal dynamic covalent systems.

**Fig. 4:**
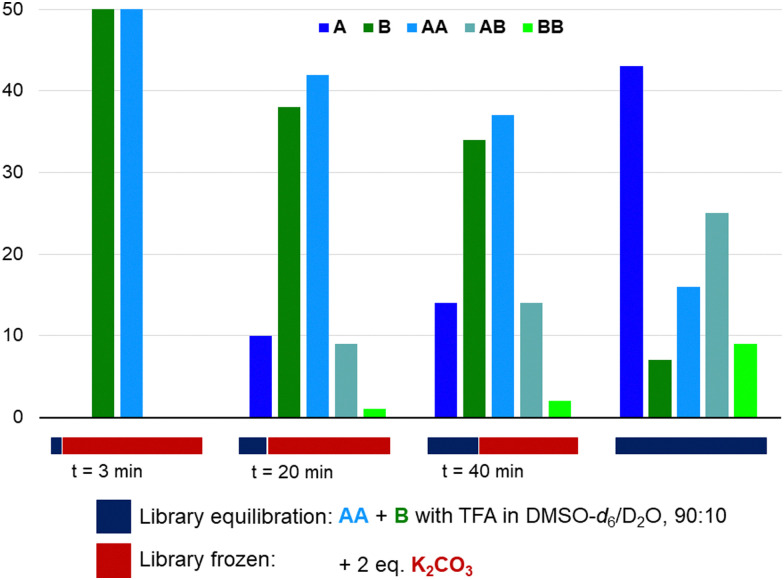
The equilibration of a library starting from AA (14 mM) and B (14 mM) with TFA (14 mM) in DMSO-d_6_/D_2_O (90 : 10) can be brought to a halt by the addition of K_2_CO_3_ (28 mM). The libraries were analysed by ^1^H NMR spectroscopy after 5 hours, independent of the moment at which the library was frozen, and the results are shown in mol%.

To test the impact of electron withdrawing and donating substituents, azine AA (7 mM) was combined with 2 eq. of aldehyde B, C, or D with 1 eq. TFA in acetonitrile (Fig. S55–S58, ESI†). These experiments revealed that azines BB and CC are preferred compared to AA, while azine DD is formed in lower quantities. Electron withdrawing groups thus have a stabilising effect on the azines. All libraries had reached equilibrium after 24 h.

After having extensively studied the exchange of azines and aldehydes, we have explored azine metathesis (Fig. 5). For this, different azines (AA + BB or FF + GG, 2 mM each) are combined in DMSO-d_6_/D_2_O (95/5) in presence of 1 eq. TFA. A statistical mixture with ∼50% of asymmetric azine was obtained after one hour in both cases. Even though only small traces of aldehydes are visible in the ^1^H NMR spectra of these experiments, these are still likely to play a key role in the exchange mechanism of azines. This is confirmed by an experiment in absence of water, which showed no significant azine metathesis (Fig. S59, ESI†).

**Fig. 5:**
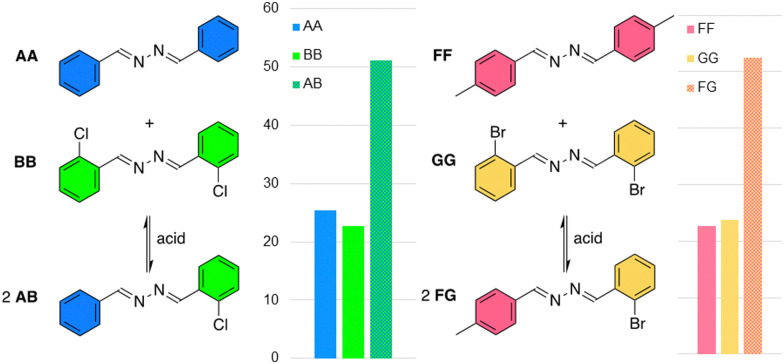
Azine metathesis, showing the exchange of azines AA + BB to form AB (left) or FF + GG to form FG (right), as determined from the ^1^H NMR spectra recorded after 1 hour and shown in mol%. Reaction conditions: each azine derivative (2 mM), TFA (2 mM) in D_2_O/DMSO-d_6_ (5 : 95) at room temperature. Only traces of hydrolysis were observed (<3%).

Here we have demonstrated that azines can be used for dynamic covalent chemistry. They tolerate the presence of significant amounts of water with only limited hydrolysis, which is a major advantage compared to imines. Furthermore, azines allow rapid exchange and equilibration of libraries in presence of strong acids in a wide variety of solvents. Their fast exchange kinetics and good solubility is a great advantage compared to acylhydrazones. The exchange rate can be tuned by the amounts of water and acid catalyst in the system and exchange can be turned off completely upon the addition of a base. This pH-dependent on/off switching of exchange enables the use of azines in orthogonal dynamic covalent chemistry. Additionally, as azines are formed from aldehydes combined with hydrazine, materials or compounds will only require the functionalisation with a single type of functional group to access azine-based dynamic covalent chemistry.

The great stability of azines and versatility of their exchange conditions open the way to many applications ranging from bioconjugation to materials chemistry. While writing this manuscript, an example of polyurethane networks containing azines was reported, showing that azines can contribute to the re-processibility of polymers upon heating.^27^ This is only one example of an application of azines in dynamic covalent chemistry and we are convinced that many more will follow.

The results reported here are part of a project that has received funding from the European Research Council (ERC) under the European Union's Horizon 2020 research and innovation programme (Grant agreement No. 802727). HV is a research associate of the Fonds de la Recherche Scientifique – FNRS.

## Conflicts of interest

There are no conflicts to declare.

## Supplementary Material

CC-058-D2CC03523E-s001
