# Supplementary material for: Dynamic covalent chemistry with azines
Source: Chem Commun (Camb). 2022 Sep 8;58(79):11103–6. doi: 10.1039/d2cc03523e (PMC9531658; doi:10.1039/d2cc03523e)
Supplement: CC-058-D2CC03523E-s001 [file CC-058-D2CC03523E-s001.pdf]

Electronic Supplementary Material (ESI) for ChemComm.
This journal is © The Royal Society of Chemistry 2022

Electronic Supplementary Information

Dynamic covalent chemistry with azines

Contents

1. Reagents and solvents........2
2. Instruments and conditions........2
3. Synthesis and characterisation of azine compounds........2
4. Calculation of molar ratios........10
5. Azines stability in acidic conditions and aqueous solutions........10
6. Imine stability in aqueous solution.........25
7. Proof of reversibility........26
8. Acylhydrazone control experiments........29
9. Azine exchange reactions........30
   9.1 Effect of Brønsted acids........31
   9.2 Effect of water content........38
   9.3 Low concentration DCLs with water as co-solvent........49
10. The role of water in the mechanism........51
11. DCLs freezing........52
12. The effect of electron donating and withdrawing substituents on exchange........55
13. Azine metathesis reactions........59
14. Appendix 1........62
15. References........66

1. Reagents and solvents

All commercially available reagents were purchased from Sigma Aldrich, Merck, or Alfa Aesar and used without further purification. Deuterated solvents used for exchange reactions were used as such without being dried. Where mentioned, the drying of solvent was done with molecular sieves 3Å, for at least 2 d.

2. Instruments and conditions

NMR spectra were recorded on Varian VNMRS 400 (^{1}H: 400 MHz, ^{13}C: 100 MHz) equipped with a OneNMR probe (25°C, 2.6 µs pulse, 3 s acquisition time, 3 s relaxation delay). Chemical shifts (δ) are given in parts per million (ppm) and referenced to the corresponding residual solvent peak. Coupling constants (J) are denoted in Hertz (Hz). Abbreviations indicating multiplicity were used as follows: s = *singlet*, d = *doublet*, dd = *double doublet*, m = *multiplet*.

3. Synthesis and characterisation of azine compounds

The syntheses of azines were conducted via one general procedure: the aldehyde and hydrazine monohydrate were mixed in a 2:1 ratio, in ethanol. In a typical experiment, an immediate colour change is observed. The completion of the reaction was monitored by thin-layer chromatography (silica gel; ethyl acetate:*n*-heptane, 4:6). The product was collected by suction filtration, washed with ethanol, and dried under vacuum. All the synthesized azines were yellow solids.

1,2-di((E)-benzylidene)hydrazine (AA)[1]

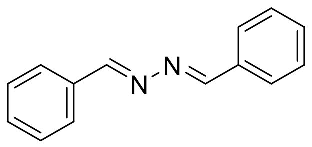

Benzaldehyde (1.91, 18.8 mmol, 2 eq.) was added dropwise to a solution of hydrazine monohydrate (0.48 mL, 9.4 mmol, 1 eq.) in ethanol (25 mL). The mixture was heated at 50 °C for 20 minutes and then allowed to cool down to room temperature. A precipitate was formed immediately after the mixture was cooled down. The precipitate was filtered and washed with ethanol to give a yellow solid in 84% isolated yield (1.65 g, 7.9 mmol). **^{1}H NMR** (400 MHz, $CDCl_3$): δ = 8.67 (s, 2H), 7.87 – 7.84 (m, 4H), 7.47 – 7.45 (m, 6H) ppm. **^{13}C NMR** (100 MHz, $CDCl_3$): δ = 162.2, 134.2, 131.4, 128.9, 128.7 ppm.

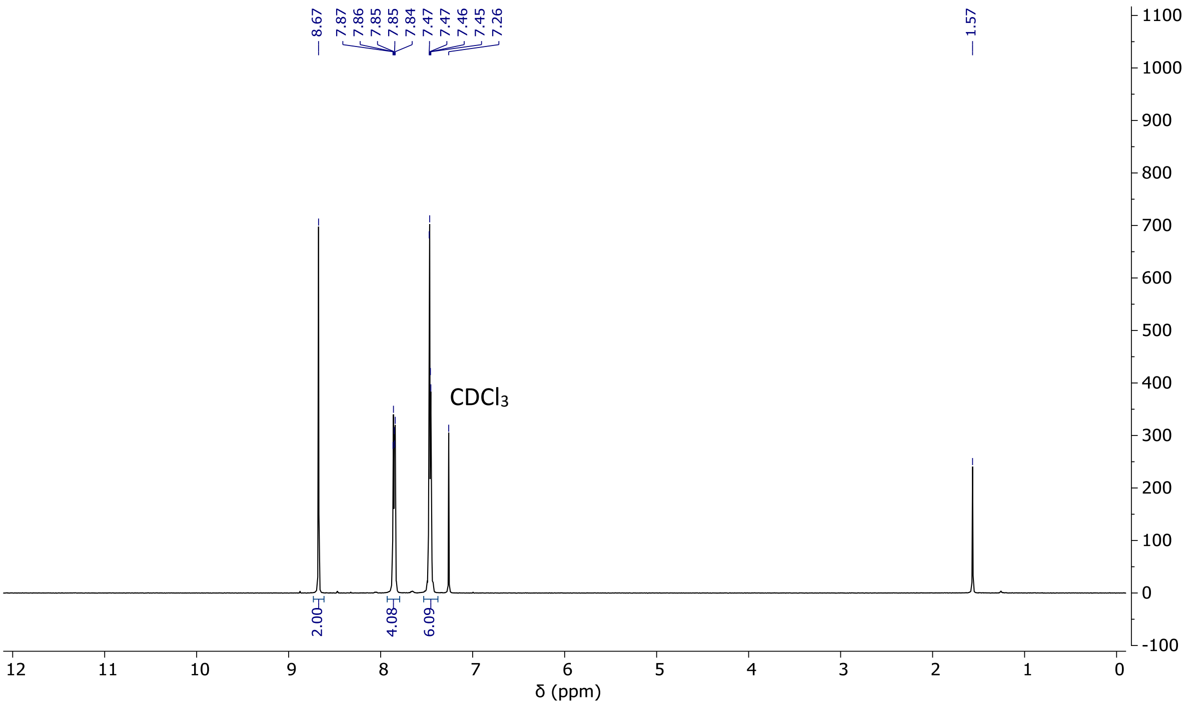

Figure S1: ^{1}H NMR spectrum of compound **AA**.

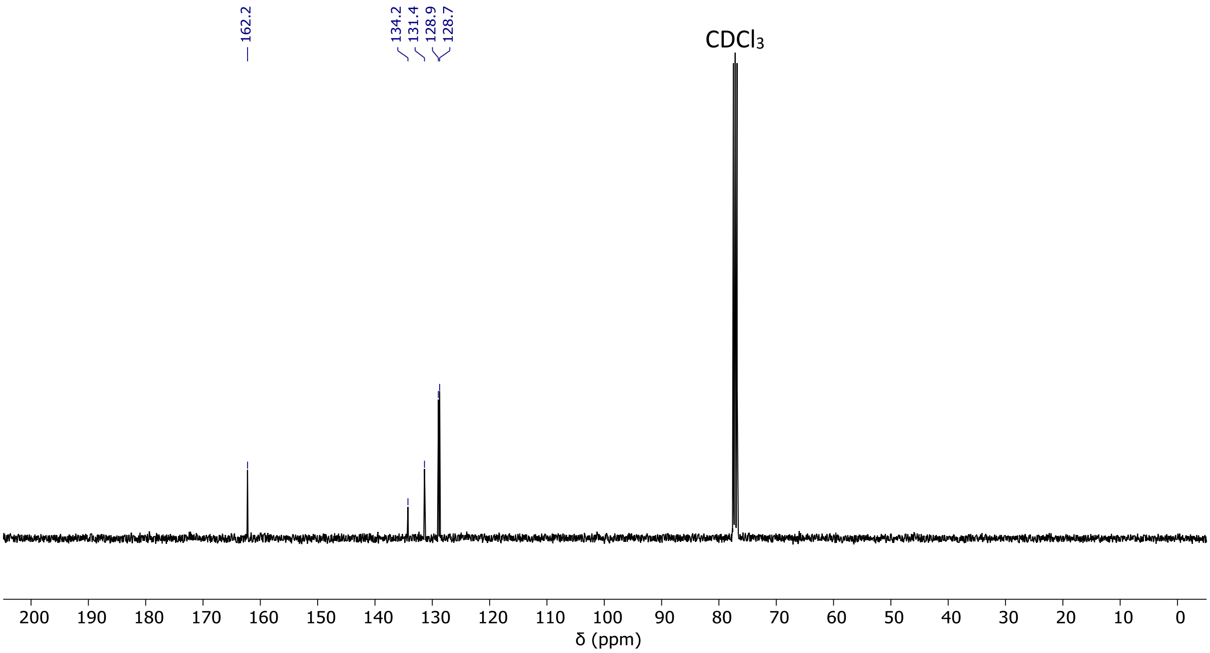

Figure S2: ^{13}C NMR spectrum of compound **AA**.

1,2-bis((E)-2-chlorobenzylidene)hydrazine (BB)[2]

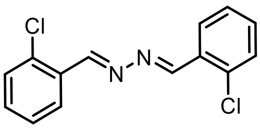

2-Chlorobenzaldehyde (1mL, 8.9 mmol, 2 eq.) was added dropwise over a solution of hydrazine monohydrate (0.22 mL, 4.45 mmol, 1 eq.) in ethanol (25 mL) and stirred for 30 minutes. The mixture was stirred for 2 minutes and precipitated. The precipitate was filtrated and washed with ethanol to give an as yellow solid in 89% isolated yield (1.1 g, 3.96 mmol). **^{1}H NMR** (400 MHz, $CDCl_3$): δ = 9.09 (s, 2H), 8.22 (dd, J = 7.6, 1.9 Hz, 2H), 7.45 – 7.33 (m, 6H) ppm. **^{13}C NMR** (100 MHz, $CDCl_3$): δ = 159.2, 136.0, 132.3, 131.6, 130.2, 128.5, 127.2 ppm.

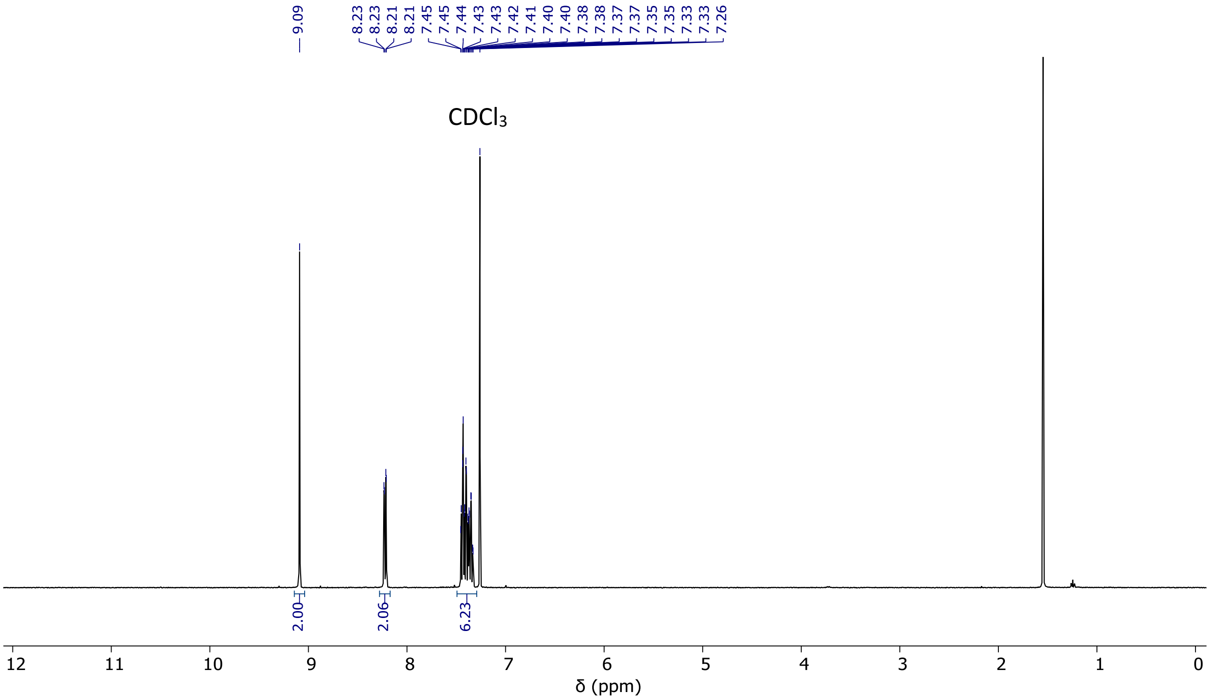

Figure S3: ^{1}H NMR spectrum of compound **BB**.

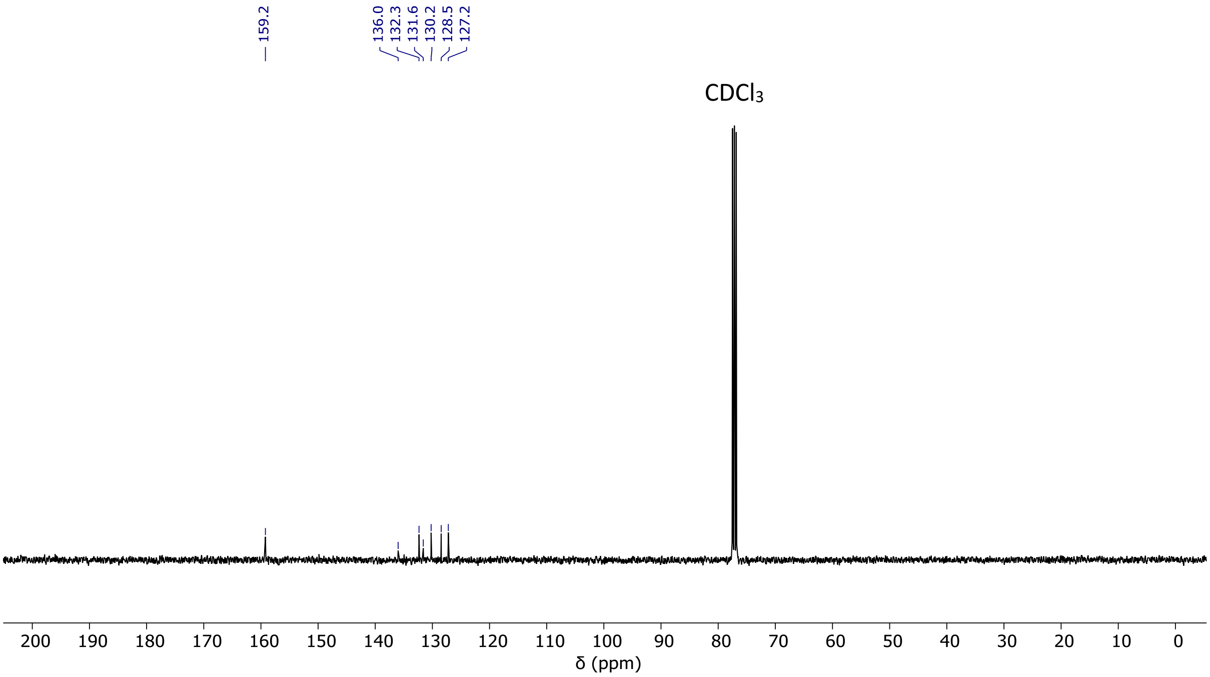

Figure S4: ^{13}C NMR spectrum of compound **BB**.

1,2-bis(4-(trifluoromethyl)benzylidene)hydrazine (CC)[3]

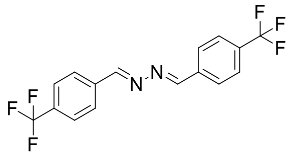

4-(trifluoromethyl)benzaldehyde (0.78 mL, 5.7 mmol, 2 eq.) was added dropwise over a solution of hydrazine monohydrate (0.15 mL, 2.9 mmol, 1 eq.) in ethanol (20 mL) and stirred for one hour, until precipitation of the compound. The precipitate was filtrated and washed with ethanol to give an yellowish solid in 85% isolated yield (0.85 g, 2.4 mmol). **^{1}H NMR** (400 MHz, $CDCl_3$): δ = 8.68 (s, 1H), 7.97 (d, J = 8.0 Hz, 2H), 7.72 (d, J = 8.0 Hz, 2H) ppm.**^{13}C NMR** (100 MHz, $CDCl_3$): δ = 161.1, 137.1, 132.9, 128.8, 125.7, 123.7 ppm.

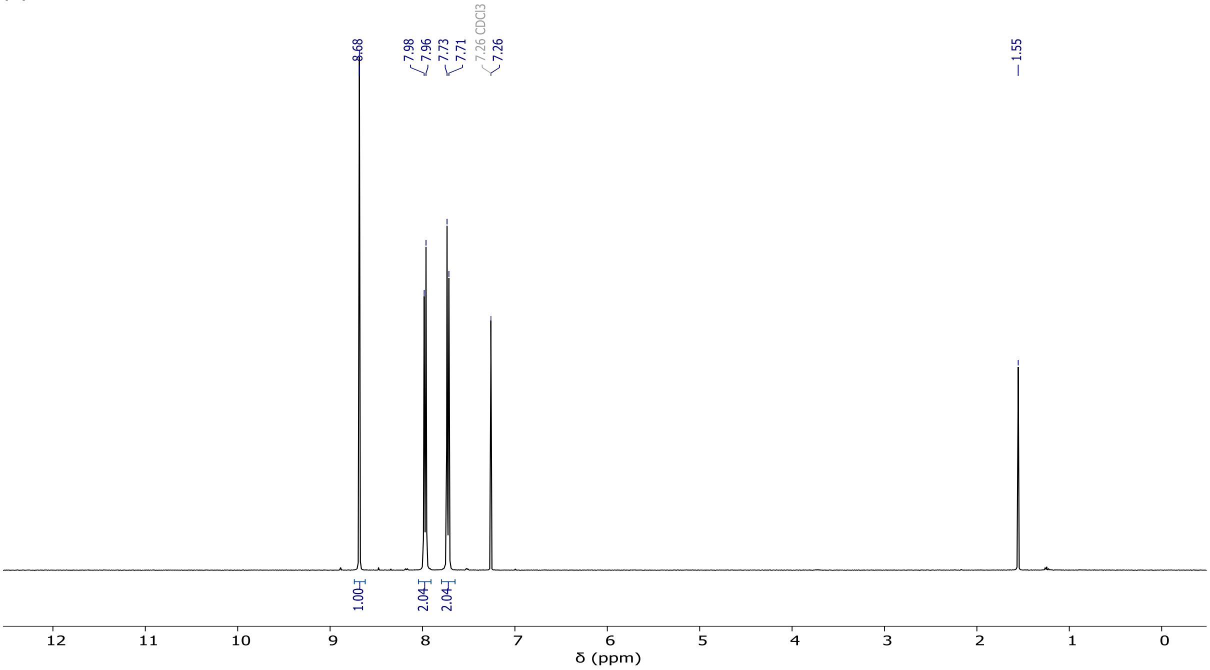

Figure S5: ^{1}H NMR spectrum of compound **CC**

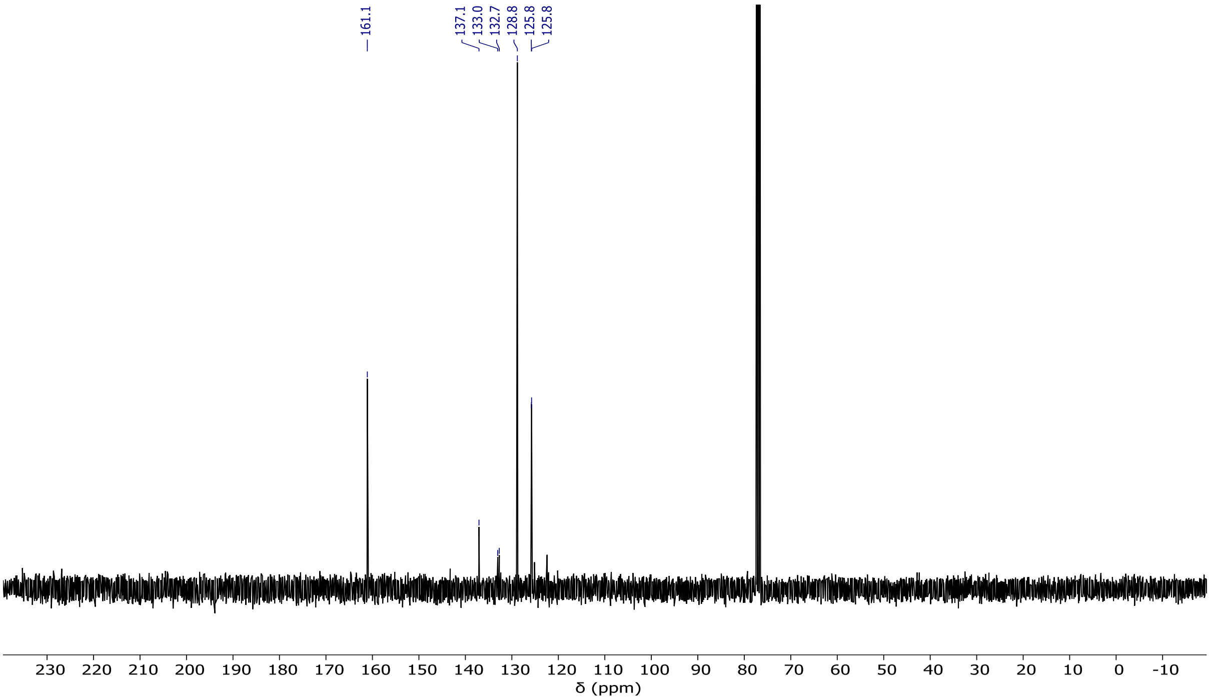

Figure S6: ^{13}C NMR spectrum of the compound **CC**.

1,2-bis(4-methoxybenzylidene)hydrazine (DD)[3]

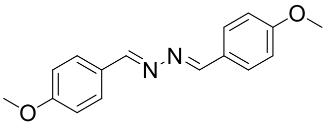

4-Methoxybenzaldehyde (1.26 mL, 11 mmol, 2 eq.) was added dropwise over a solution of hydrazine monohydrate (0.27 mL, 5.5 mmol, 1 eq.) in ethanol (25 mL) and stirred for one hour, until precipitation of the compound. The precipitate was filtrated and washed with ethanol to give an yellowish solid in 98% isolated yield (1.45 g, 5.4 mmol). **^{1}H NMR** (400 MHz, $CDCl_3$): δ = 8.61 (s, 1H), 7.81 – 7.74 (m, 2H), 7.01 – 6.89 (m, 2H), 3.86 (s, 3H) ppm. **^{13}C NMR** (100 MHz, $CDCl_3$): δ = 161.98, 161.06, 130.10, 127.00, 114.23, 55.38 ppm.

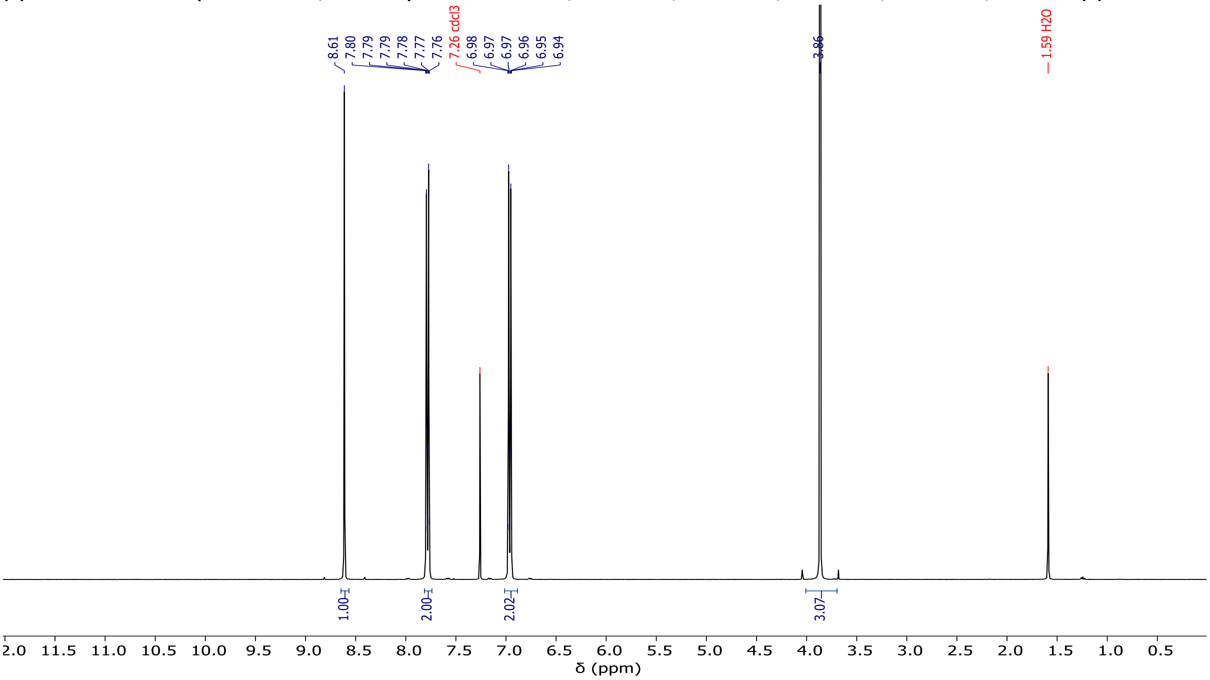

Figure S7: ^{1}H NMR spectrum of compound **DD.**

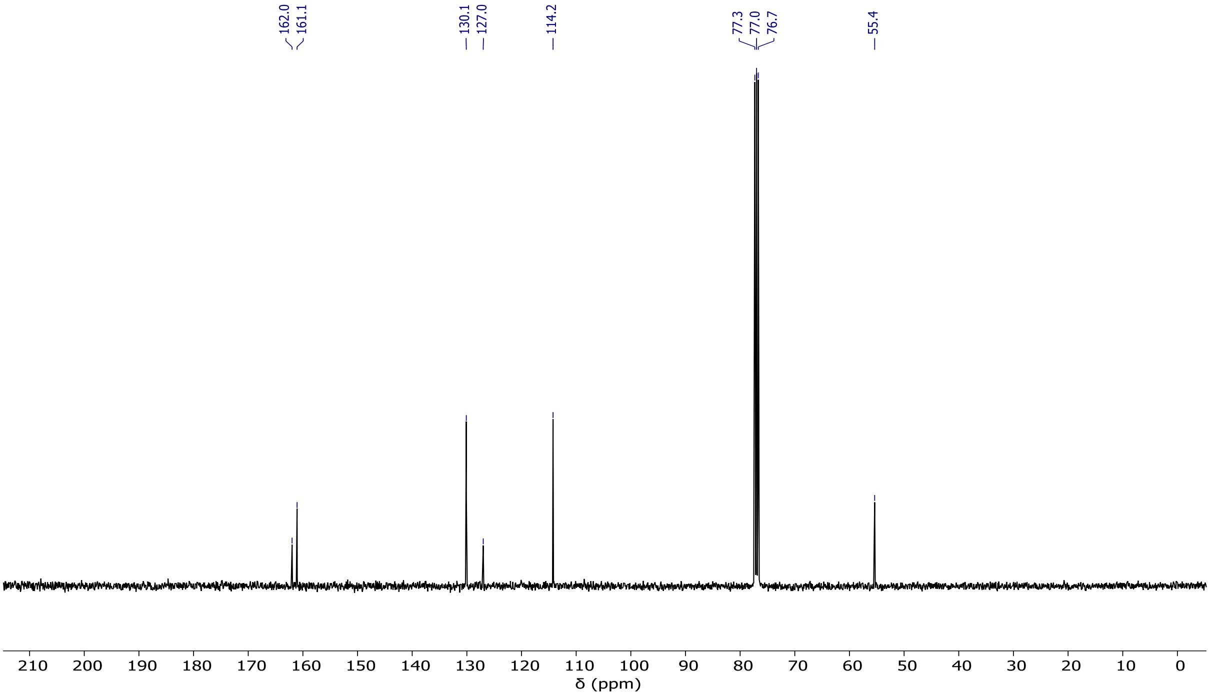

Figure S8: ^{13}C NMR spectrum of the compound **DD**.

4-hydroxy-3-methoxybenzaldehyde azine (EE)[4]

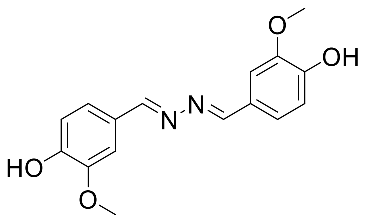

Vanilin (50 g, 328.6 mmol) was dissolved in 100 ml ethanol at 50 degrees and hydrazine monohydrate (8.23 mL, 164.3 mmol, 0.5 eq.) was added dropwise over 15 minutes. The resulting mixture was stirred for 1 hour, cooled to room temperature and put in the fridge overnight. The resulting crystals were filtered on a sintered glass funnel and washed with cold ethanol (2x 30 ml) to yield the desired azine as yellow crystals (97.7 %, 48.2 g, 160.5 mmol). **M.p.**: 179.0-180 (litt: 181) **^{1}H NMR** (300 MHz, DMSO-d_6) δ 9.67 (s, 2H), 8.57 (s, 2H), 7.45 (d, J = 1.8 Hz, 2H), 7.25 (dd, J = 8.0, 1.9 Hz, 2H), 6.87 (d, J = 8.0 Hz, 2H), 3.83 (s, 6H) ppm.

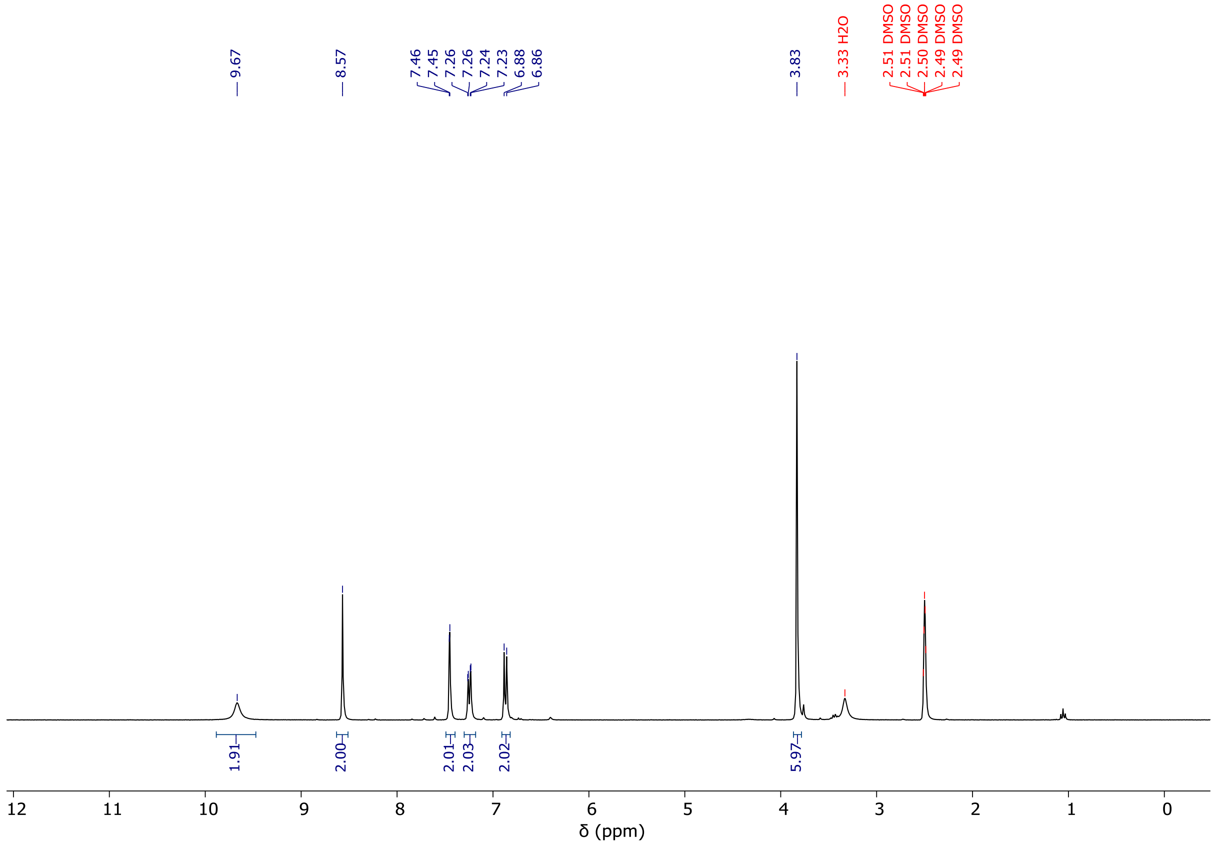

Figure S9: ^{1}H NMR spectrum of compound **EE**

1,2-bis((E)-4-methylbenzylidene)hydrazine (FF)[1]

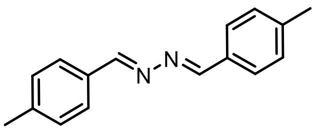

p-Tolualdehyde (1 mL, 8.3 mmol, 2 eq) was added dropwise over a solution of hydrazine monohydrate (0.20 mL, 4.1 mmol, 1 eq) in ethanol (25 mL) and stirred for 30 minutes. A precipitate was formed immediately. The precipitate was filtrated and washed with ethanol to give an as yellow solid in 82% isolated yield (0.8 g, 3.2 mmol). **^{1}H NMR** (400 MHz, $CDCl_3$): δ = 8.64 (s, 2H), 7.73 (d, J = 7.9 Hz, 4H), 7.26 (d, J = 7.9 Hz, 4H), 2.41 (s, 6H) ppm. **^{13}C NMR** (100 MHz, $CDCl_3$): δ = 162.0, 141.8, 131.6, 129.7, 128.7, 21.8 ppm.

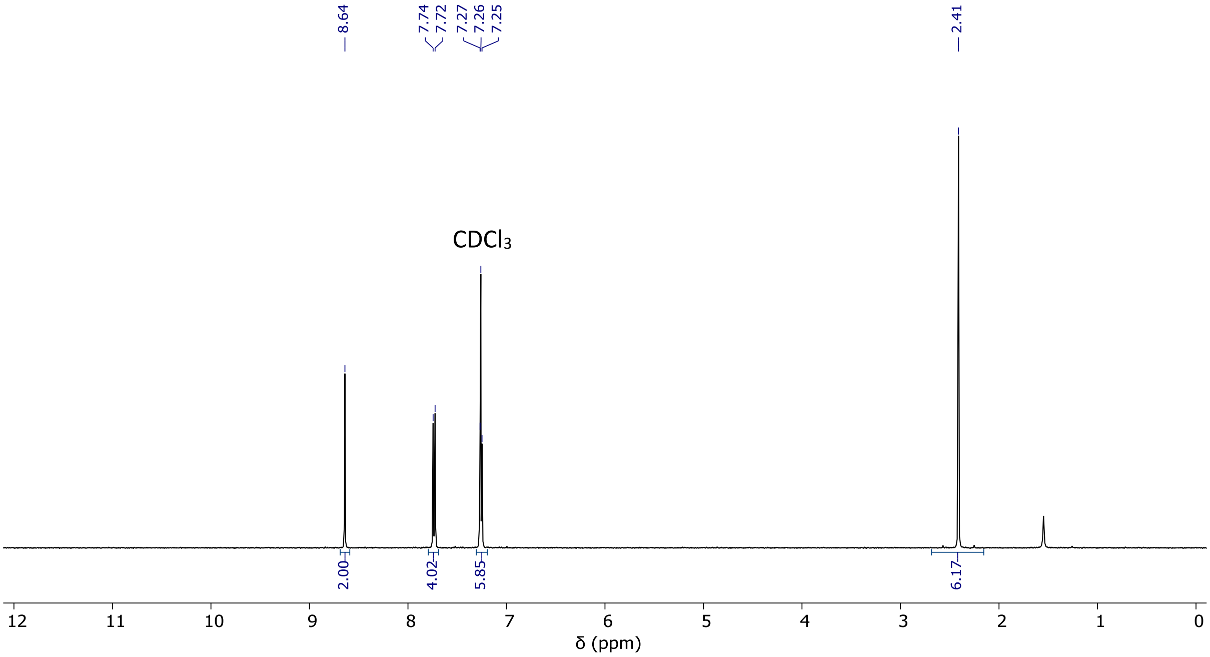

Figure S10: ^{1}H NMR spectrum of compound **FF**.

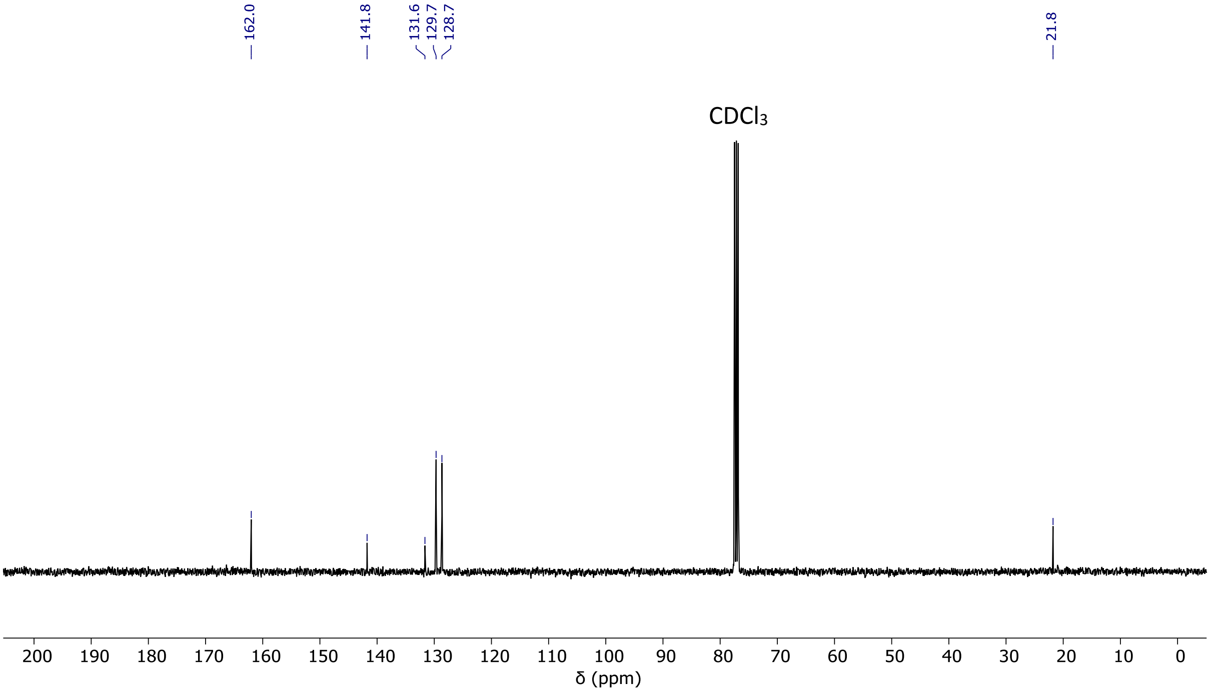

Figure S11: ^{13}C NMR spectrum of compound **FF**.

1,2-bis((E)-2-bromobenzylidene)hydrazine (GG)[5]

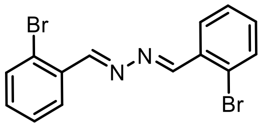

2-Bromobenzaldehyde (0.315 mL, 2.7 mmol, 2 eq) was added dropwise over a solution of hydrazine monohydrate (0.067 mL, 1.35 mmol 1 eq) in 25 mL ethanol, and stirred for 30 minutes. A precipitate was formed immediately. The precipitate was filtrated and washed with ethanol to give an as yellow solid in 85% isolated yield (0.42 g, 1.15 mmol). **^{1}H NMR** (400 MHz, $CDCl_3$) δ = 9.03 (s, 2H), 8.22 (dd, J = 7.8, 1.8 Hz, 2H), 7.63 (dd, J = 7.9, 1.3 Hz, 2H), 7.42 – 7.38 (m, 2H), 7.34 - 7.32 (m, 2H) ppm. **^{13}C NMR** (100 MHz, $CDCl_3$): δ = 161.5, 133.5, 133.1, 132.6, 128.9, 127.8, 126.0 ppm.

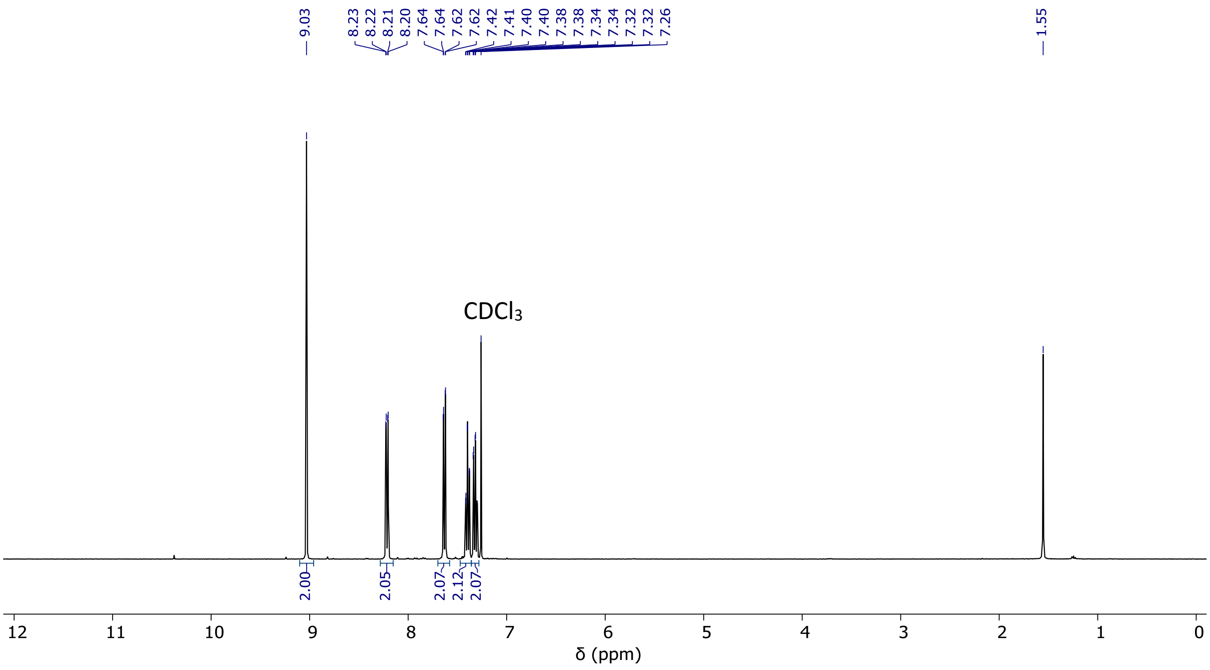

Figure S12: ^{1}H NMR spectrum of compound **GG**.

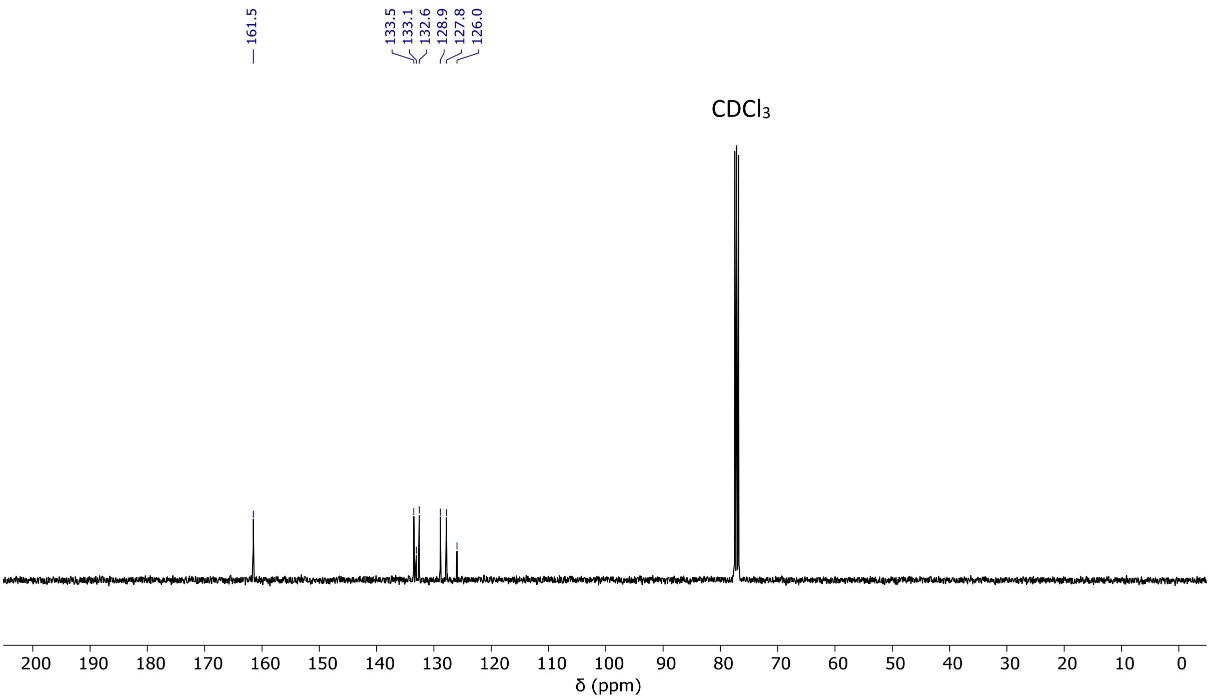

Figure S13: ^{13}C NMR spectrum of the compound **GG**.

4. Calculation of molar ratios

The percentage of the molar ratios was calculated as described in equations 1-3. For instance, in a mixture of aldehydes **X** and **Y** and azines **XX**, **YY**, and **XY**, the area (A) of the selected peak corresponding to each molecule is divided by the number (N) of protons contributing to the integral (1 for aldehydes, 2 for azines). After that, the total area (A_{total}) was calculated as follows:

$$\mathbf{A_{total} = A_X/N_X + A_Y/N_Y + A_{XX}/N_{XX} + A_{YY}/N_{YY} + A_{XY}/N_{XY}}$$
$$\mathbf{= A_X + A_Y + A_{XX}/2 + A_{YY}/2 + A_{XY}/2} \qquad \textit{Equation 1}$$

For aldehyde **X** and azine **XX**, the mol percentages would be as follows:

Aldehyde **X:** $\mathbf{mol\% = (A_X/A_{total})*100}$ *Equation 2*

Azine **XX:** $\mathbf{mol\% = (½\ A_{XX}/A_{total})*100}$ *Equation 3*

5. Azines stability in acidic conditions and aqueous solutions

For the formation of **AA** in acid conditions were mixed: **A** (2 eq., 28 mM), hydrazine hydrate (1 eq., 14 mM), and trifluoroacetic acid (TFA) (1 eq.). For the hydrolysis reactions of **AA** in acid conditions were mixed: **AA** (1 eq., 14 mM) and TFA (1 eq.). The DMSO-d_6/D_2O ratio of the solvent was varied to evaluate the azine stability in the presence of water. Table S1 summarizes the most relevant results.

Table S1*:* Reaction conditions and most relevant results assessed during the formation and hydrolysis of the **AA** azine.

Entry		Acid (Eq.)	Solvent	Time (hours)	A	AA
1	**AA** hydrolysis	n/a	DMSO-d_6 / D_2O (60:40)	1	0	100
				7 days	0	100
		TFA (1)	DMSO-d_6	1	0	100
				72	1	99
		TFA (1)	DMSO-d_6 / D_2O (95:5)	1	6	94
				72	7	93
		TFA (1)	DMSO-d_6 / D_2O (80:20)	1	16	84
				72	16	84
2	**AA** formation	TFA (1)	DMSO-d_6	1	2	98
				72	0	100
		TFA (1)	DMSO-d_6 / D_2O (95:5)	1	7	93
				72	5	95
		TFA (1)	DMSO-d_6 / D_2O (80:20)	1	11	89
				72	10	90

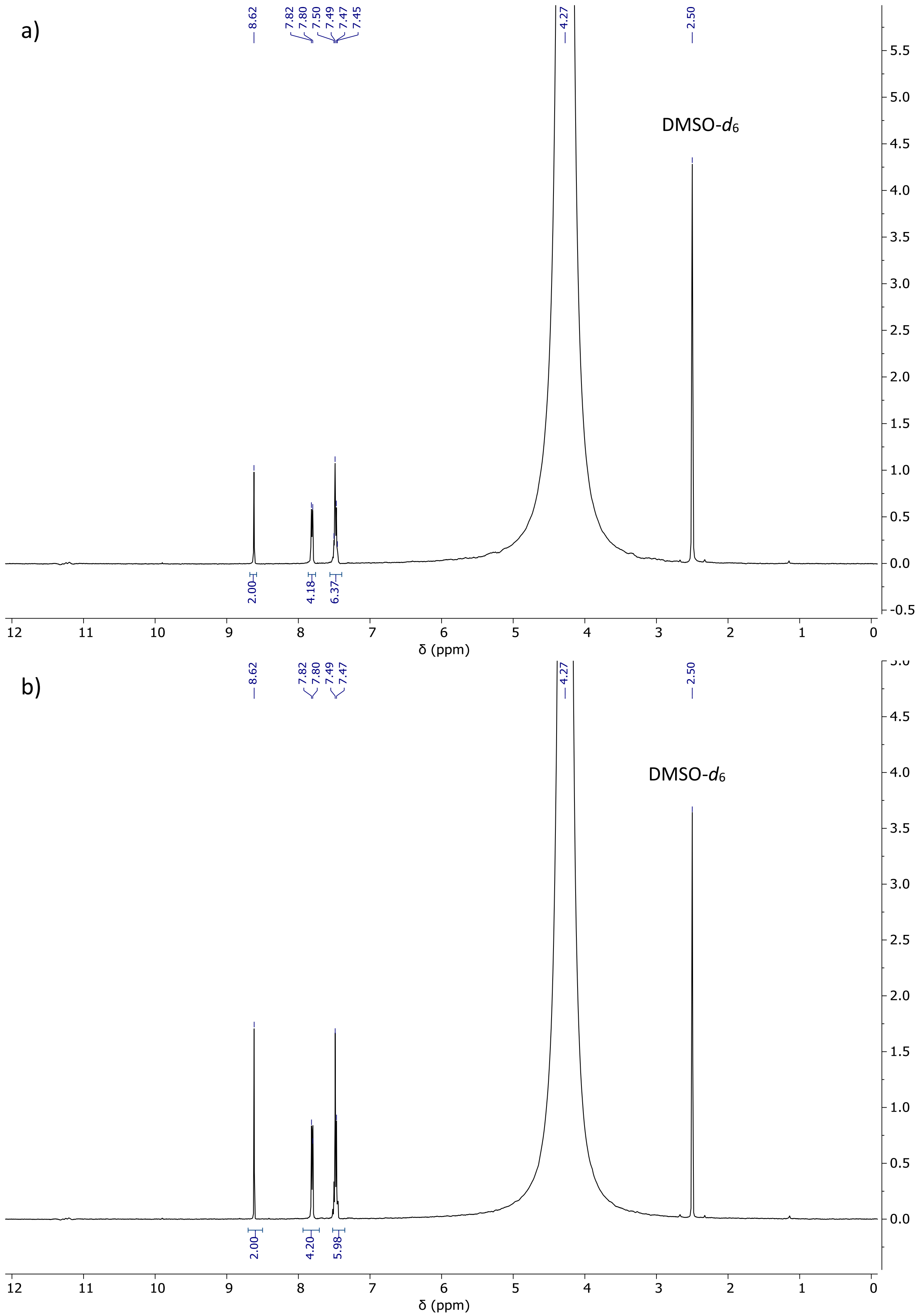

Figure S14: ^{1}H NMR (400 MHz, DMSO-d_6/D_2O, 60:40) spectra of **AA** azine (14 mM), after a) 1 hour and b) 7 days.

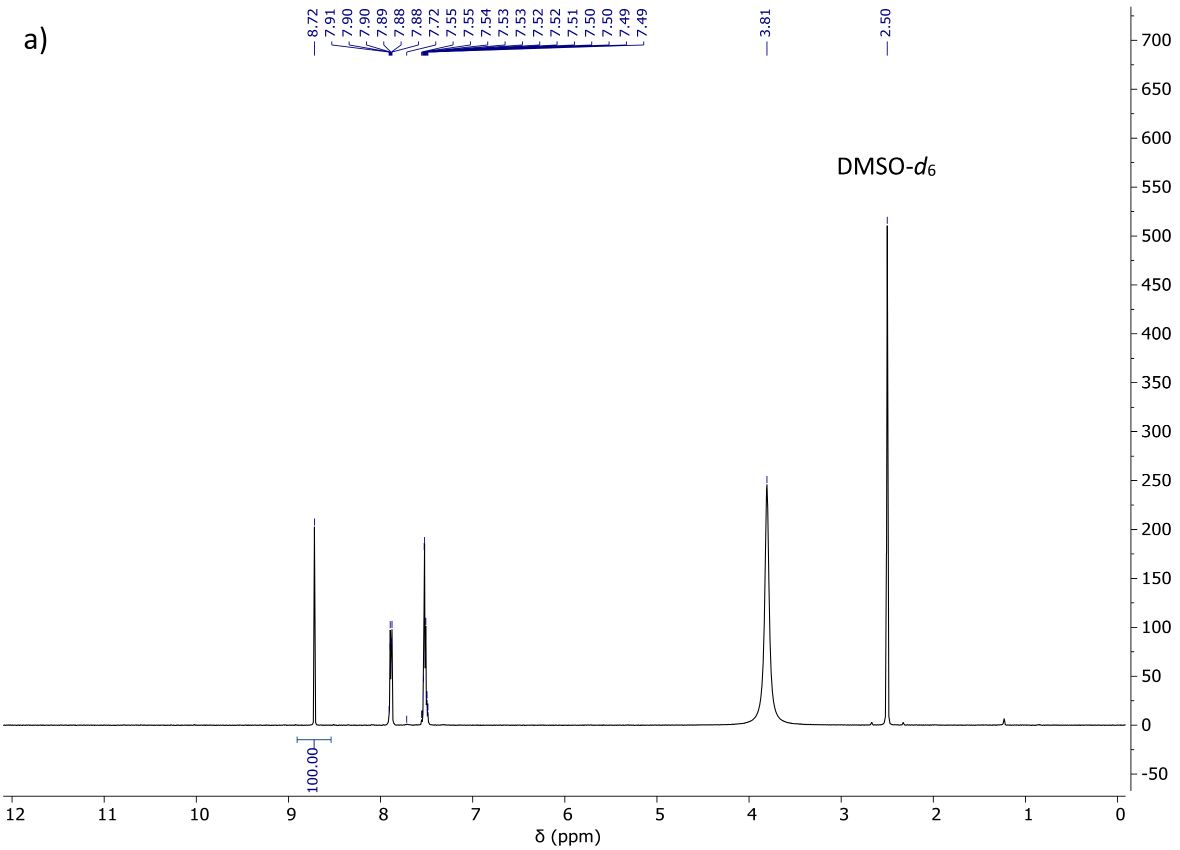

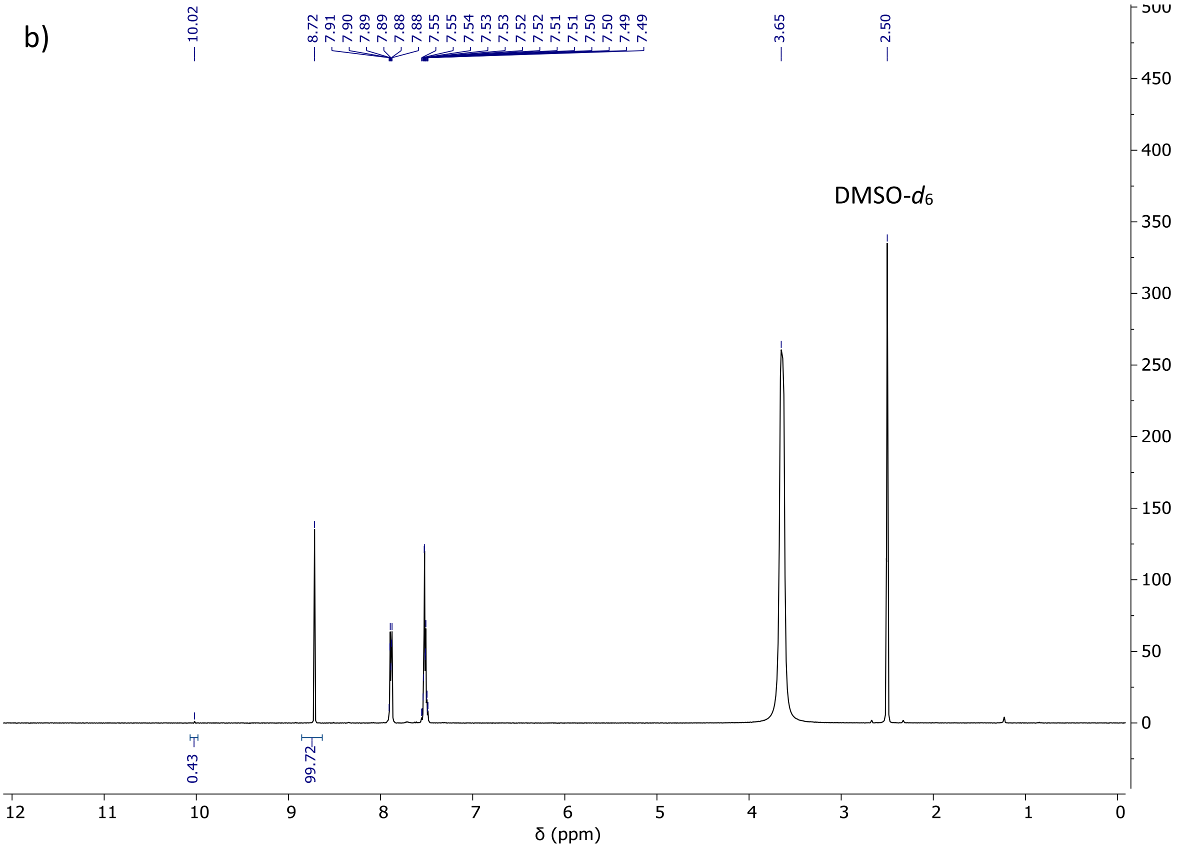

Figure S15: ^{1}H NMR (400 MHz, DMSO-d_6) spectra of **AA** hydrolysis (14 mM) in TFA (1 eq.), after a) 1 hour and b) 72 hours.

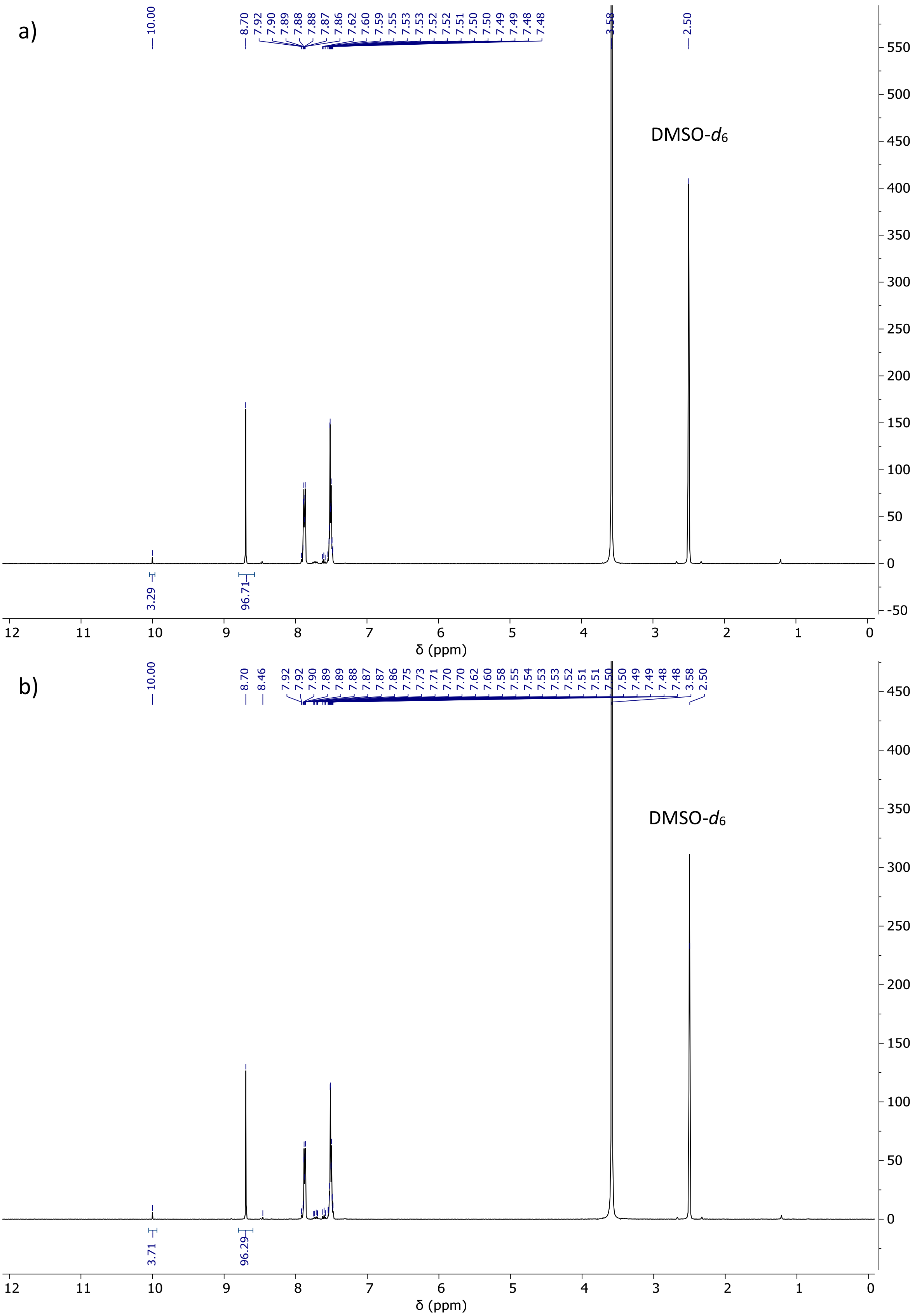

Figure S16: ^{1}H NMR (400 MHz, DMSO-d_6/D_2O, 95:5) spectra of **AA** hydrolysis (14 mM) in TFA (1 eq.), after a) 1 hour and b) 72 hours.

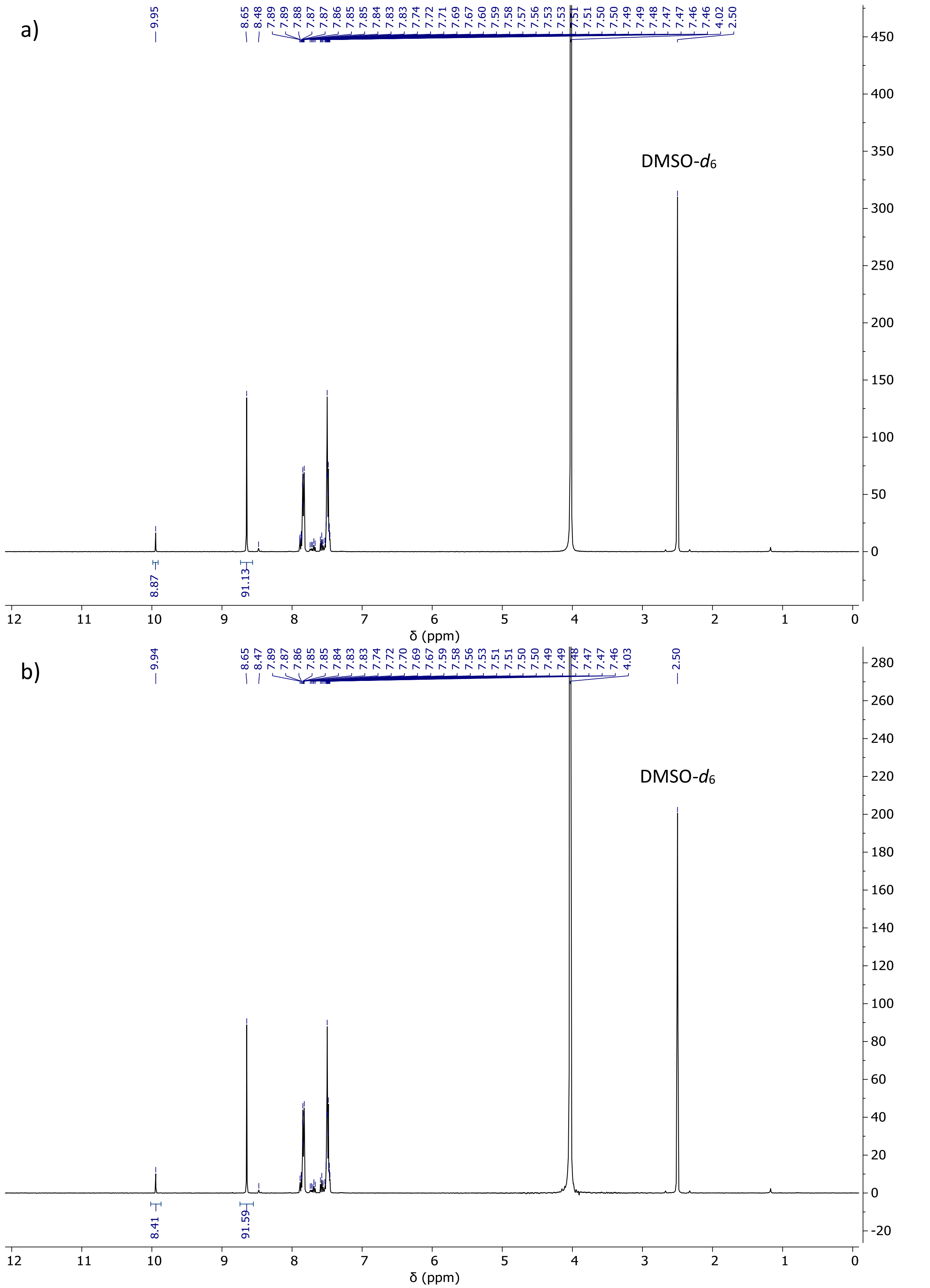

Figure S17: ^{1}H NMR (400 MHz, DMSO-d_6/D_2O, 80:20) spectra of **AA** hydrolysis (14 mM) in TFA (1 eq.), after a) 1 hour and b) 72 hours.

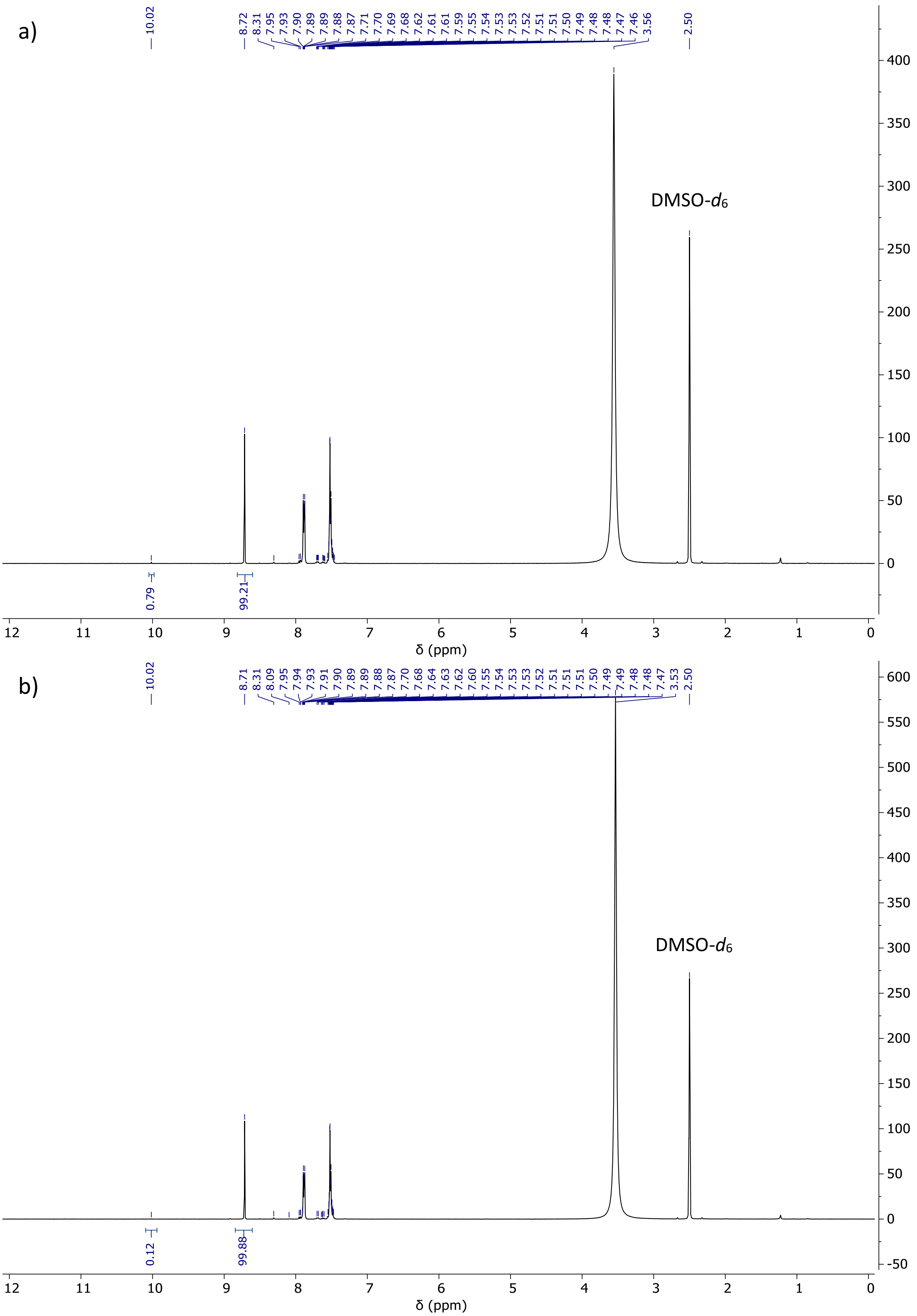

Figure S18: ^{1}H NMR (400 MHz, DMSO-d_6) spectra of **AA** formation (14 mM) in TFA (1 eq.), after a) 1 hour and b) 72 hours.

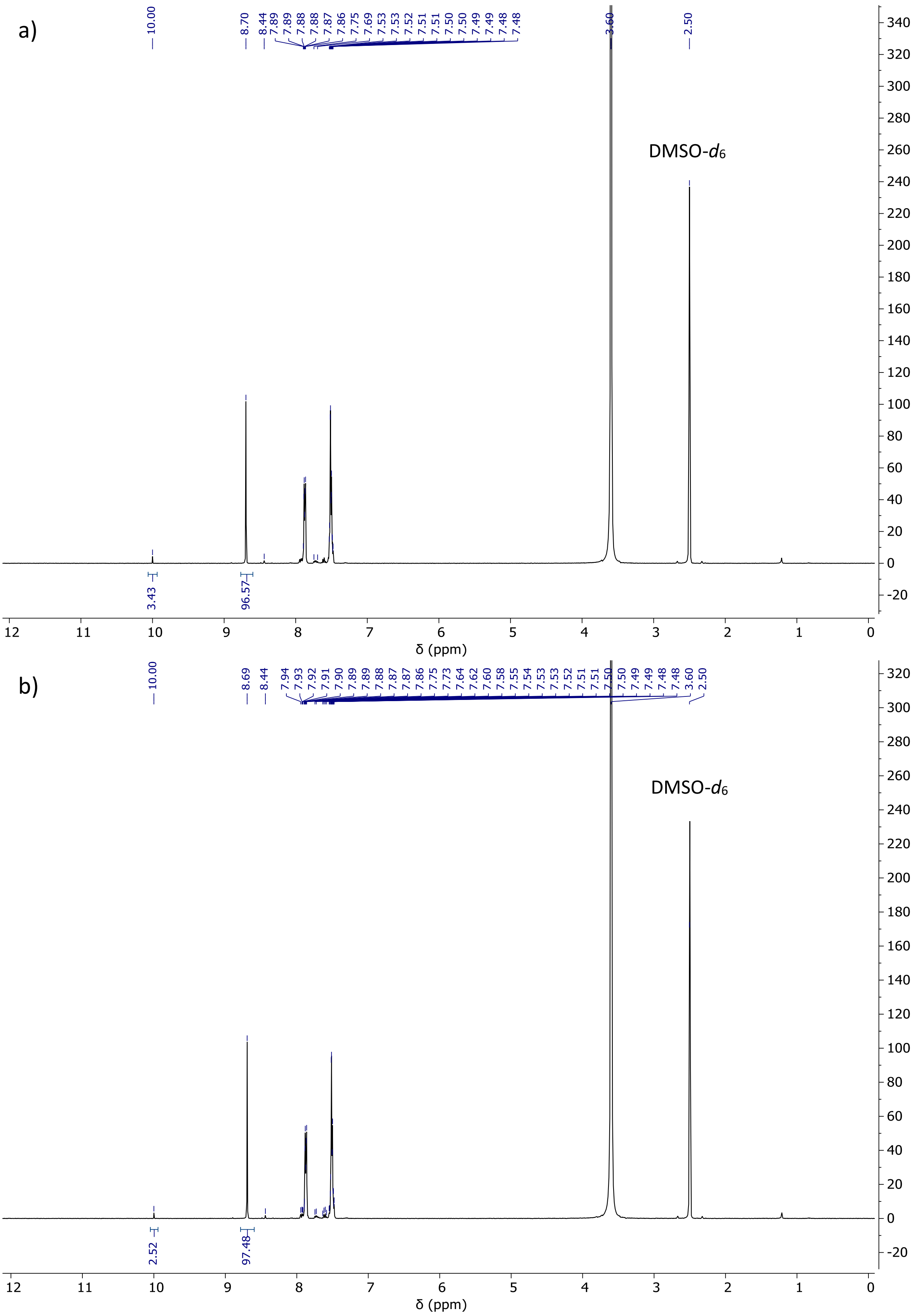

Figure S19: ^{1}H NMR (400 MHz, DMSO-d_6/D_2O, 95:5) spectra of **AA** formation (14 mM) in TFA (1 eq.), after a) 1 hour and b) 72 hours.

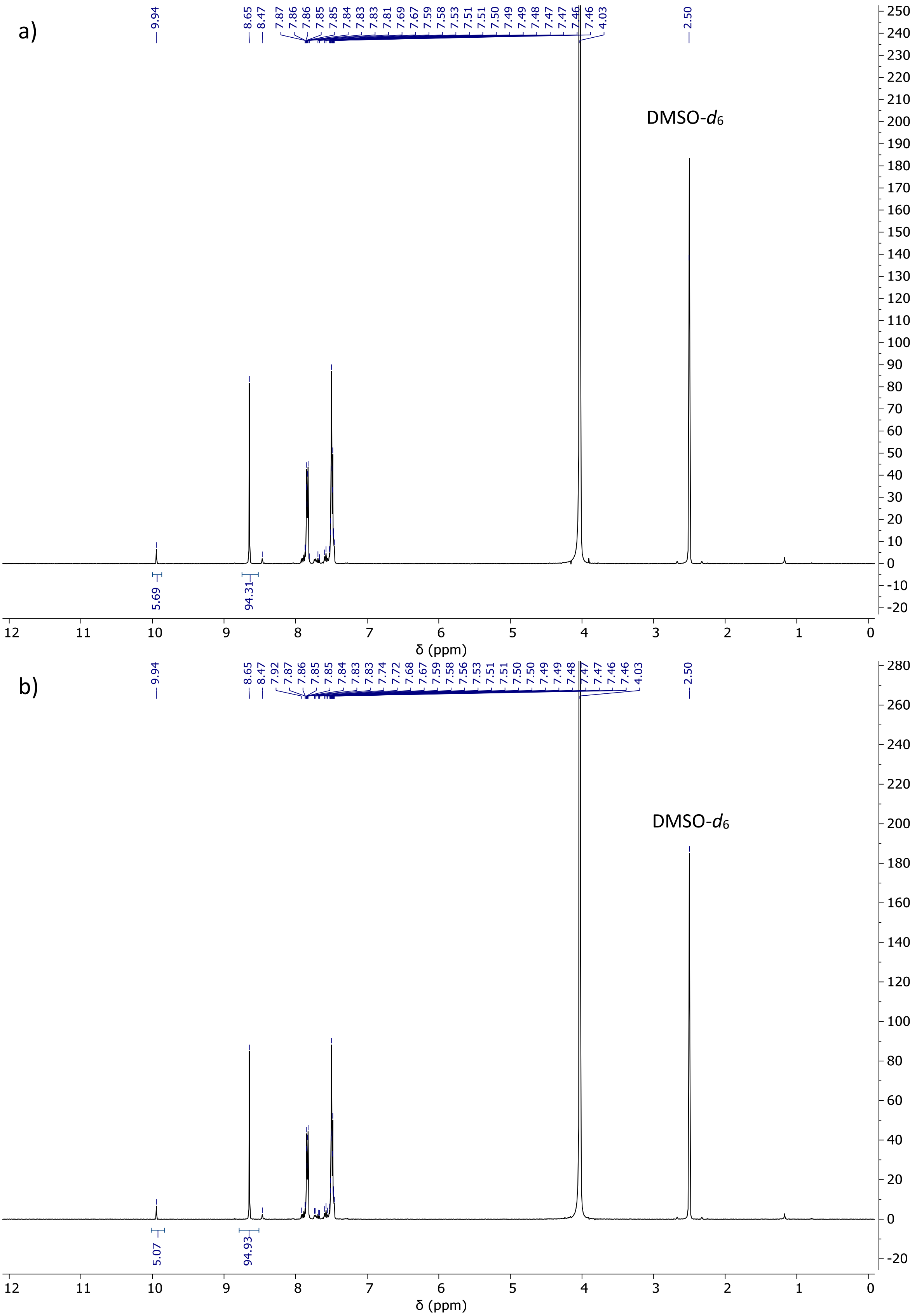

Figure S20: ^{1}H NMR (400 MHz, DMSO-d_6/D_2O, 80:20) spectra of **AA** formation (14 mM) in TFA (eq.), after a) 1 hour and b) 72 hours.

Hydrolysis of CC and DD compounds

For the hydrolysis reactions of **CC** and **DD** in acid conditions were mixed: **CC** or **DD** (1 eq., 7 mM) and TFA (1 eq.) in DMSO-d_6/D_2O (95/5). Table S2 summarizes the most relevant results.

Table S2: Reaction conditions and most relevant results assessed during the hydrolysis of azines **CC** and **DD**.

Entry		Acid (Eq.)	Solvent	Time (hours)	Aldehyde	Azine
**1**	**CC** hydrolysis	TFA (1)	DMSO-d_6 / D_2O (95:5)	1	4	96
				72	6	94
**2**	**DD** hydrolysis	TFA (1)	DMSO-d_6 / D_2O (95:5)	1	7	93
				72	17	83

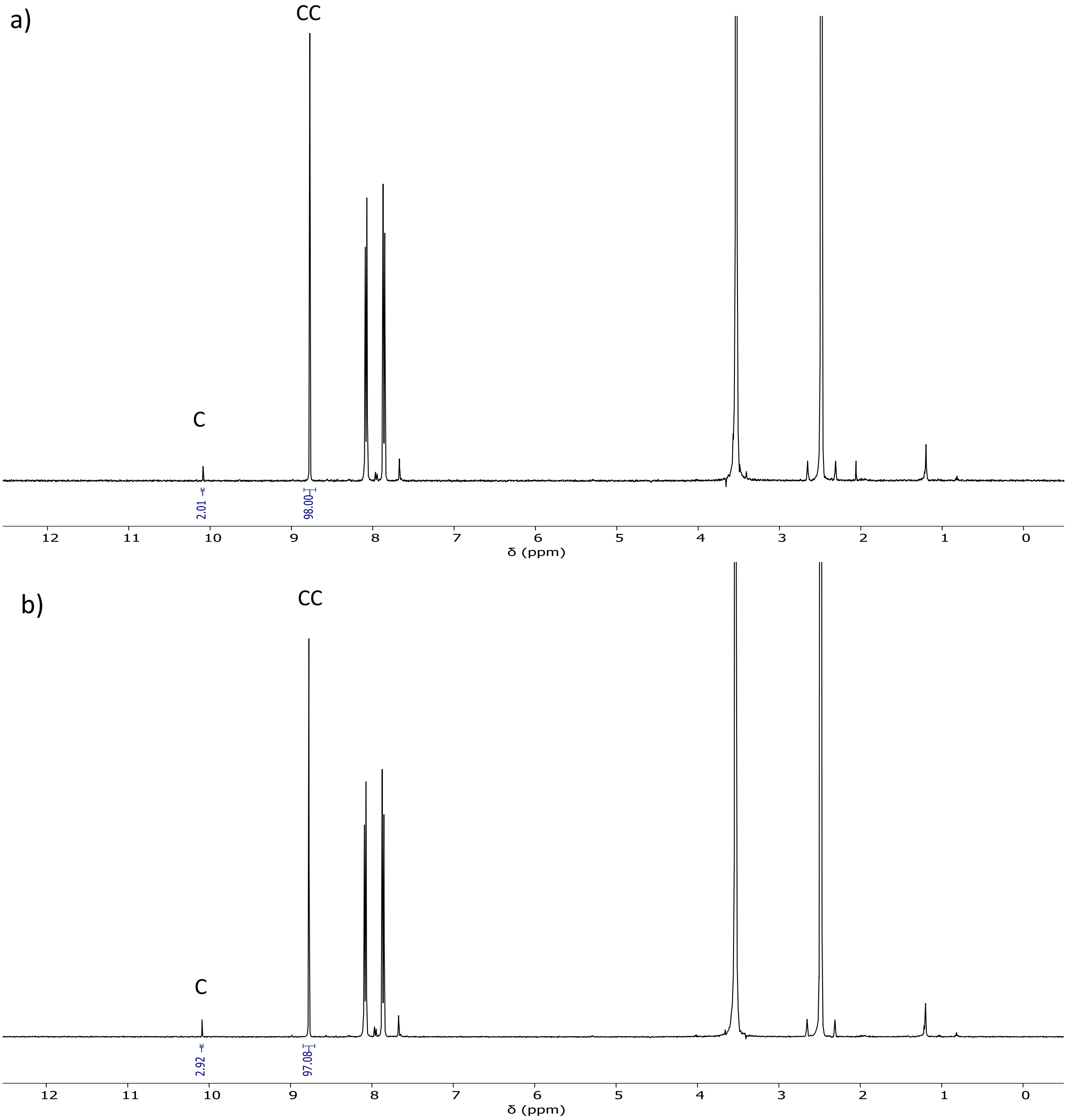

Figure S21: ^1H NMR (400 MHz, DMSO-d_6/D_2O, 95:5) spectra of **CC** hydrolysis (7 mM) in the presence of TFA (1 eq.), after a) 1 hour and b) 72 hours.

a)

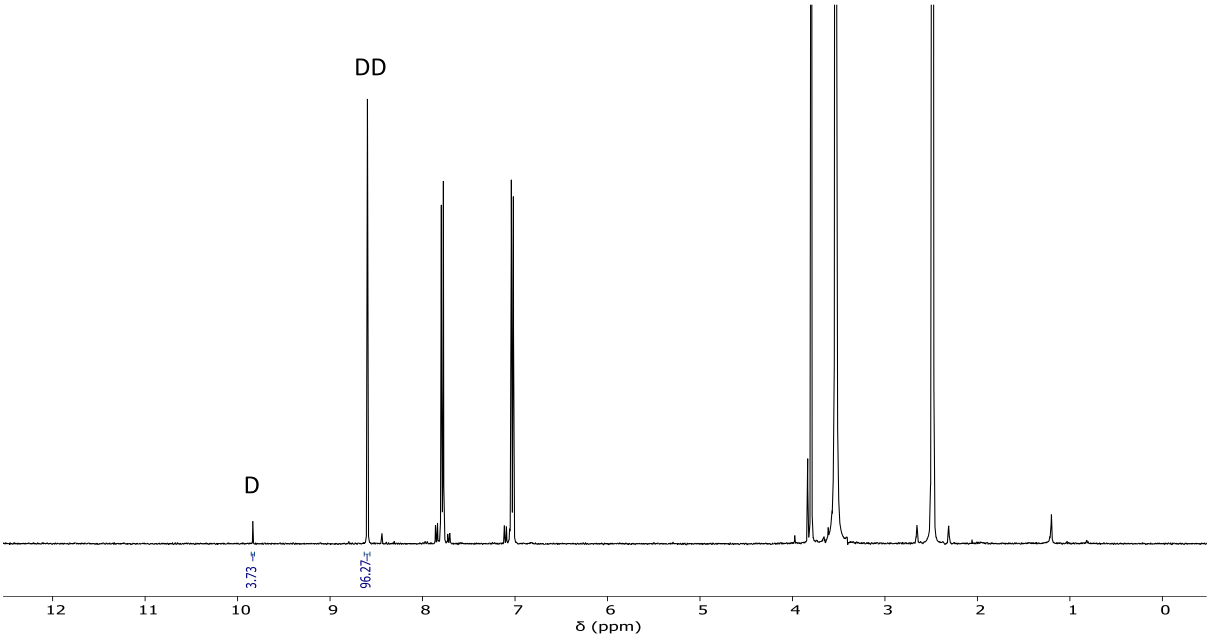

b)

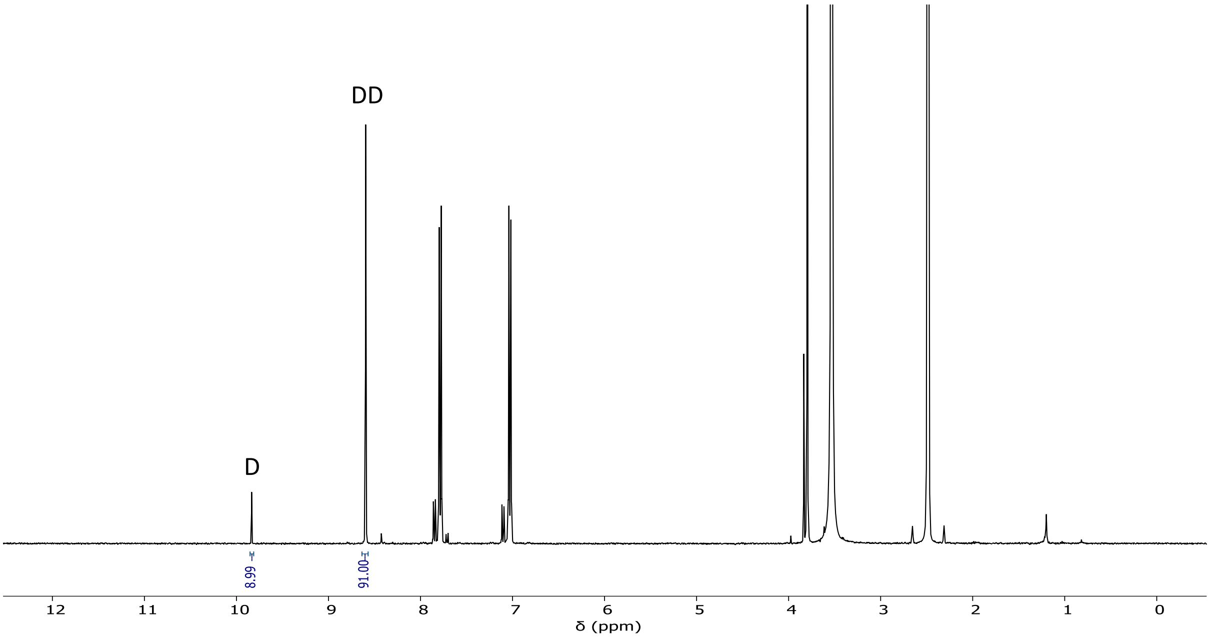

Figure S22: ^{1}H NMR (400 MHz, DMSO-d_6/D_2O, 95:5) spectra of **DD** hydrolysis (7 mM) in the presence of TFA (1 eq.), after a) 1 hour and b) 72 hours.

Hydrolysis of azine EE

Hydrolysis reactions of **EE** was monitored in D_2O/ DMSO-d_6 (80/20) buffered at different pD's. 1 mM solutions of EE in appropriately buffered D_2O/ DMSO-d_6 was formulated as follows: A stock solution of azine **EE** (50 mM, DMSO-d_6) was added to D_2O/DMSO-d_6 (buffered to appropriate pD with 50 mM McIlvaine buffer and containing 18.36% DMSO-d_6) to give a 1 mM azine solution and these samples were then monitored for 6 days.

McIlvaine buffer was formulated by mixing appropriate amounts of a 0.2 M stock solution of Na_2HPO_4 and 0.1 M stock solution of citric acid and ensuring correct pD (pH+0.41) was attained by measurement with a pH probe.[6]

Table S3 summarizes the mol % of **E** (aldehyde) **EE** (azine) and **E$_H$** (hydrazone) present at each pD value at the specified times.

Table S3: Reaction conditions and most relevant results assessed during the hydrolysis of azine **EE**

	pD	Solvent	Time	E	EE	E_H
**EE** hydrolysis	pD = 8	D_2O/DMSO-d_6 (80:20)	1 hour	0	100	0
			3 days	13	87	0
			6 days	21	79	0
	pD = 7	D_2O/DMSO-d_6 (80:20)	1 hour	0	100	0
			3 days	22	62	16
			6 days	31	50	19
	pD = 6	D_2O/DMSO-d_6 (80:20)	1 hour	2	97	1
			3 days	34	50	17
			6 days	33	50	16
	pD = 5	D_2O/DMSO-d_6 (80:20)	1 hour	8	86	6
			3 days	57	36	6
			6 days	61	33	6

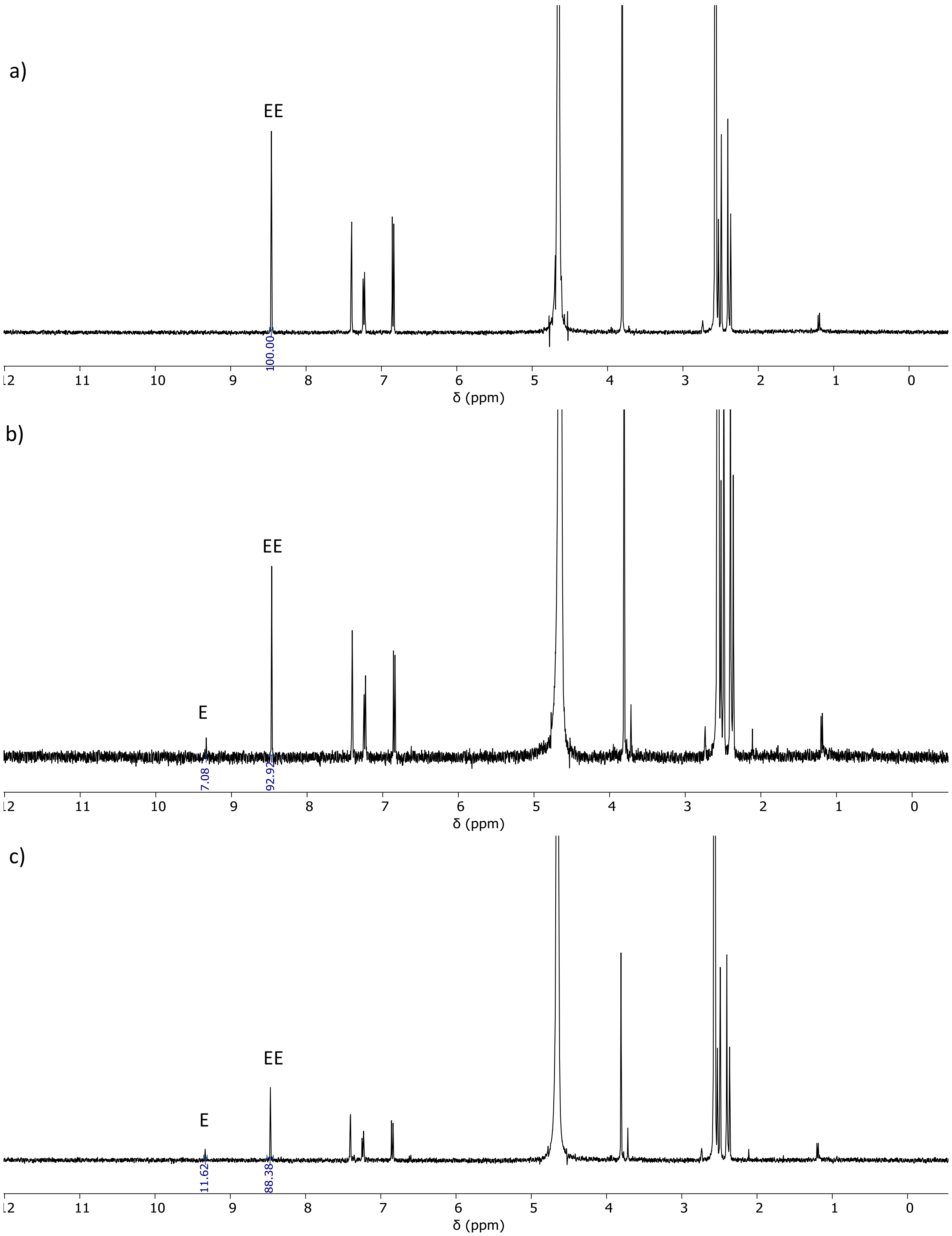

Figure S23: ^{1}H NMR (400 MHz, D_2O/DMSO-d_6, 80:20) spectra of **EE** hydrolysis (1 mM) at pD = 8 (50 mM McIlvaine buffer), after a) 1 hour and b) 72 hours, c) 144 hours.

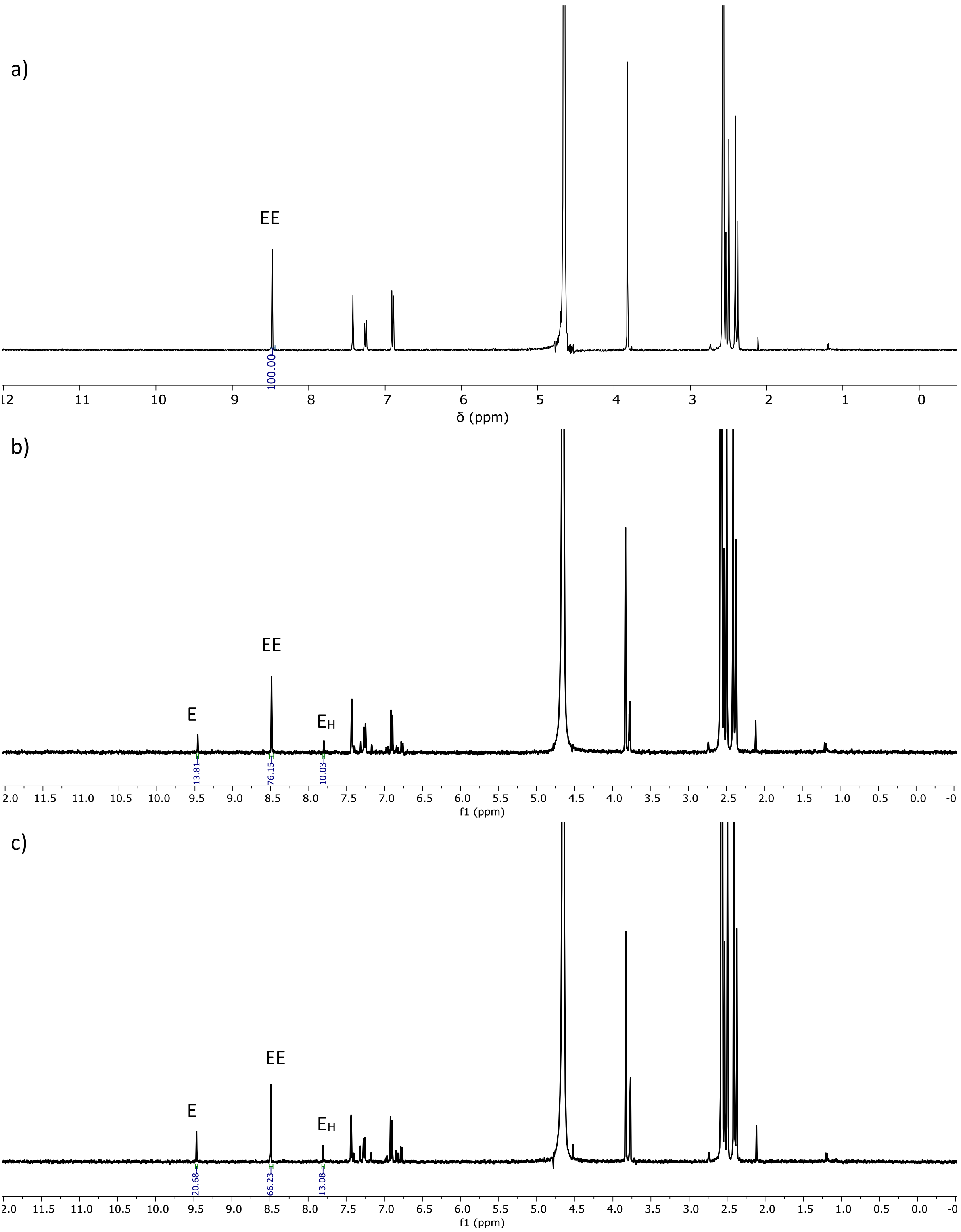

Figure S24: ^{1}H NMR (400 MHz, D_2O/DMSO-d_6, 80:20) spectra of **EE** hydrolysis (1 mM) at pD = 7 (50 mM McIlvaine buffer), after a) 1 hour and b) 72 hours, c) 144 hours.

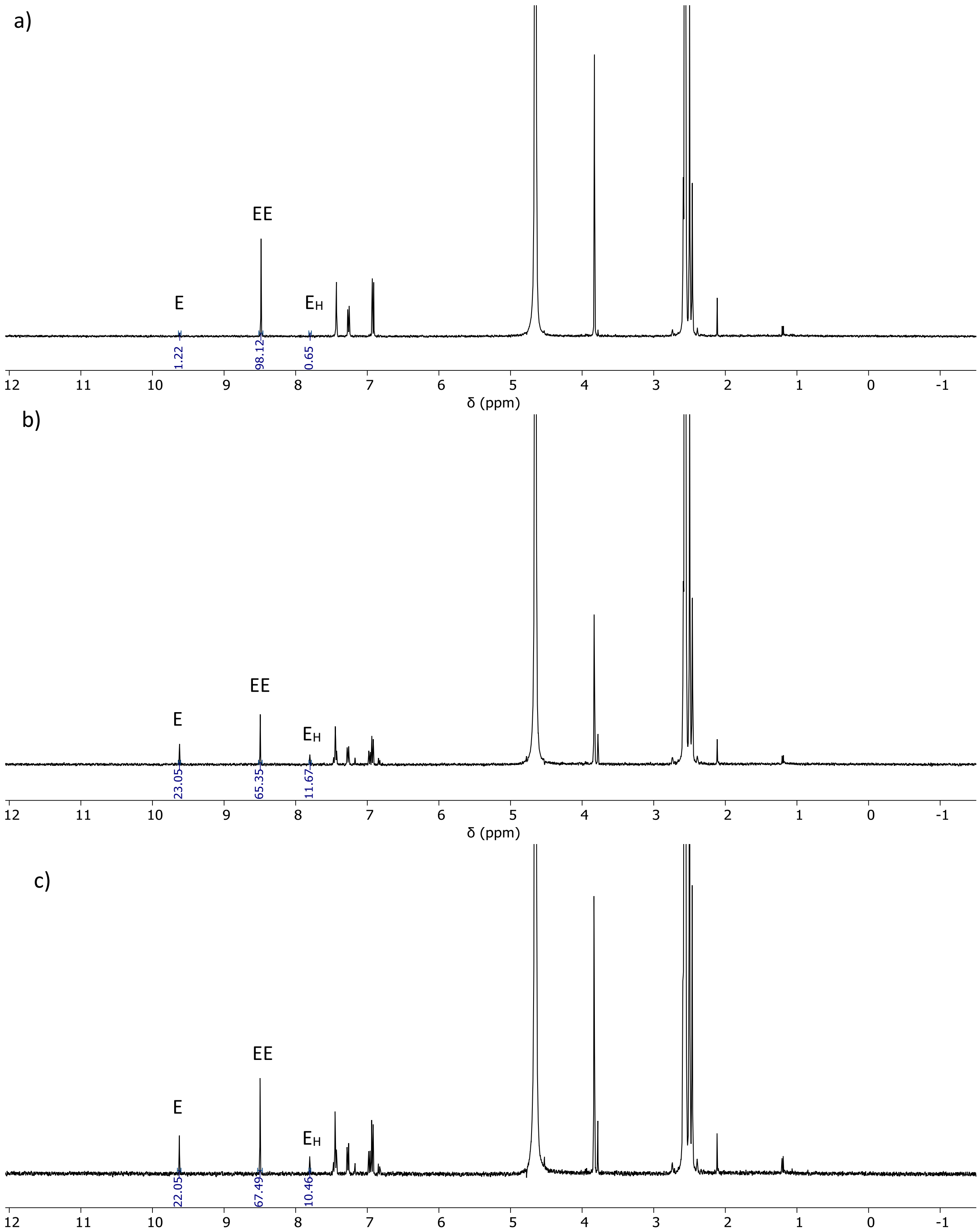

Figure S25: ^{1}H NMR (400 MHz, D_2O/DMSO-d_6, 80:20) spectra of **EE** hydrolysis (1 mM) at pD = 6 (50 mM McIlvaine buffer), after a) 1 hour and b) 72 hours, c) 144 hours.

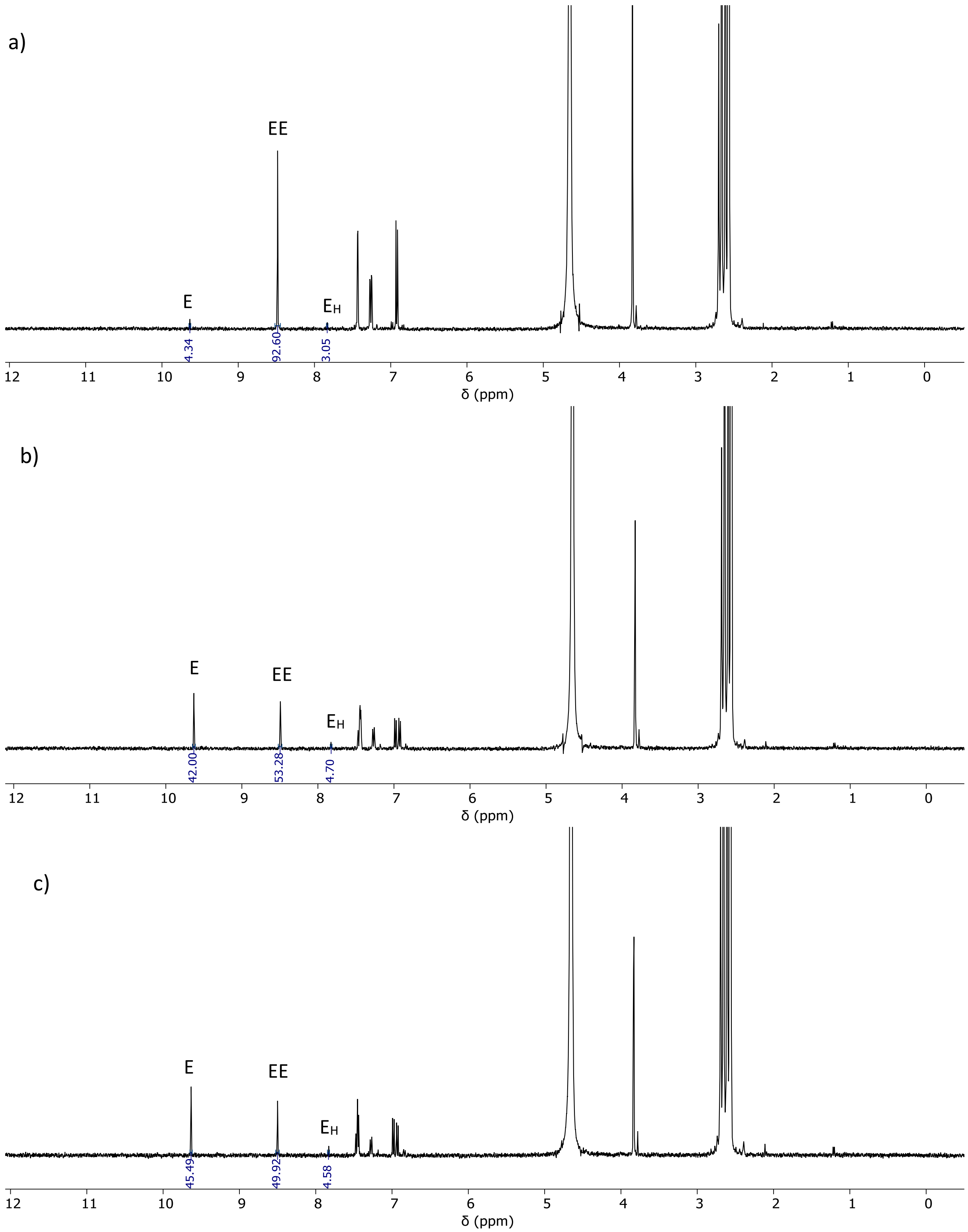

Figure S26: ^{1}H NMR (400 MHz, D_2O/DMSO-d_6, 80:20) spectra of **EE** hydrolysis (1 mM) at pD = 5 (50 mM McIlvaine buffer), after a) 1 hour and b) 72 hours, c) 144 hours.

6. Imine stability in aqueous solution.

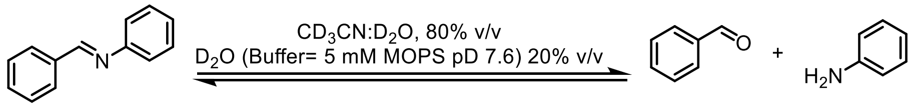

1 2 3

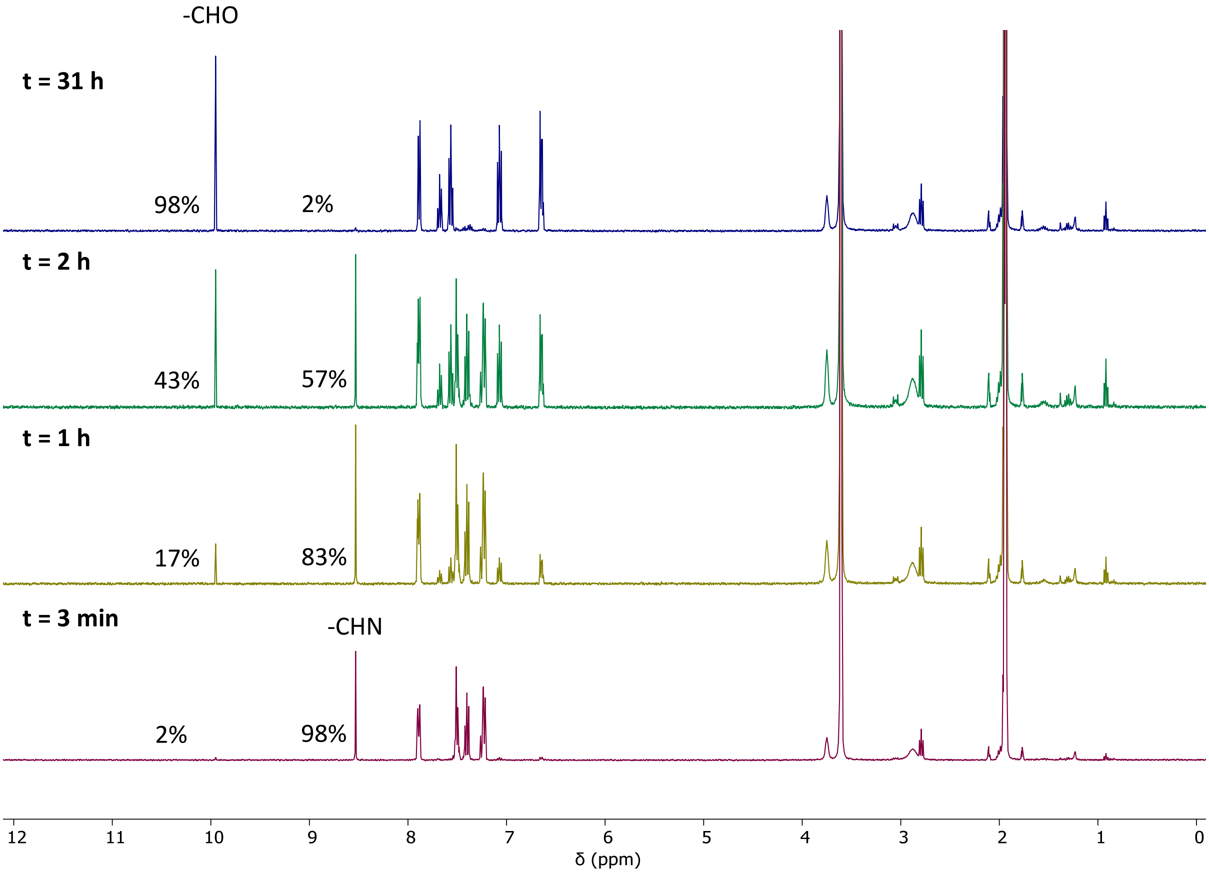

Figure S27: ^{1}H NMR spectra (400 MHz, CD_3CN) of benzylideneaniline (2 mM) in the presence of D_2O (20 % v/v) (pD = 7.6), after 3 min, 1 h, 2 h and 31 h.

7. Proof of reversibility

The equilibrium from different starting points was assessed to demonstrate that azine exchange is reversible. For this study, one equivalent of azine (7 mM), two equivalents of aldehyde 14 mM), and TFA (7 mM) in $CDCl_3$ were mixed in glass scintillation vials (2 mL). The progress of the reaction was monitored with ^{1}H NMR spectroscopy after 1 h, 24 h, and 7 days, showing no significant changes in the ratios over time, as demonstrated by the spectra after 1 h and 7 days (Figures S17-S18). The protons of the methine group (–CH=) from the corresponding azines and aldehydes were integrated and assigned using their corresponding letters.

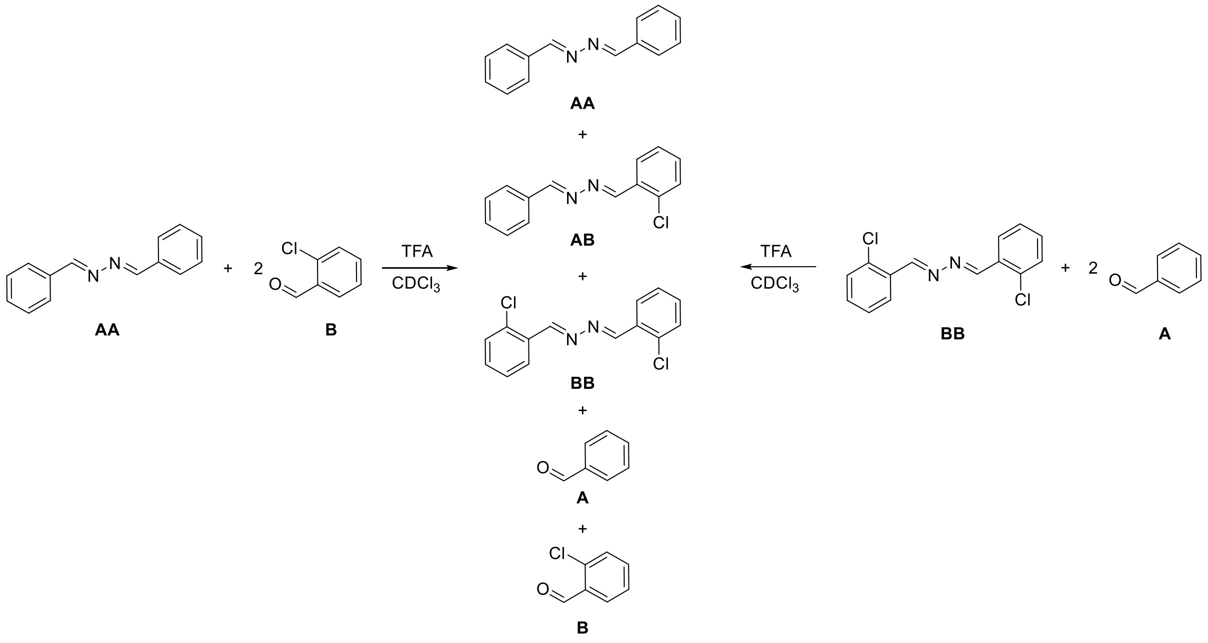

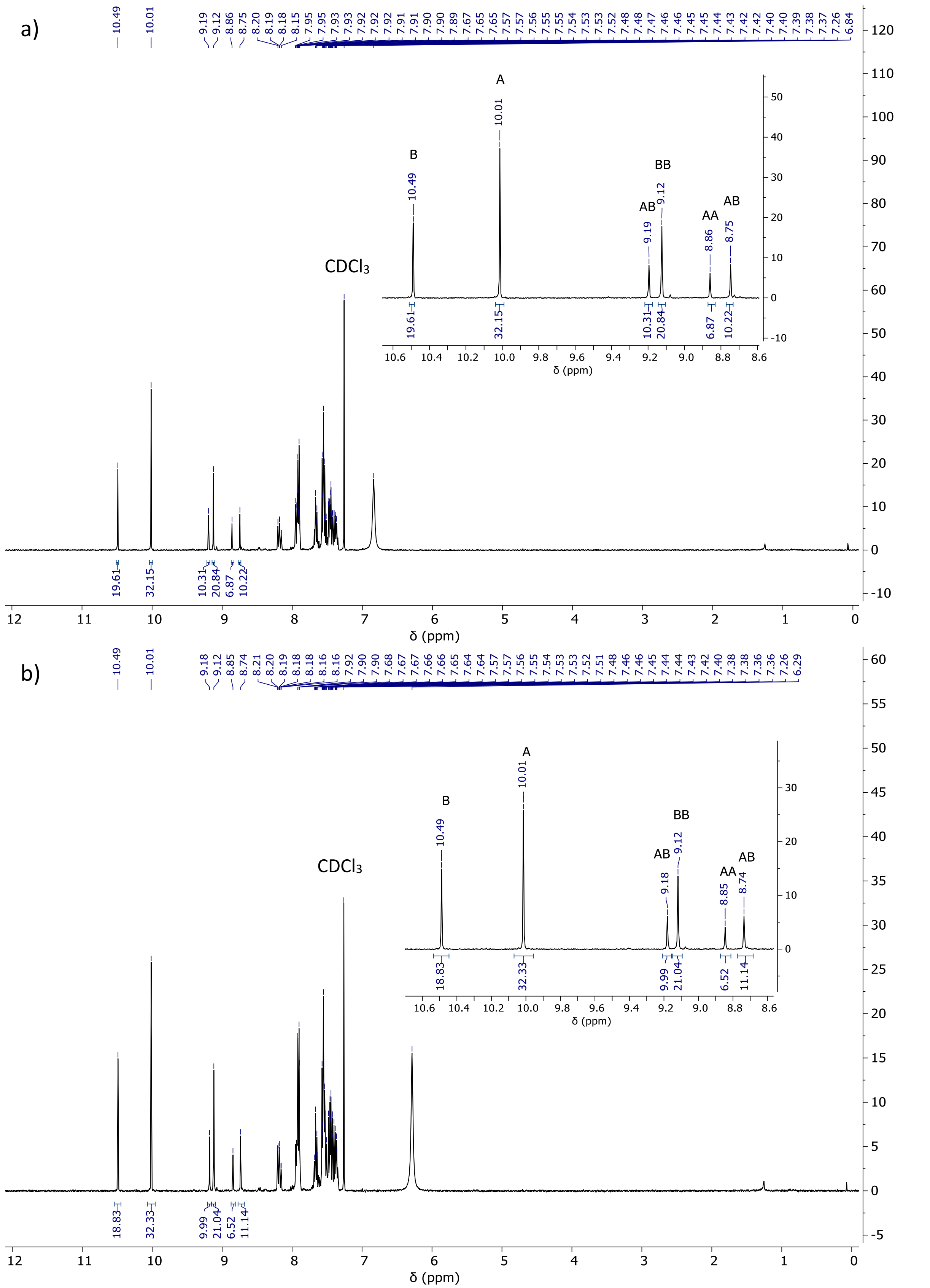

Figure S28: ^{1}H NMR (400 MHz, $CDCl_3$) spectra for the exchange reaction (**AA** + 2**B**) of the azine **AA** (7 mM) and the aldehyde **B** (14 mM) in TFA (1 eq.), after a) 1 hour and b) 7 days.

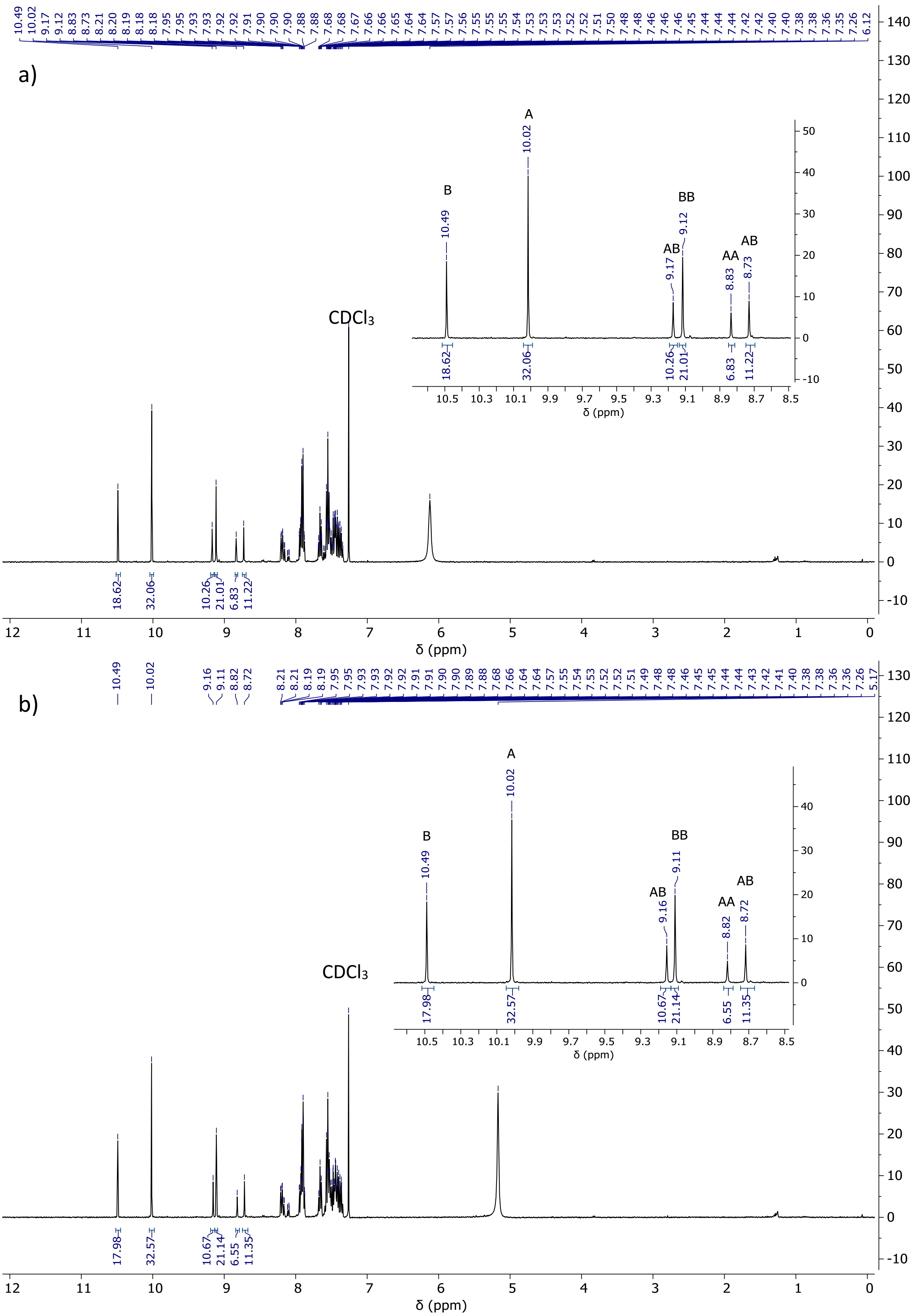

Figure S29: ^{1}H NMR (400 MHz, $CDCl_3$) spectra for the exchange reaction (**BB** + 2**A**) of azine **BB** (7 mM) and the aldehyde **A** (14 mM) in TFA (1 eq.), after a) 1 hour and b) 7 days.

8. Acylhydrazone control experiments

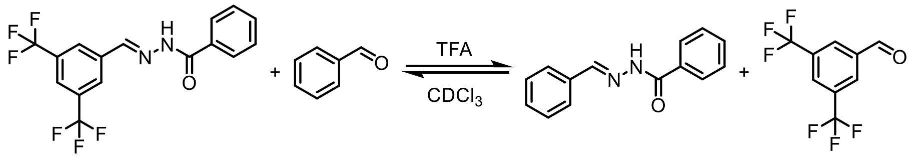

Acylhydrazone (**4**) and benzaldehyde (**2**) were mixed in equimolar amounts (7 mM, $CDCl_3$) and were subjected to the same exchange conditions as used for azine exchange (TFA, 1 eq.). The library was monitored by the ratio of aldehydes (**2** and **6**) until equilibrium was reached.

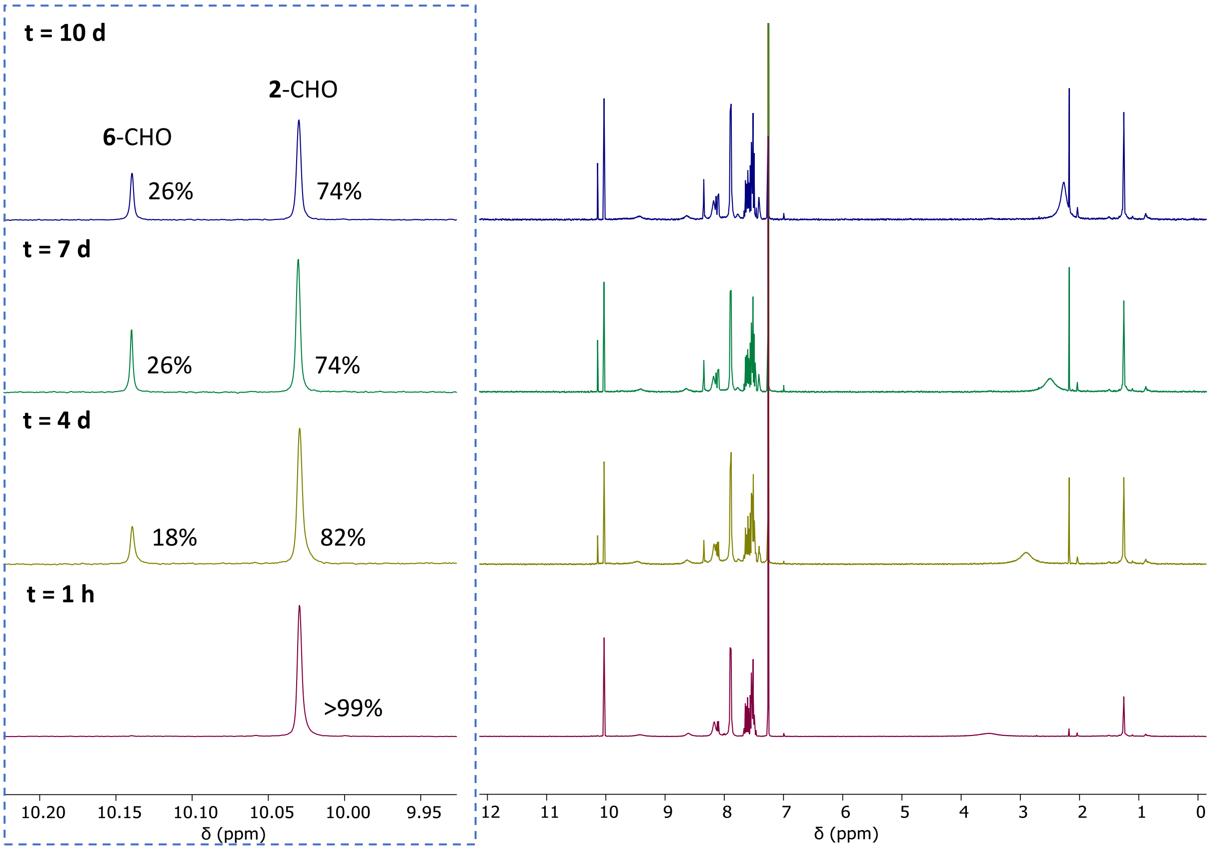

Figure S30: ^{1}H NMR (400 MHz, $CDCl_3$) spectra of a mixture of hydrazone **4** and benzaldehyde in the presence of 1 equivalent TFA after 1 h, 4 d, 7 d, and 10 d. Inset: expansion on the ^{1}H NMR spectra ranges from 9.95 ppm to 10.20 ppm.

9. Azine exchange reactions

In an typical experiment, azine **AA** (initial concentration: 1 eq., 7 µmol, 14 mM), aldehyde **B** (initial concentration: 1 eq., 7 µmol, 14 mM) were mixed in the presence of 1 equivalent (7 µmol, 14 mM) or 0.1 equivalent (0.7 µmol, 1.4 mM) of additive (trifluoroacetic acid, p-toluenesulfonic acid, acetic acid, formic acid) in deuterated solvent solutions (V_{total}: 500 µL). The reactions were carried out in 2 mL glass vials. After the addition of all reagents, the vials were shaken properly. The reaction progress was monitored by ^{1}H NMR spectroscopy after 1 h, 24 h, and 7 or 10 days. The protons of the methine group (–CH=) of the azines and aldehydes were integrated and assigned using their corresponding letters.

9.1 Effect of Brønsted acids

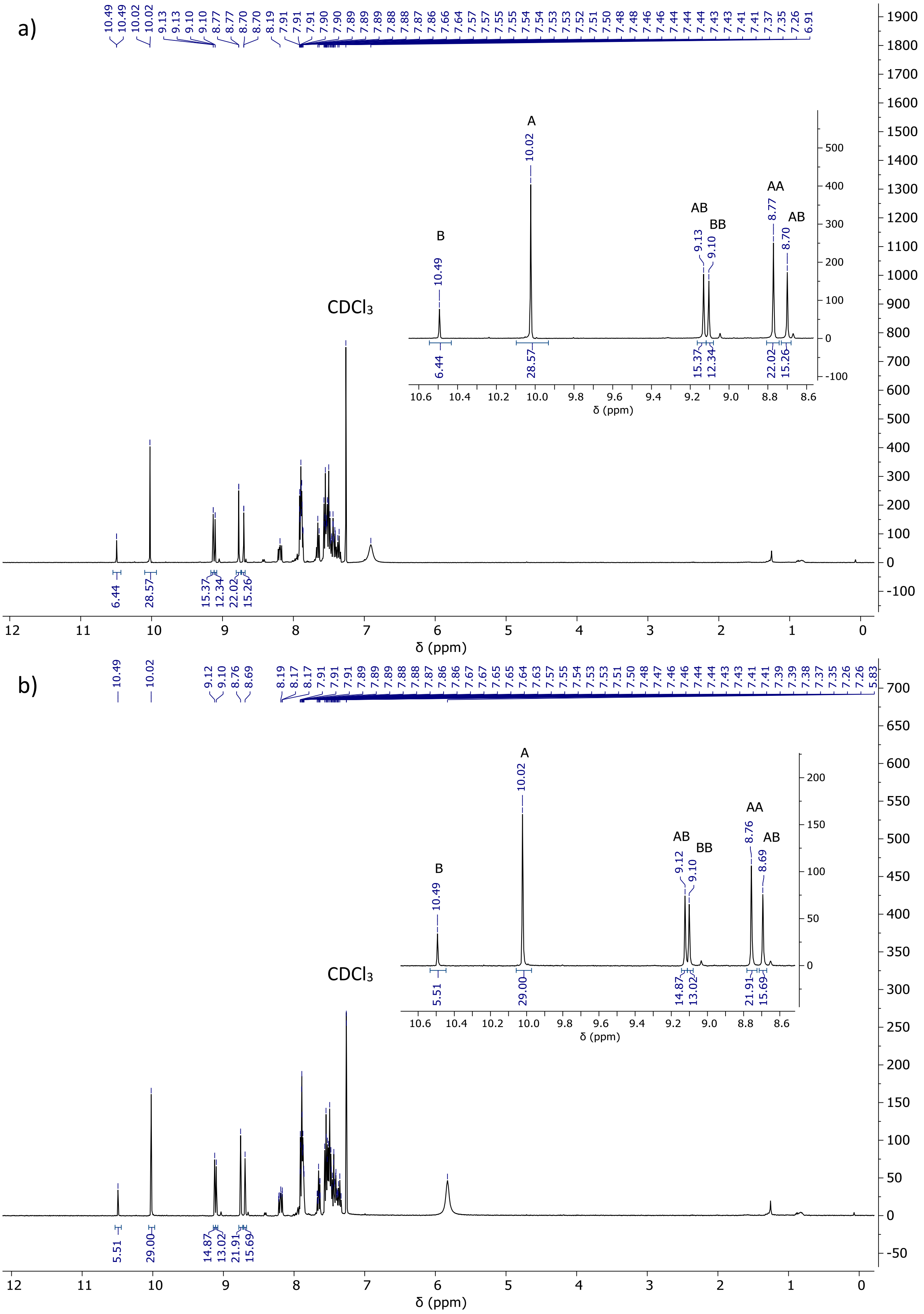

Figure S31: ^{1}H NMR (400 MHz, $CDCl_3$) spectra of equilibrium of **AA** + **B** (14 mM each), in the presence of TFA (1 eq.), after a) 1 hour and b) 24 hours.

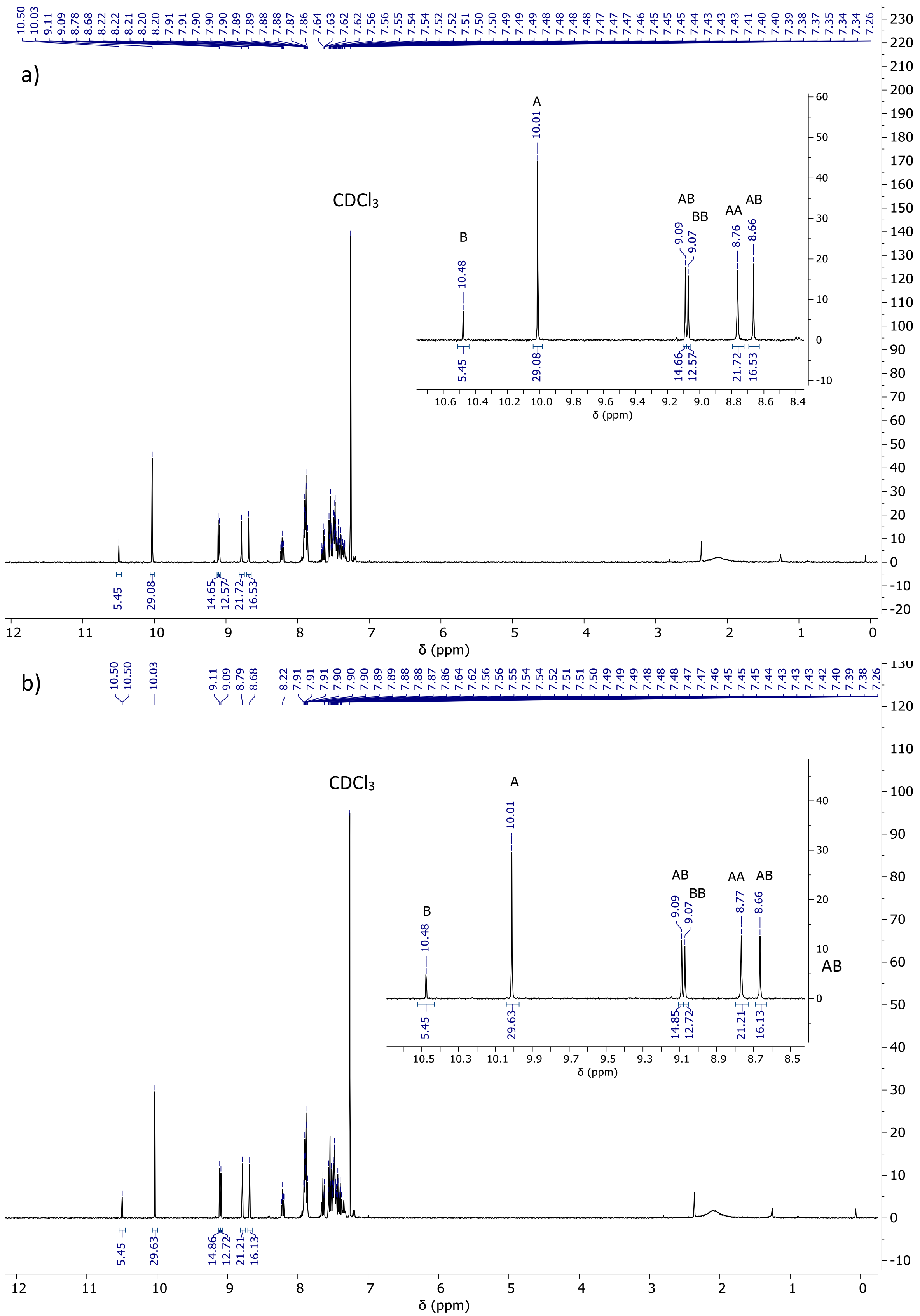

Figure S32: ^{1}H NMR (400 MHz, $CDCl_3$) spectra of equilibrium of **AA** + **B** (14 mM each), in the presence of PTSA (1 eq.), after a) 1 hour and b) 24 hours.

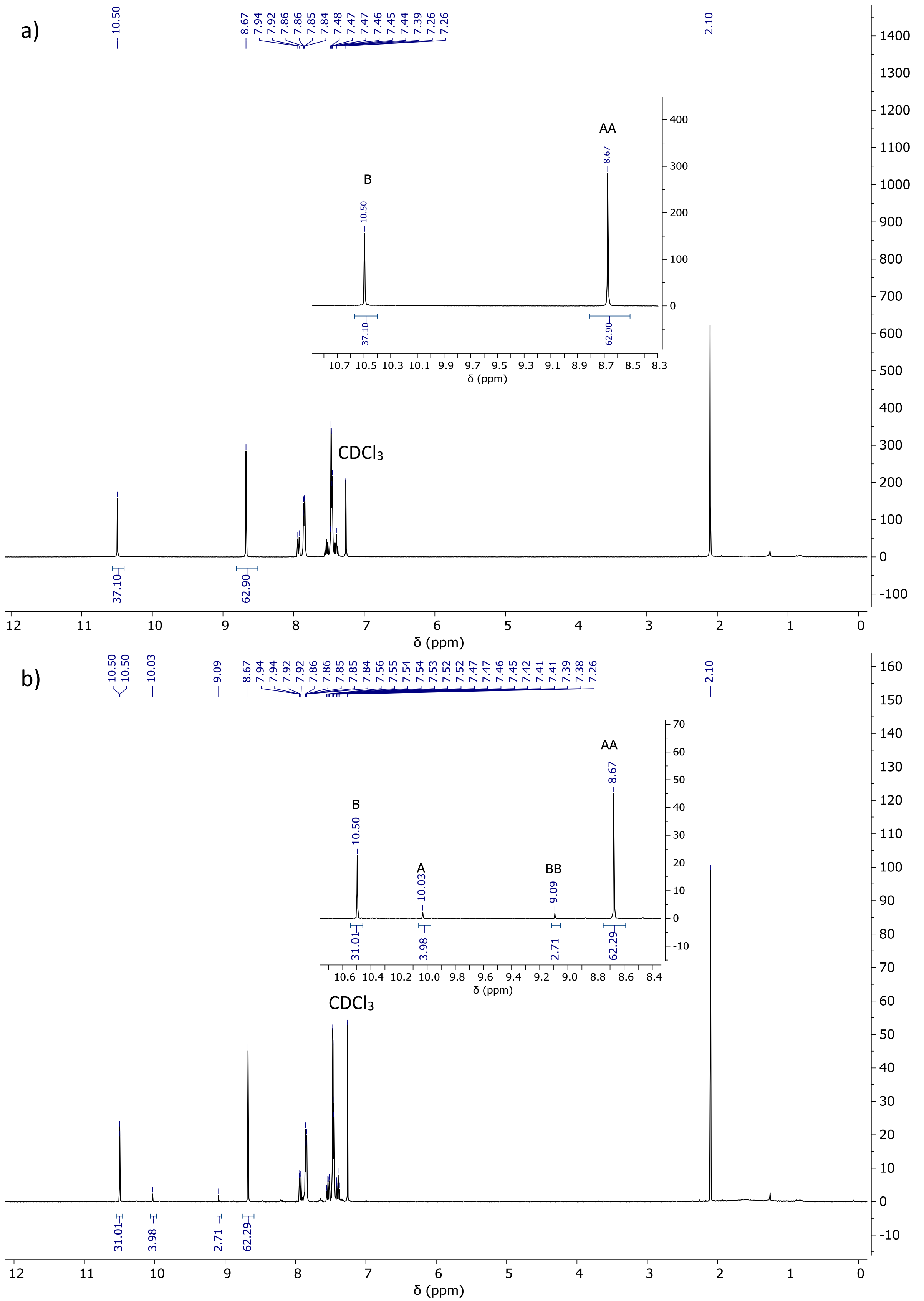

Figure S33: ^{1}H NMR (400 MHz, $CDCl_3$) spectra of equilibrium of **AA** + **B** (14 mM each), in the presence of acetic acid (1 eq.), after a) 24 hours and b) 10 days.

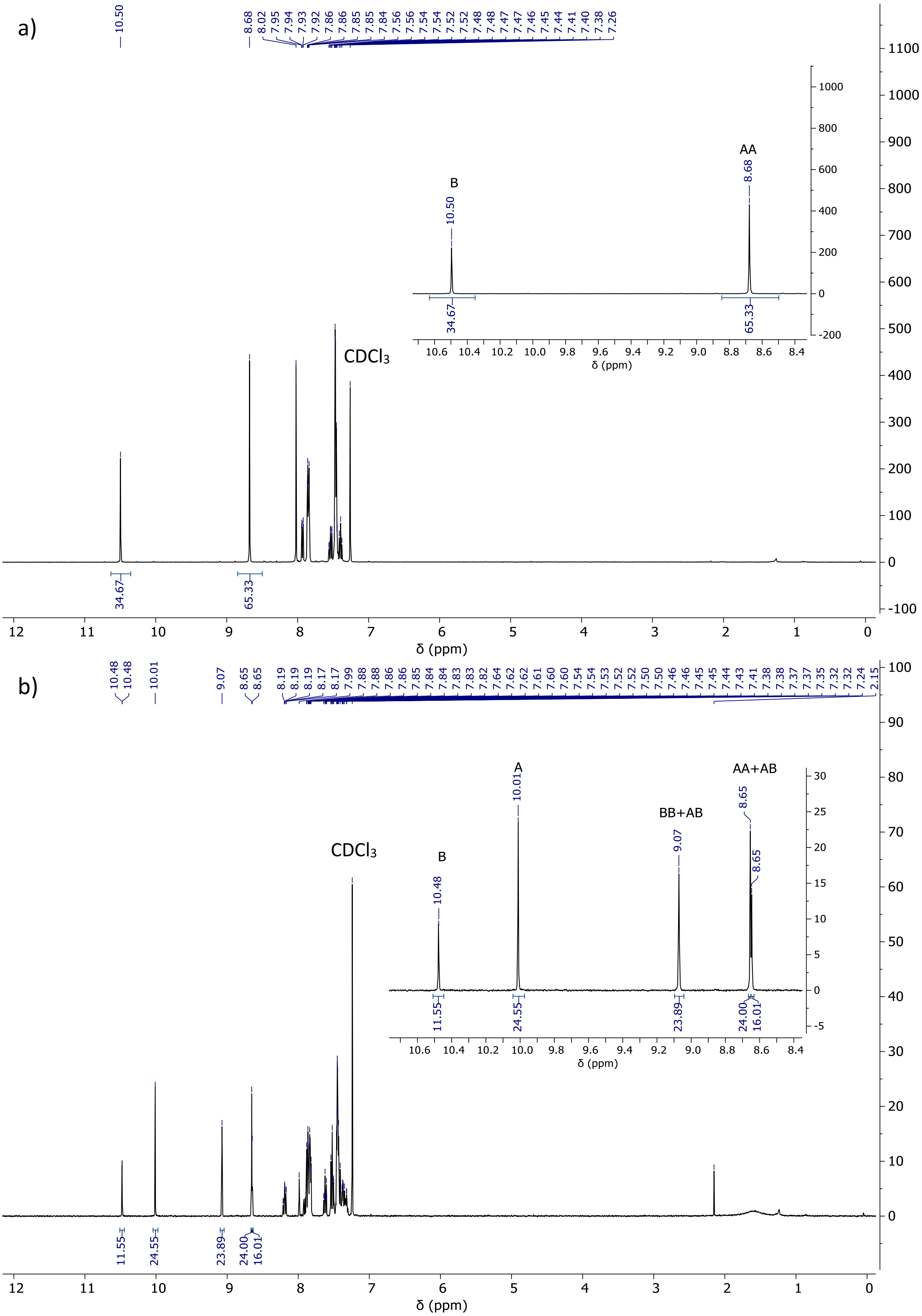

Figure S34: ^{1}H NMR (400 MHz, $CDCl_3$) spectra of equilibrium of **AA** + **B** (14 mM each), in the presence of formic acid (1 eq.), after a) 24 hours and b) 10 days.

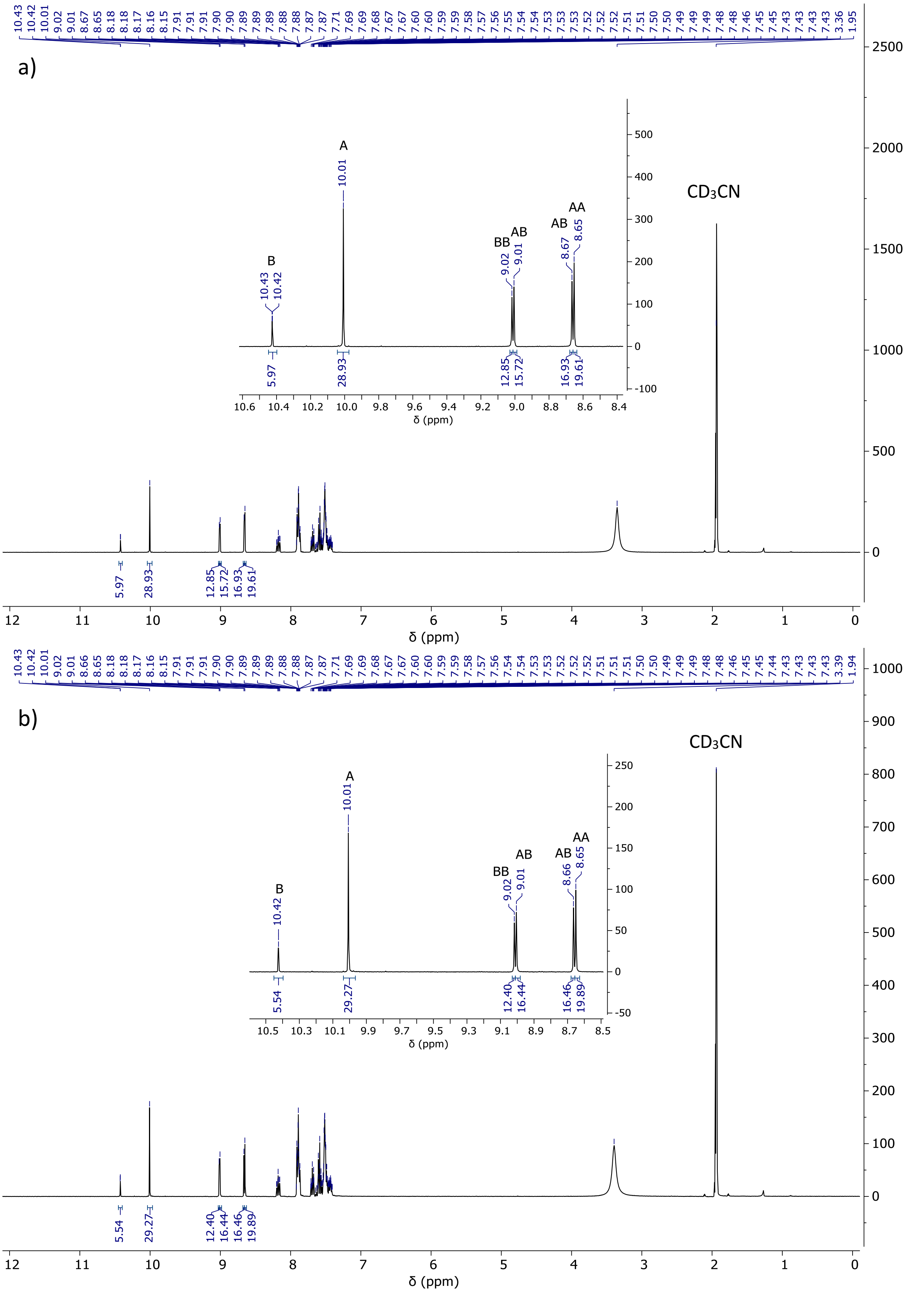

Figure S35: ^{1}H NMR (400 MHz, CD_3CN) spectra of equilibrium of **AA** + **B** (14 mM each), in the presence of TFA (1 eq.), after a) 1 hour and b) 24 hours.

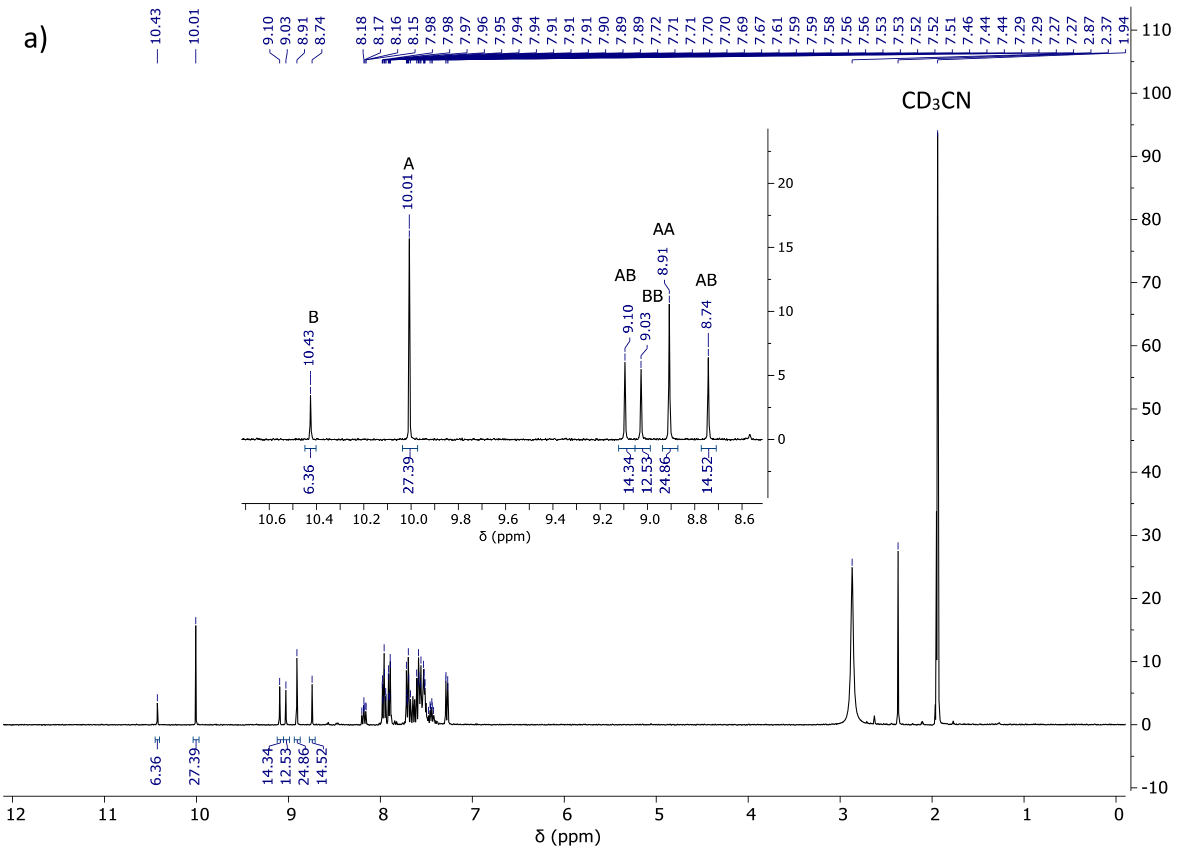

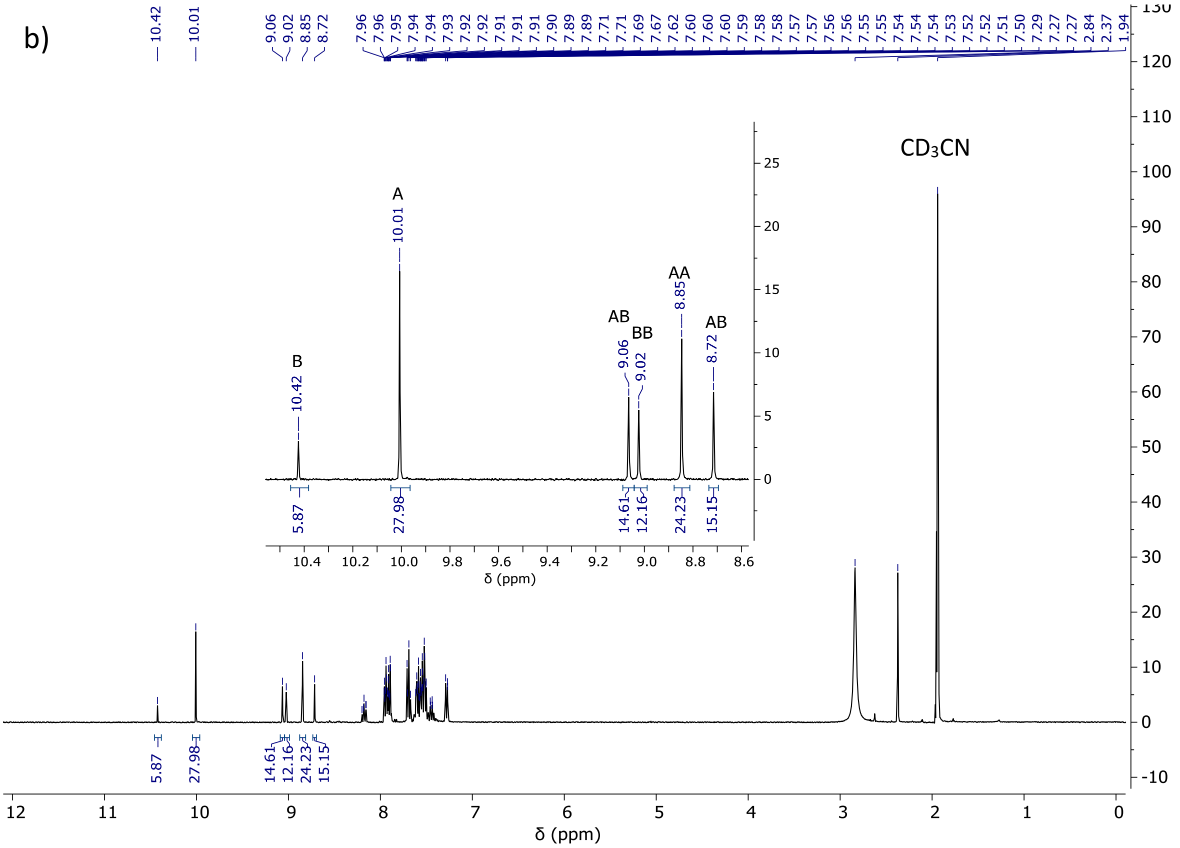

Figure S36: ^{1}H NMR (400 MHz, CD_3CN) spectra of equilibrium of **AA** + **B** (14 mM each), in the presence of PTSA (1 eq.), after a) 1 hour and b) 24 hours.

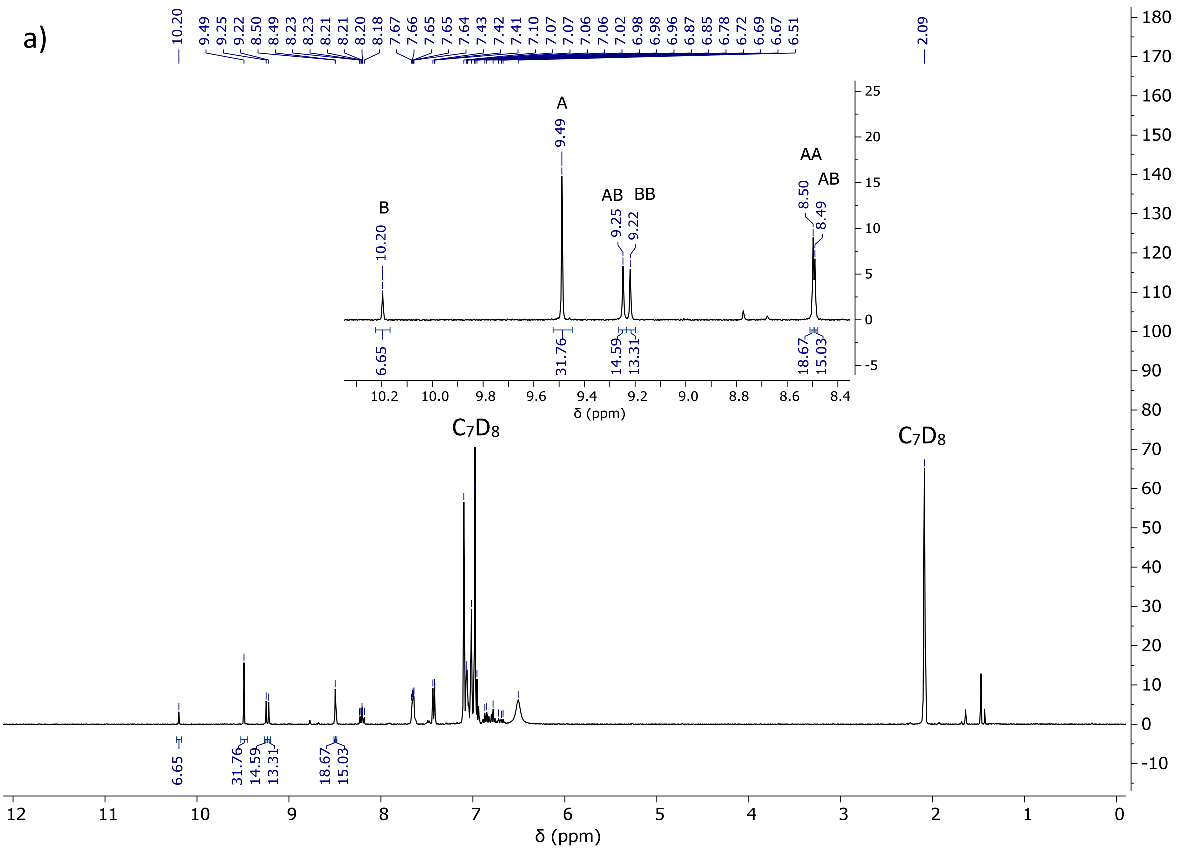

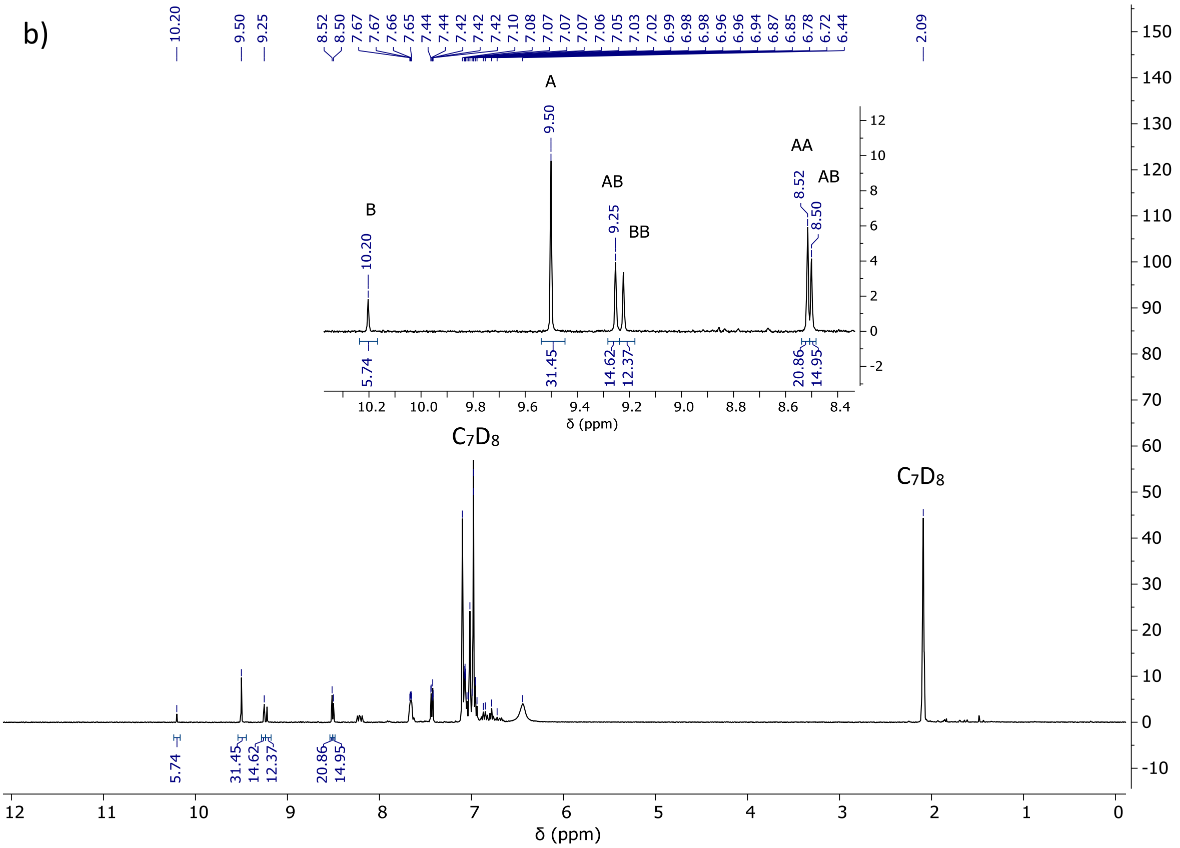

Figure S37: ^{1}H NMR (400 MHz, C_7D_8) spectra of equilibrium of **AA** + **B** (14 mM each), in the presence of TFA (1 eq.), after a) 1 hour and b) 24 hours.

9.2 Effect of water content

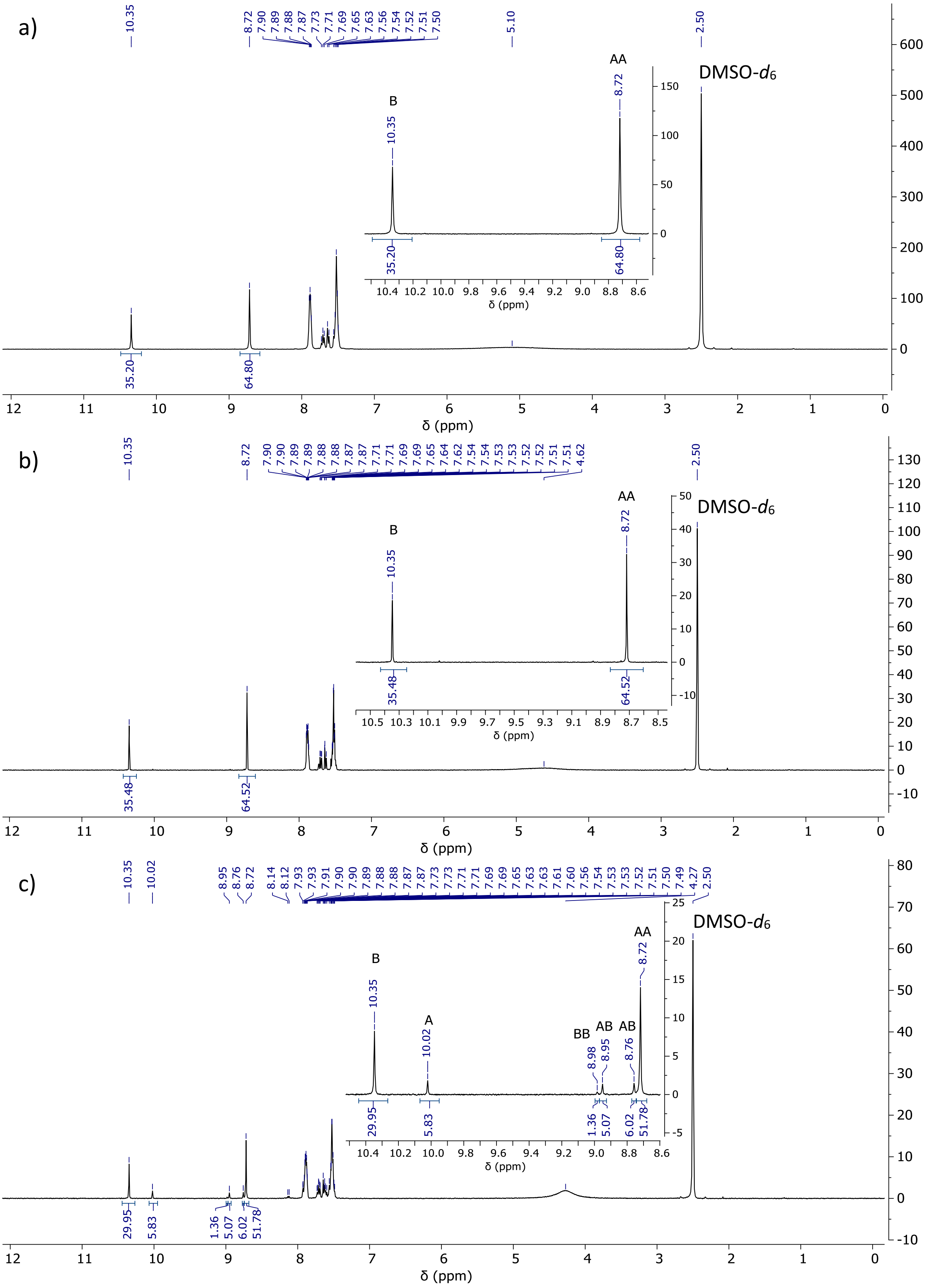

Figure S38: ^{1}H NMR (400 MHz, DMSO-d_6/D_2O, 100:0) spectra of equilibrium of **AA** + **B** (14 mM each), in the presence of TFA (1 eq.), after a) 1 h, b) 24 hours, and c) 7 days.

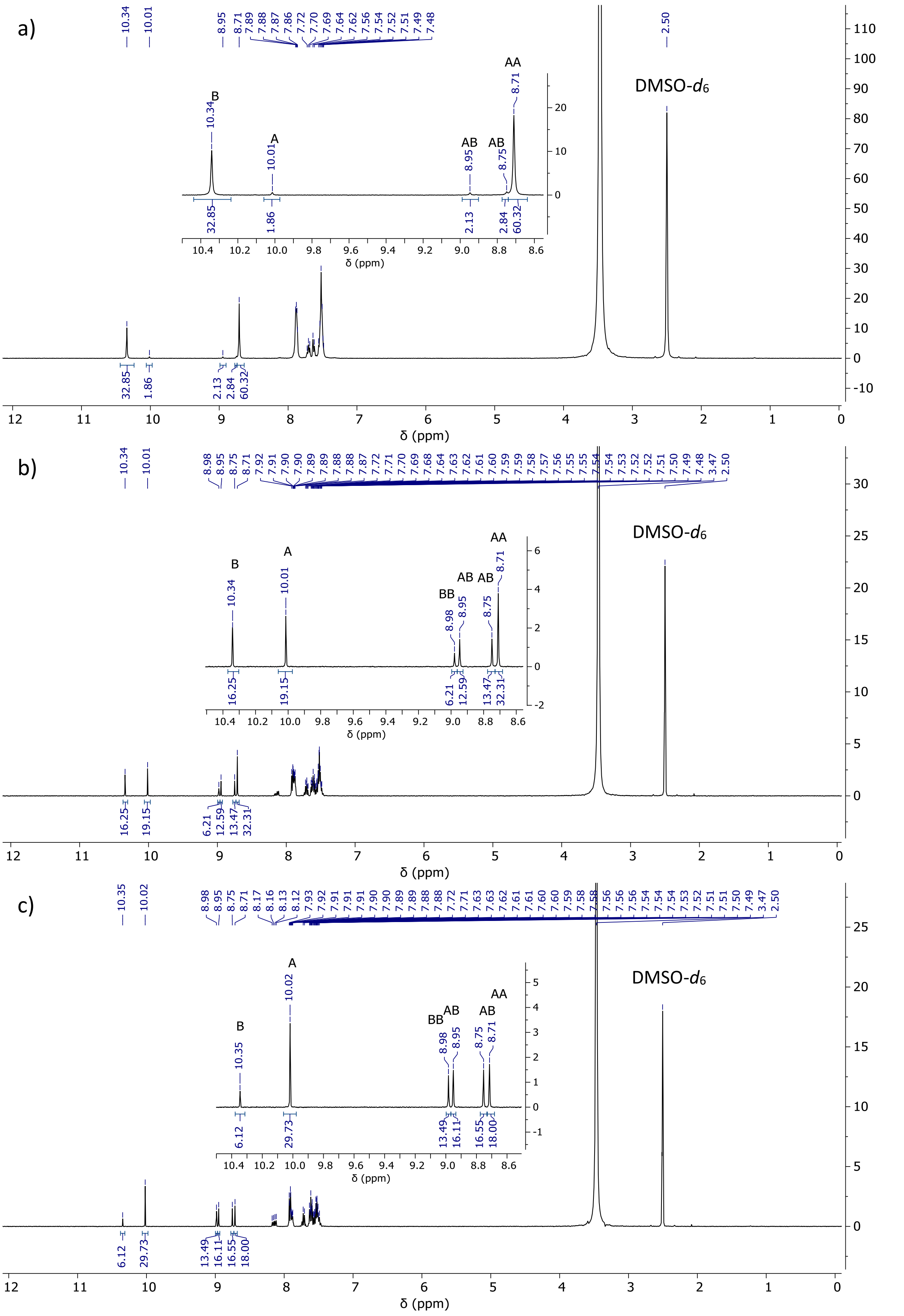

Figure S39: ^{1}H NMR (400 MHz, DMSO-d_6/$D_2$0, 99:1) spectra of equilibrium of **AA** + **B** (14 mM each), in the presence of TFA (1 eq.), after a) 1 h, b) 24 hours, and c) 7 days.

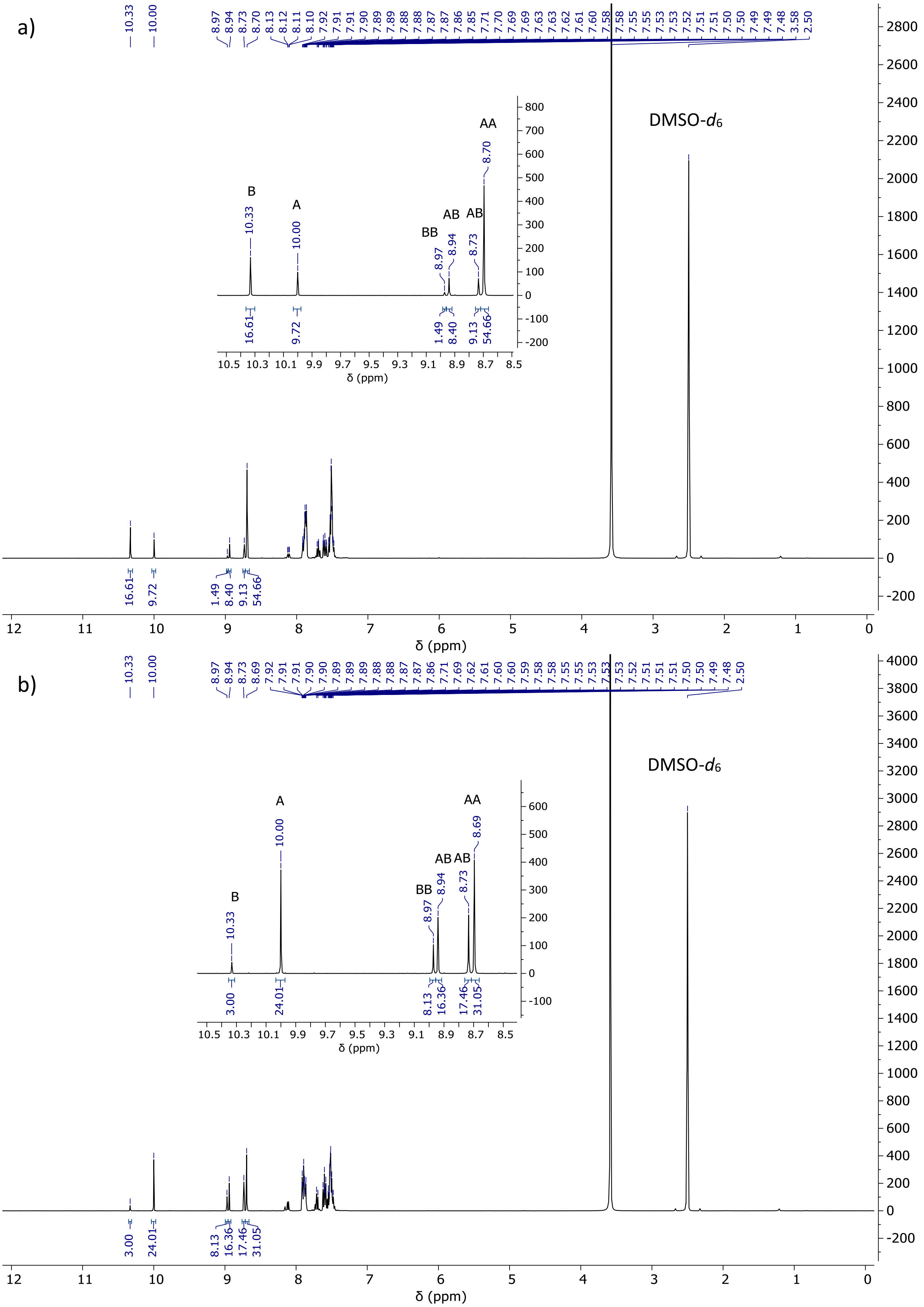

Figure S40: ^{1}H NMR (400 MHz, DMSO-d_6/D_2O, 95:5) spectra of equilibrium of **AA** + **B** (14 mM each), in the presence of TFA (1 eq.), after a) 1 hour and b) 24 hours.

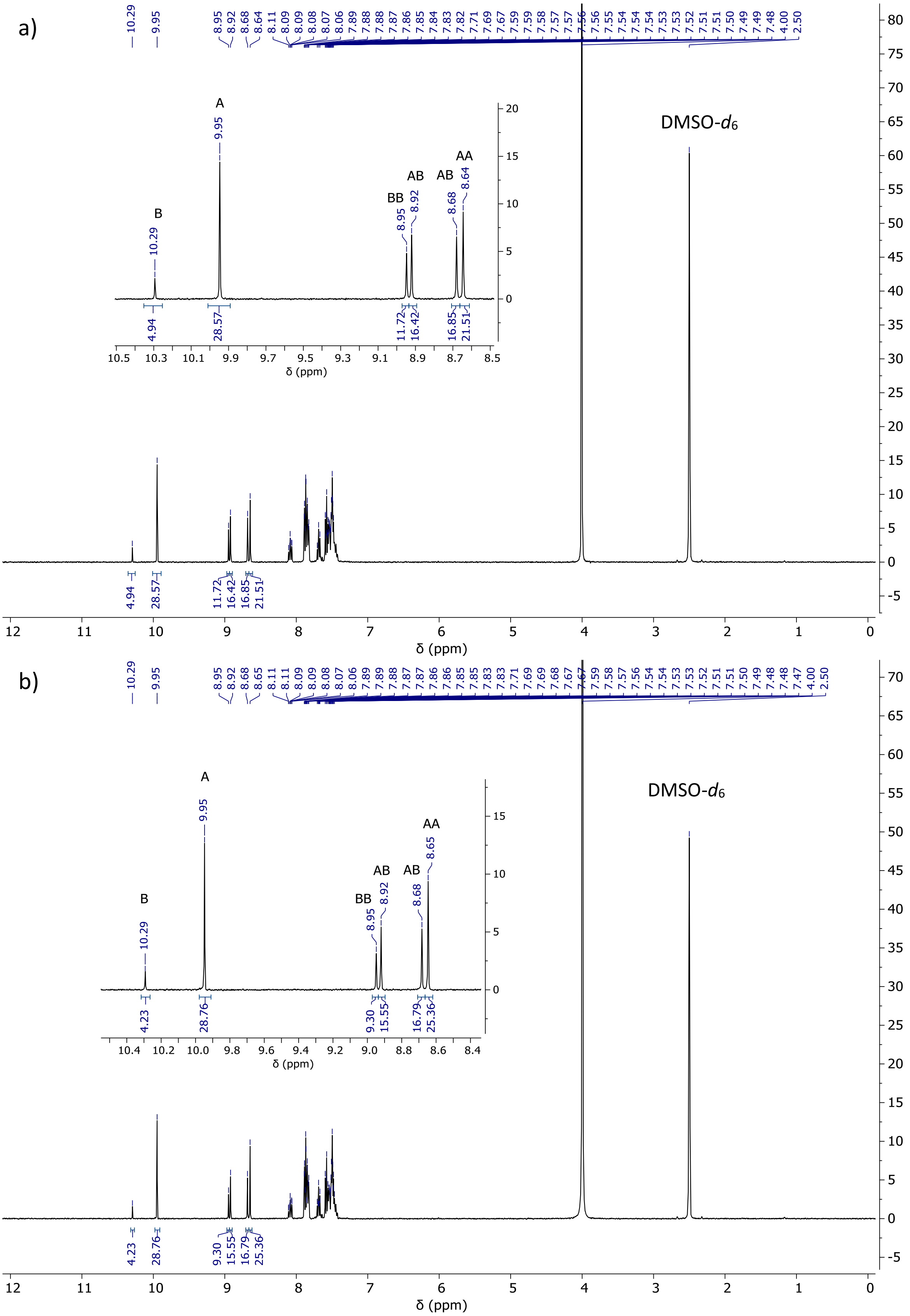

Figure S41: ^{1}H NMR (400 MHz, DMSO-d_6/D_2O, 80:20) spectra of equilibrium of **AA** + **B** (14 mM each), in the presence of TFA (1 eq.), after a) 1 hour and b) 24 hours.

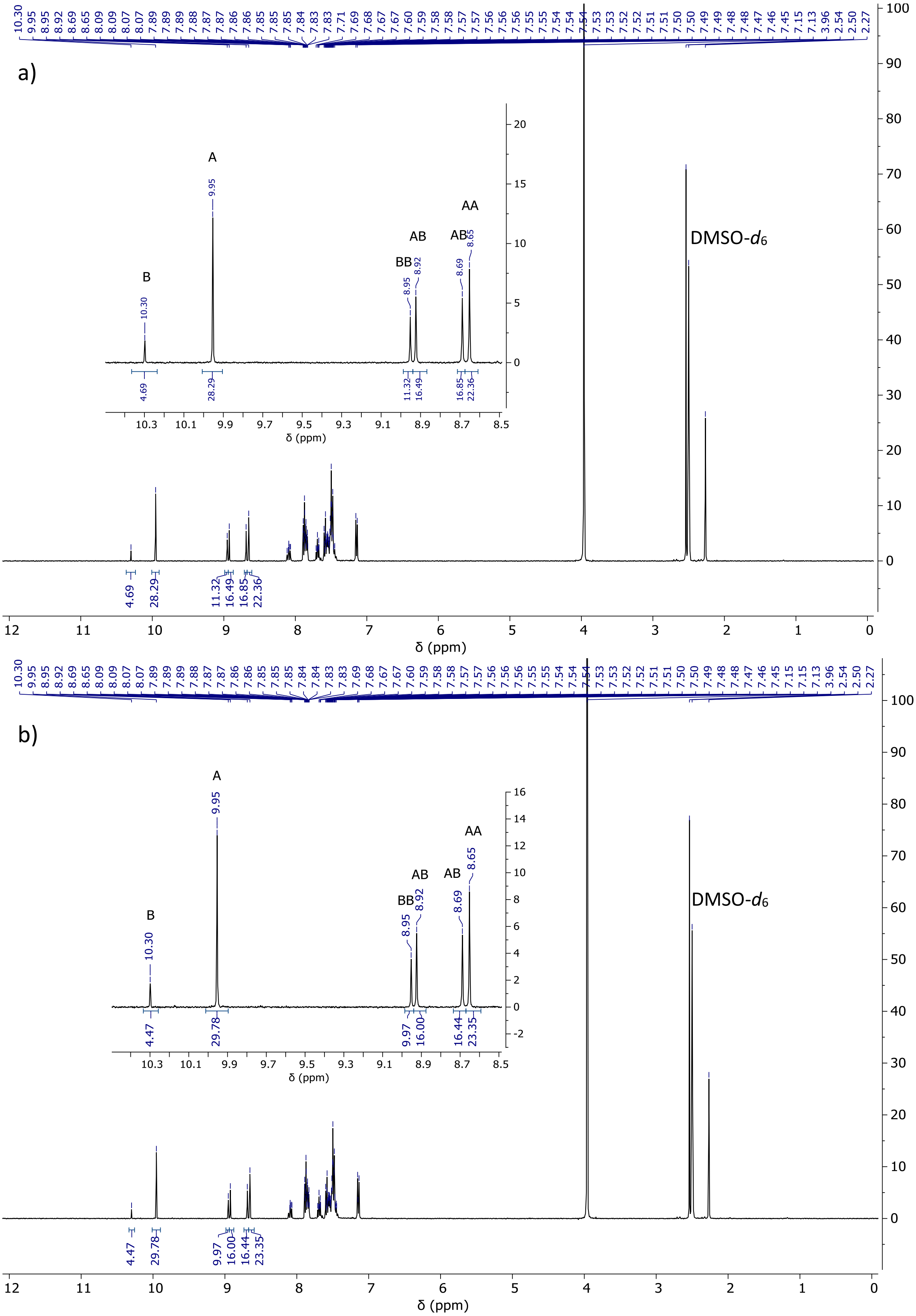

Figure S42: ^{1}H NMR (400 MHz, DMSO-d_6/D_2O, 80:20) spectra of equilibrium of **AA** + **B** (14 mM each), in the presence of PTSA (1 eq.), after a) 1 hour and b) 24 hours.

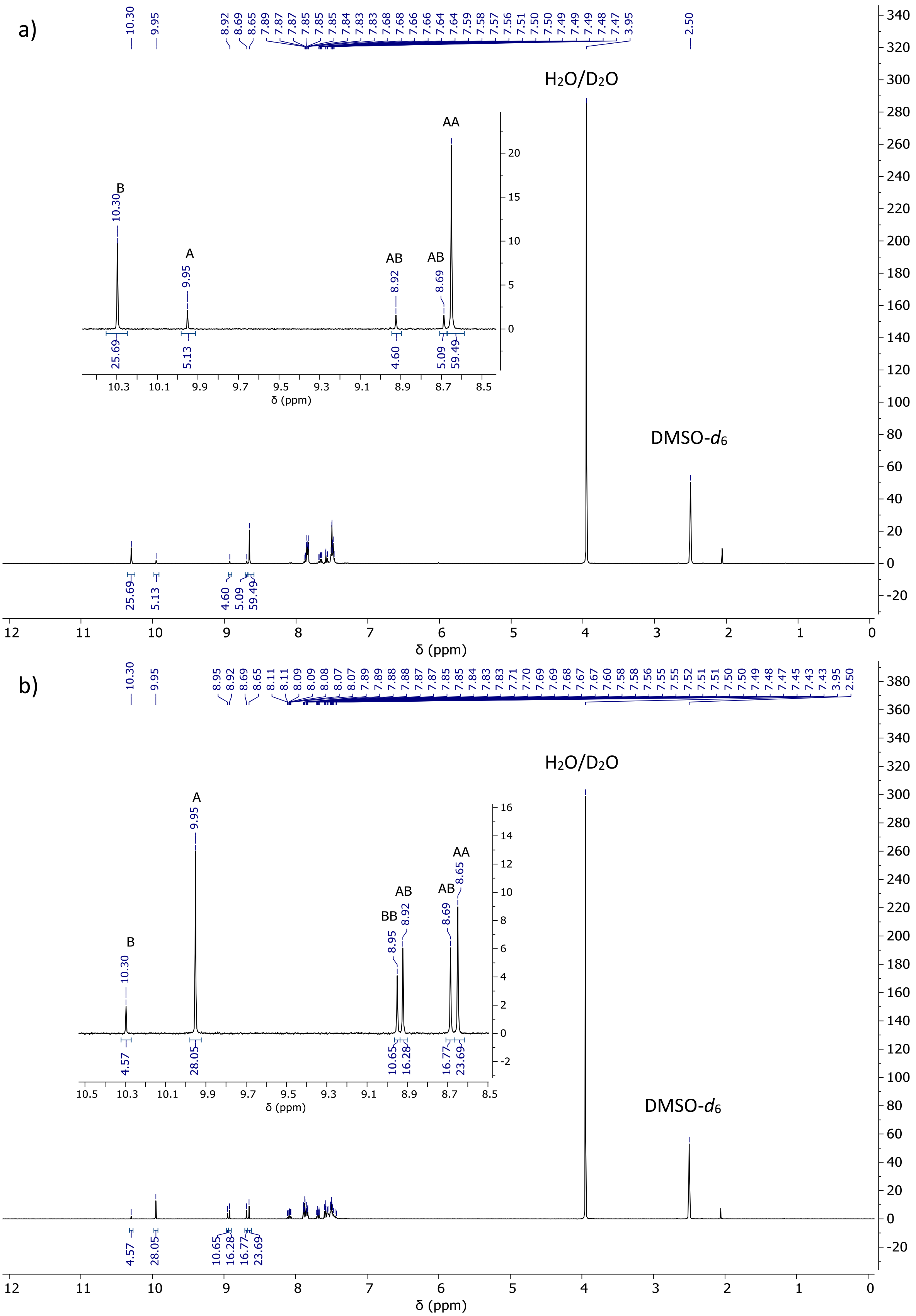

Figure S43: ^{1}H NMR (400 MHz, DMSO-d_6/D_2O, 80:20) spectra of equilibrium of **AA** + **B** (14 mM each), in the presence of TFA (0.1 eq.), after a) 1 hour and b) 24 hours.

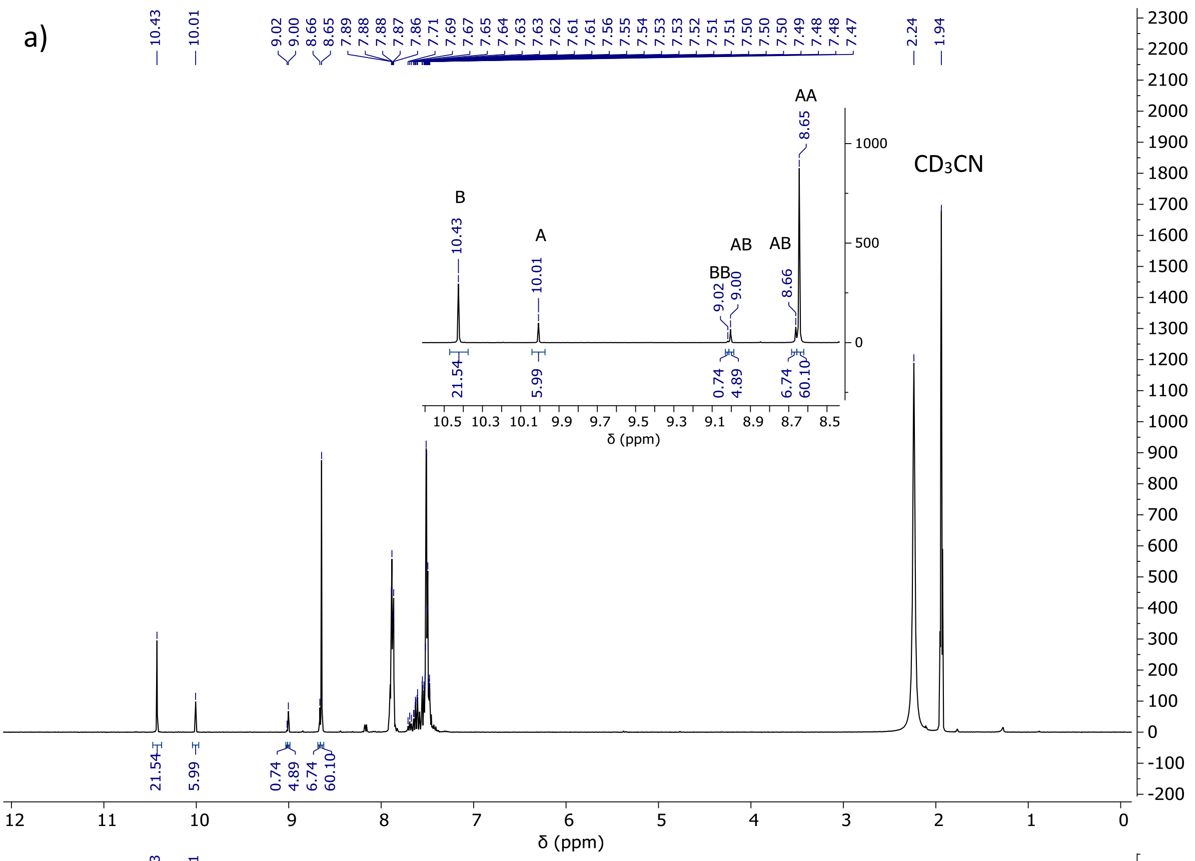

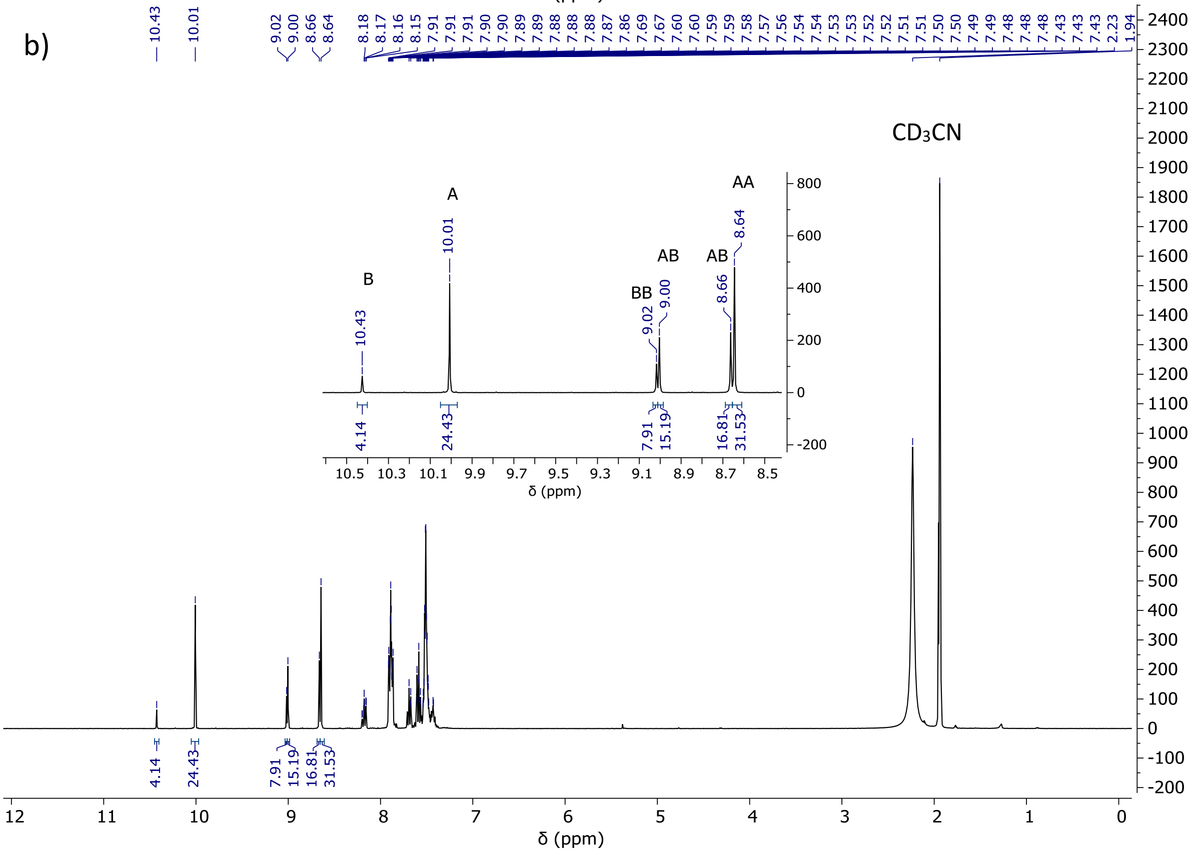

Figure S44: ^{1}H NMR (400 MHz, CD_3CN /D_2O, 100:0) spectra of equilibrium of **AA** + **B** (14 mM each), in the presence of TFA (0.1 eq.), after a) 1 hour and b) 24 hours.

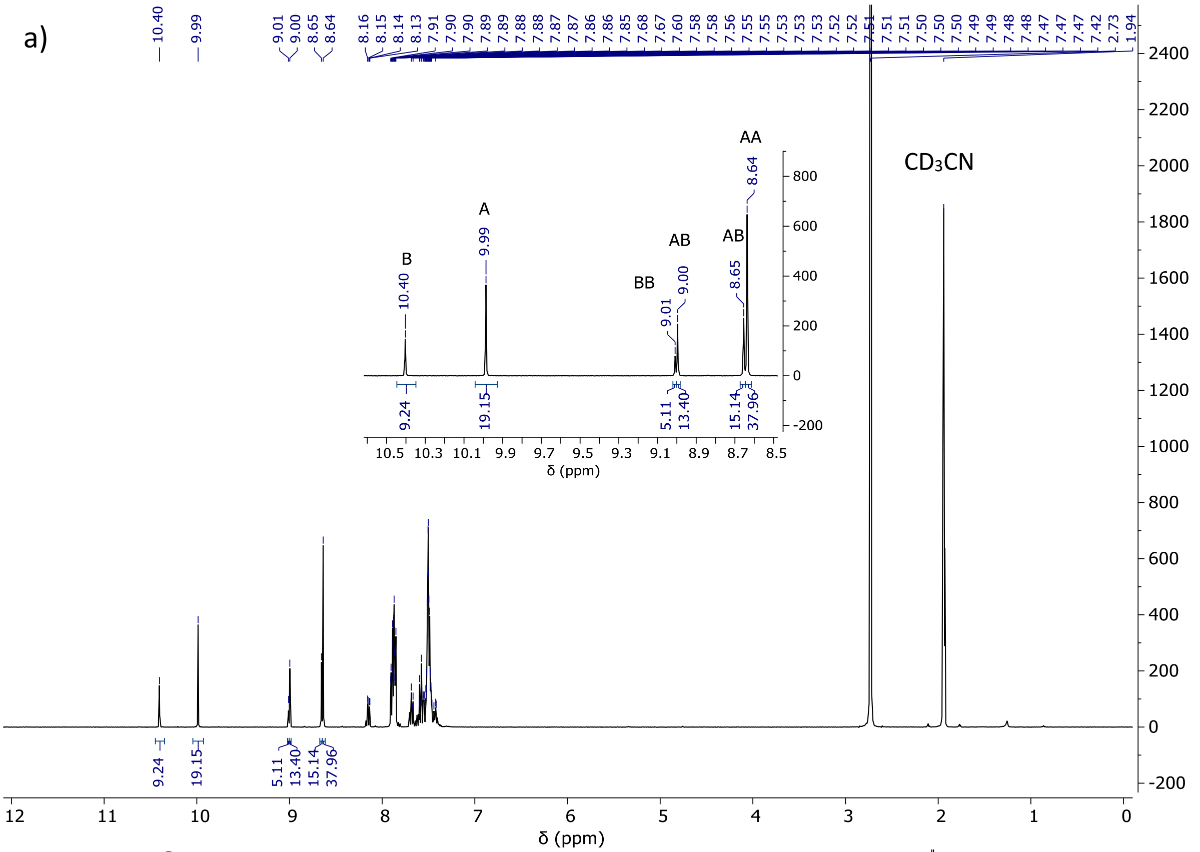

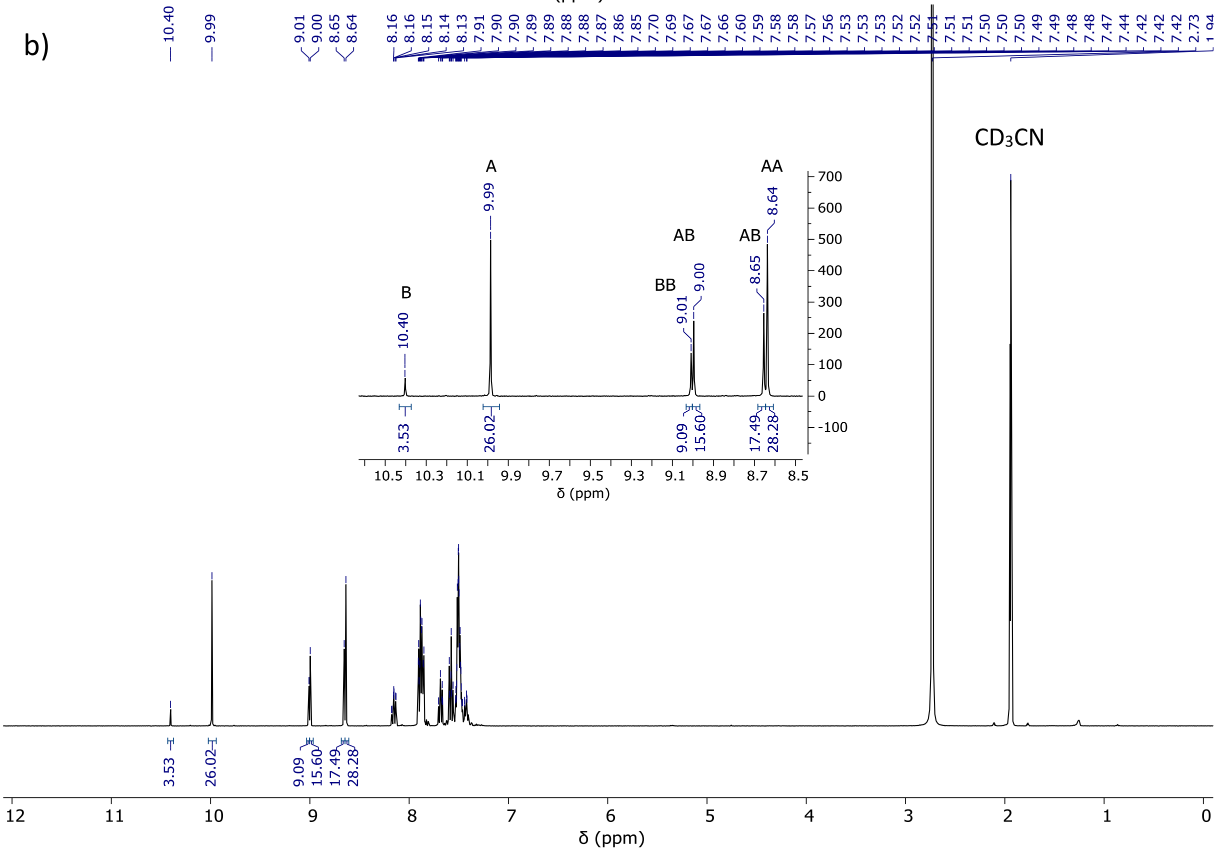

Figure S45: ^{1}H NMR (400 MHz, CD_3CN /D_2O, 95:5) spectra of equilibrium of **AA** + **B** (14 mM each), in the presence of TFA (0.1 eq.), after a) 1 hour and b) 24 hours.

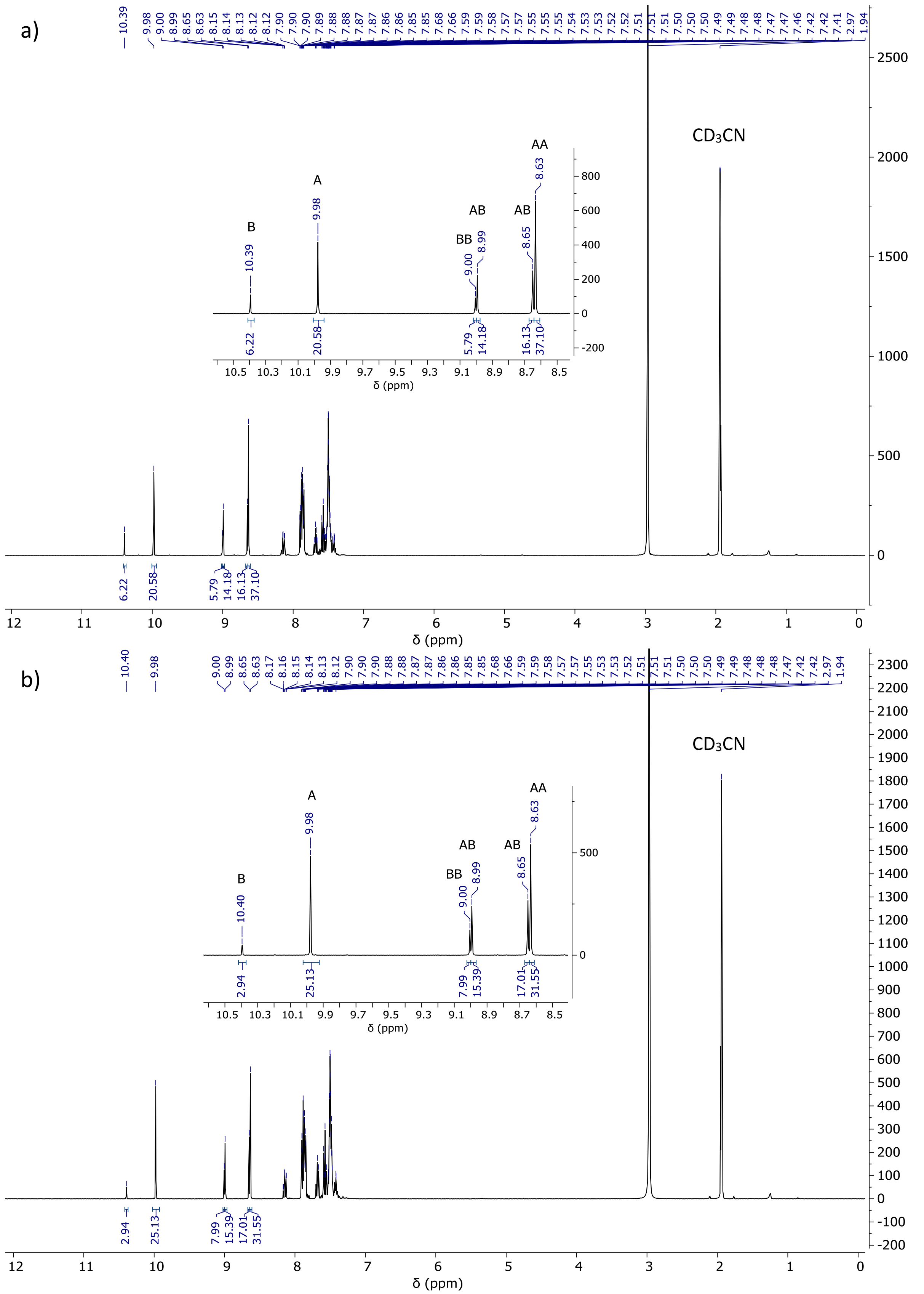

Figure S46: ^{1}H NMR (400 MHz, CD_3CN /D_2O, 90:10) spectra of equilibrium of **AA** + **B** (14 mM each), in the presence of TFA (0.1 eq.), after a) 1 hour and b) 24 hours.

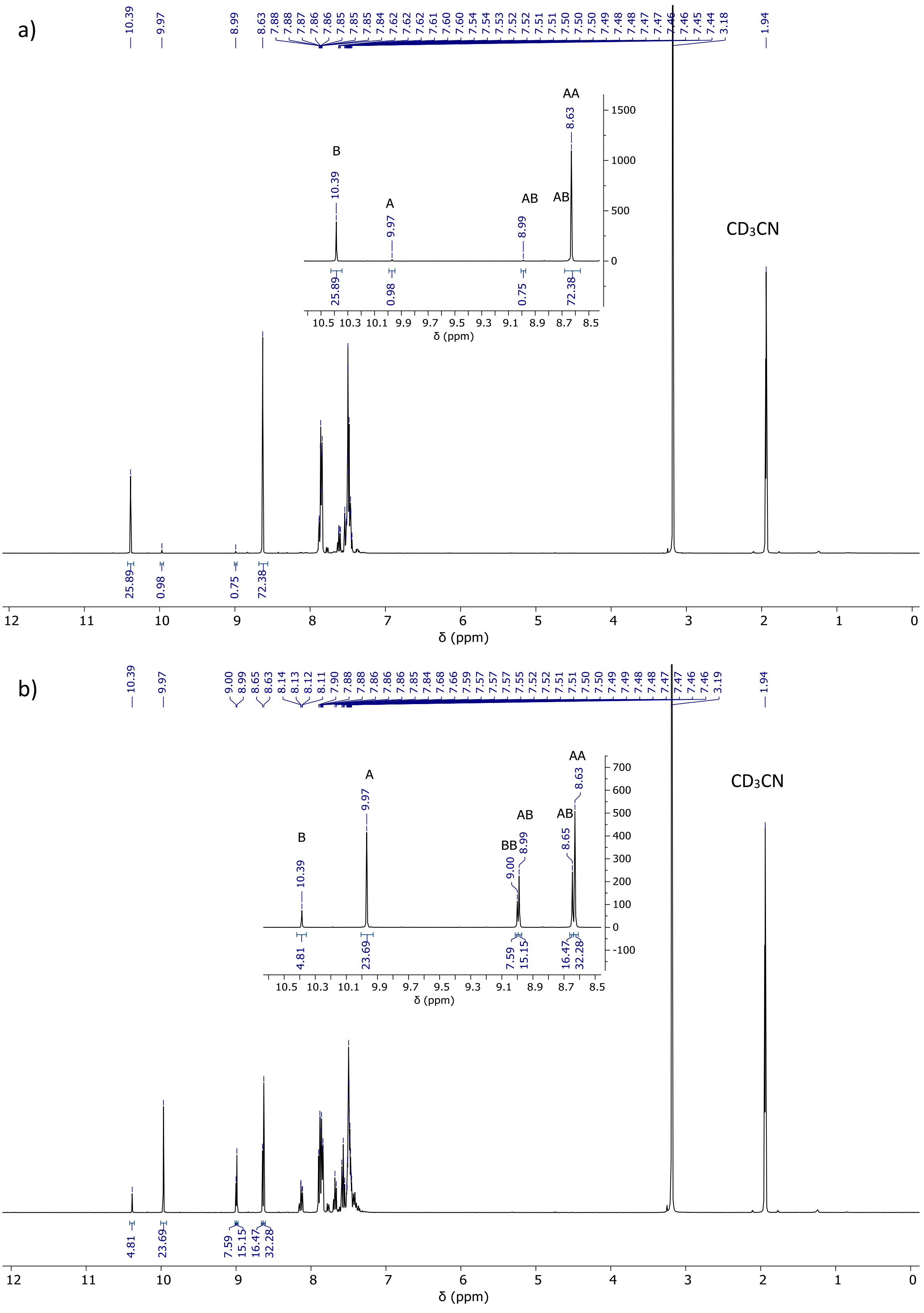

Figure S47: ^{1}H NMR (400 MHz, CD_3CN/D_2O, 90:10) spectra of equilibrium of **AA** + **B** (14 mM each), after a) 1 hour and b) 5 days.

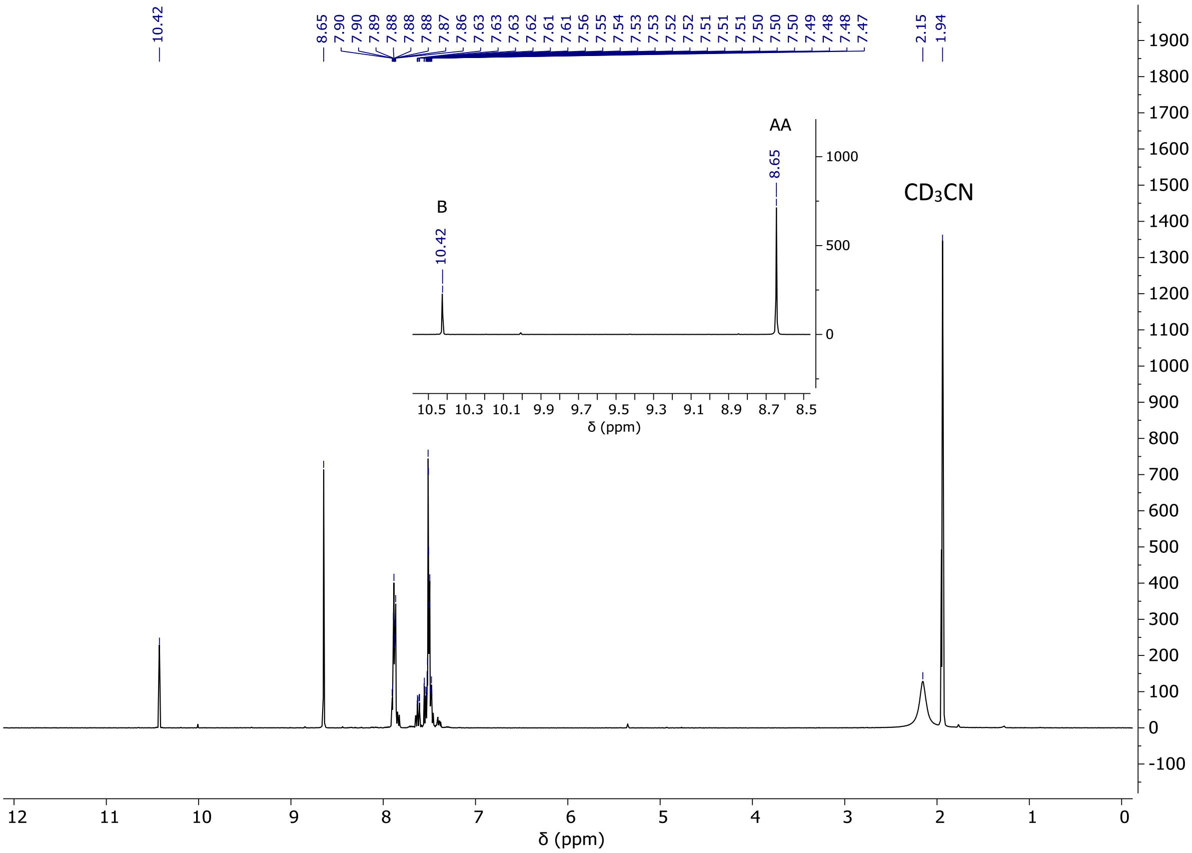

Figure S48: ^{1}H NMR (400 MHz, CD_3CN/D_2O, 100:0) spectra of equilibrium of **AA** + **B** (14 mM each), after 4 days.

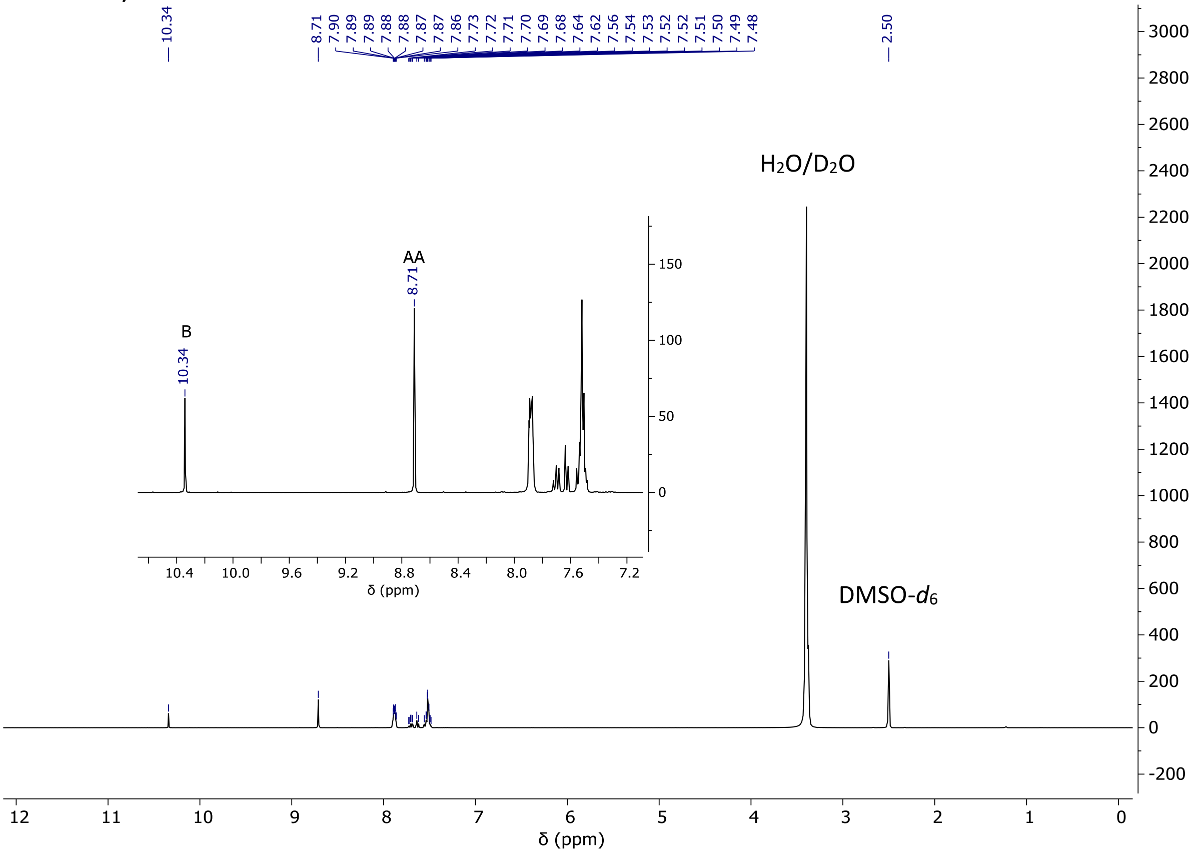

Figure S49: ^{1}H NMR (400 MHz, DMSO-d_6/D_2O, 95:5) spectra of equilibrium of **AA** + **B** (14 mM each) without acid after 10 days.

9.3 Low concentration DCLs with water as co-solvent

The azine exchange was tested at lower concentrations (0.4 mM) in a co-solvent mixture DMSO-d_6/H_2O (80:20). For these reactions azine (0.4 mM, 1 eq.), aldehyde (0.4 mM, 1 eq.), and selected catalysts (0.4 mM, 1 eq.) were mixed in DMSO-d_6/H_2O (80:20) (V_{total}= 500 µL). The reactions were performed in glass scintillation vials (2 mL). After the addition of all reagents, the contents of the vials were mixed. The progress of the reaction was monitored with ^{1}H NMR spectroscopy after 1 h, 24 h, and 7 days.

Table S4: Azine exchange reaction at lower concentration (0.4 mM) in co-solvent mixture (DMSO-d_6/H_2O, 80:20). h = hour; d = days.

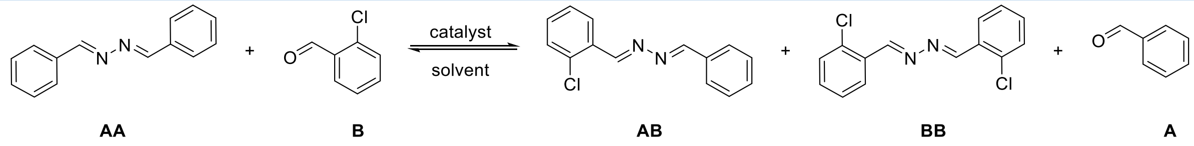

Entry	Catalyst	Reaction time	Molar ratio (%)				
			A	B	AA	BB	AB
		1 h	9	42	46	0	3
1	TFA	24 h	57	10	10	6	17
		7 d	63	13	6	5	12

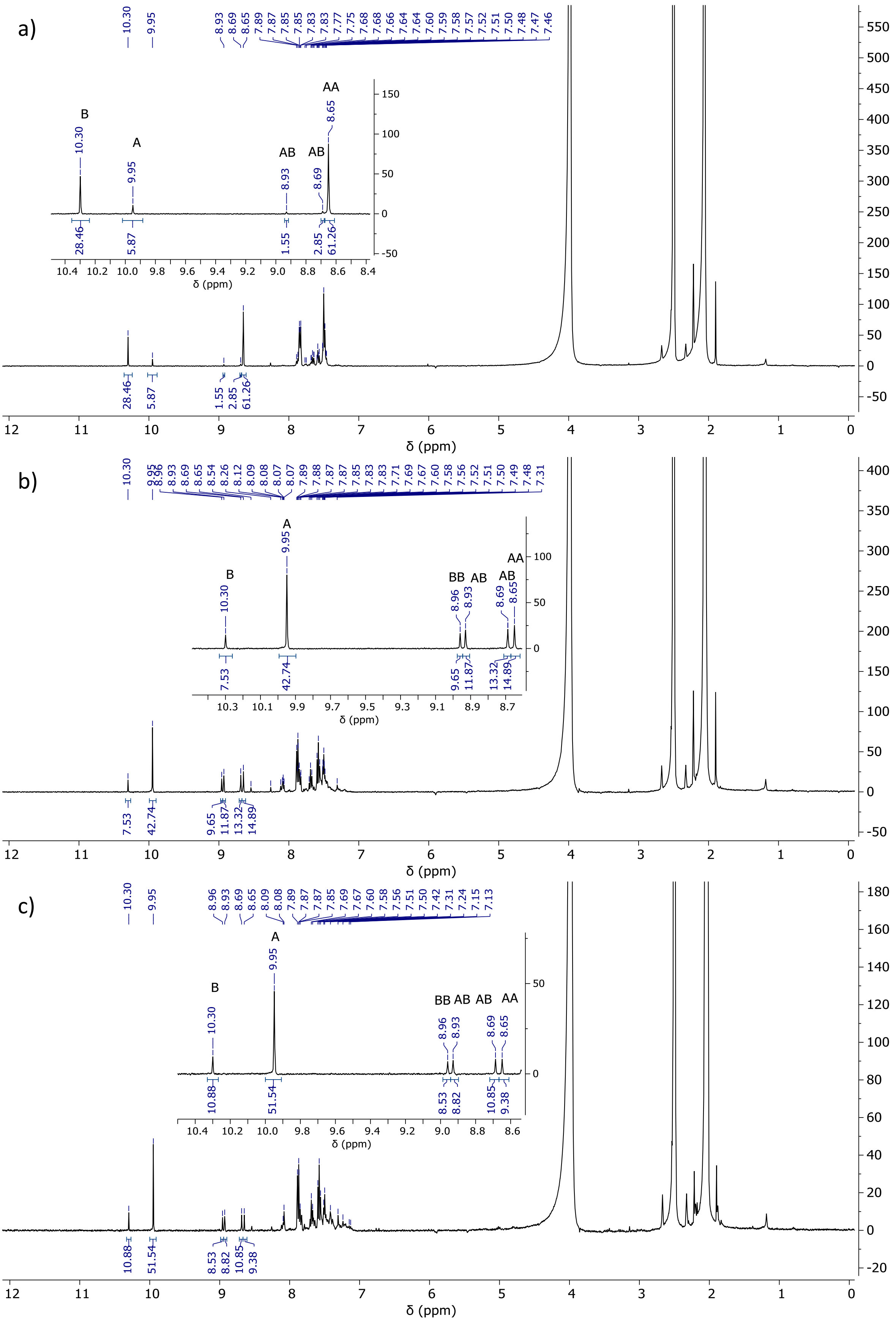

Figure S50: ^{1}H NMR (400 MHz, DMSO-d_6/D_2O, 80:20) of azine **AA** (0.4 mM) and **B** (0.4 mM) exchange reaction, in the presence of TFA (1 eq.), after a) 1 hour, b) 1 day, and c) 7 days.

10. The role of water in the mechanism

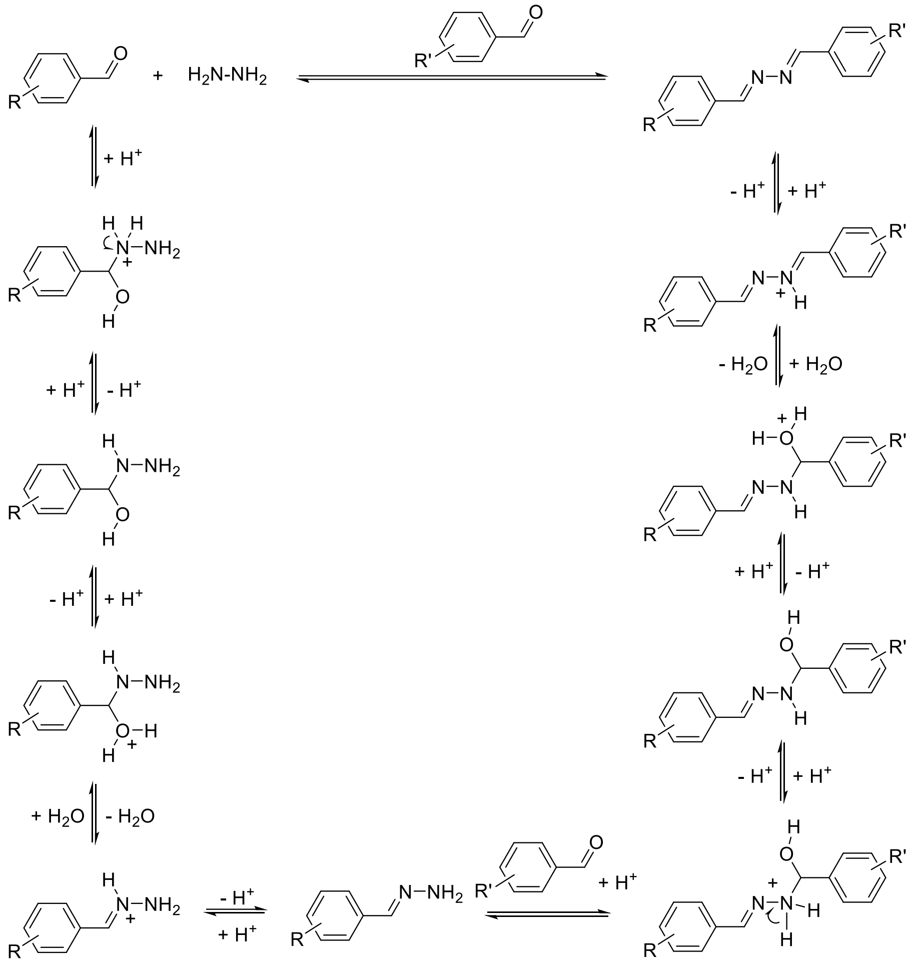

Figure S51: Proposed mechanism for the acid-catalysed formation and hydrolysis of azines.

11. DCLs freezing

The possibility of locking or freezing the azine exchange reaction was explored using 2 equivalents of K_2CO_3 (28 mM) after the reaction started. For each sample, identical amounts of azine **AA** and aldehyde **B** (14 mM) and 1 equivalent of TFA in DMSO-*d*$_6$/D_2O (90:10) were mixed in 2 mL glass scintillation vials. The base was added after 3 min, 20 min, and 40 min, respectively. In addition, one sample without base was studied for comparison. All the samples were examined by ^{1}H NMR spectroscopy five hours after adding the acid.

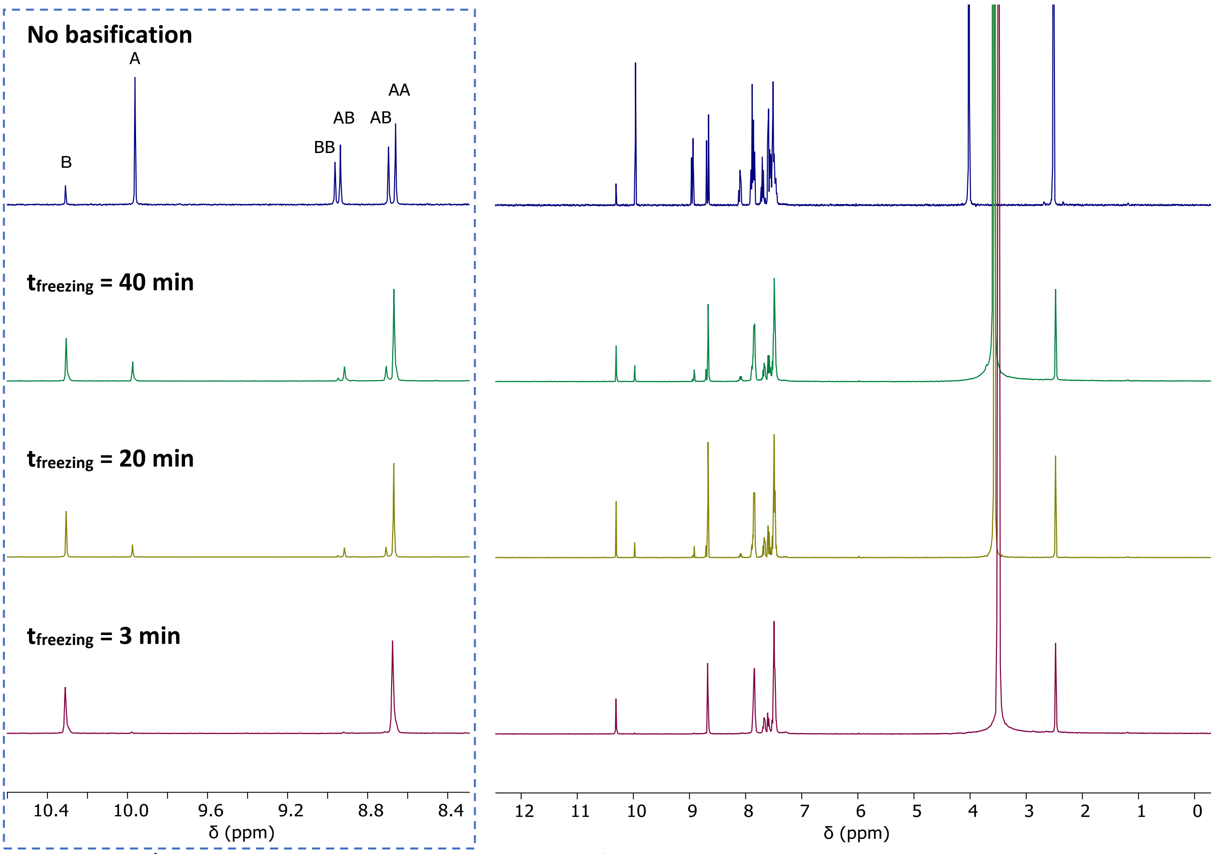

Figure S52: ^{1}H NMR spectra (400 MHz, DMSO-*d*$_6$/D_2O, 90:10) of azine **AA** (14 mM), **B** (14 mM), and TFA (14 mM) exchange reaction. After the reaction started, K_2CO_3 (28 mM, 2 eq.) was added at 3 min, 20 min, and 40 min. For comparison, the spectrum without K_2CO_3 is presented. Inset: expansion on the ^{1}H NMR spectra ranges from 8.4 ppm to 10.4 ppm.

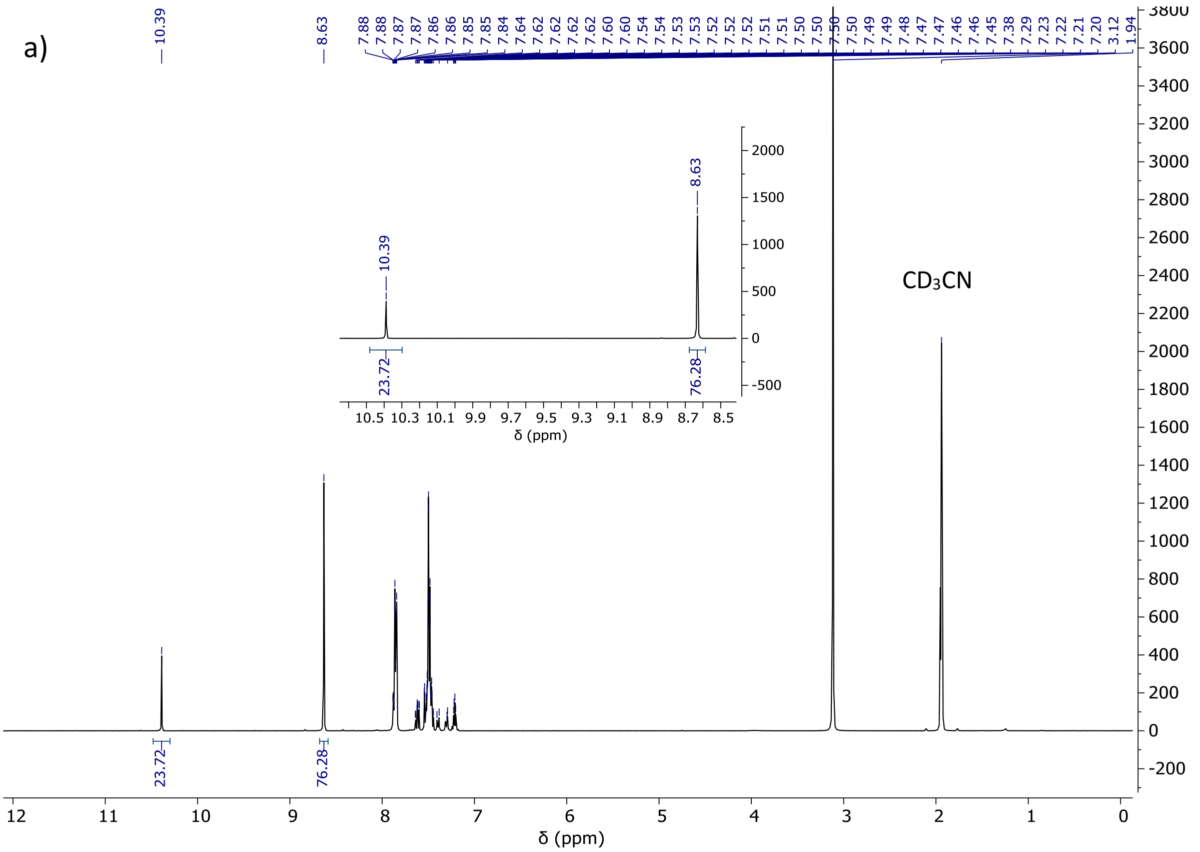

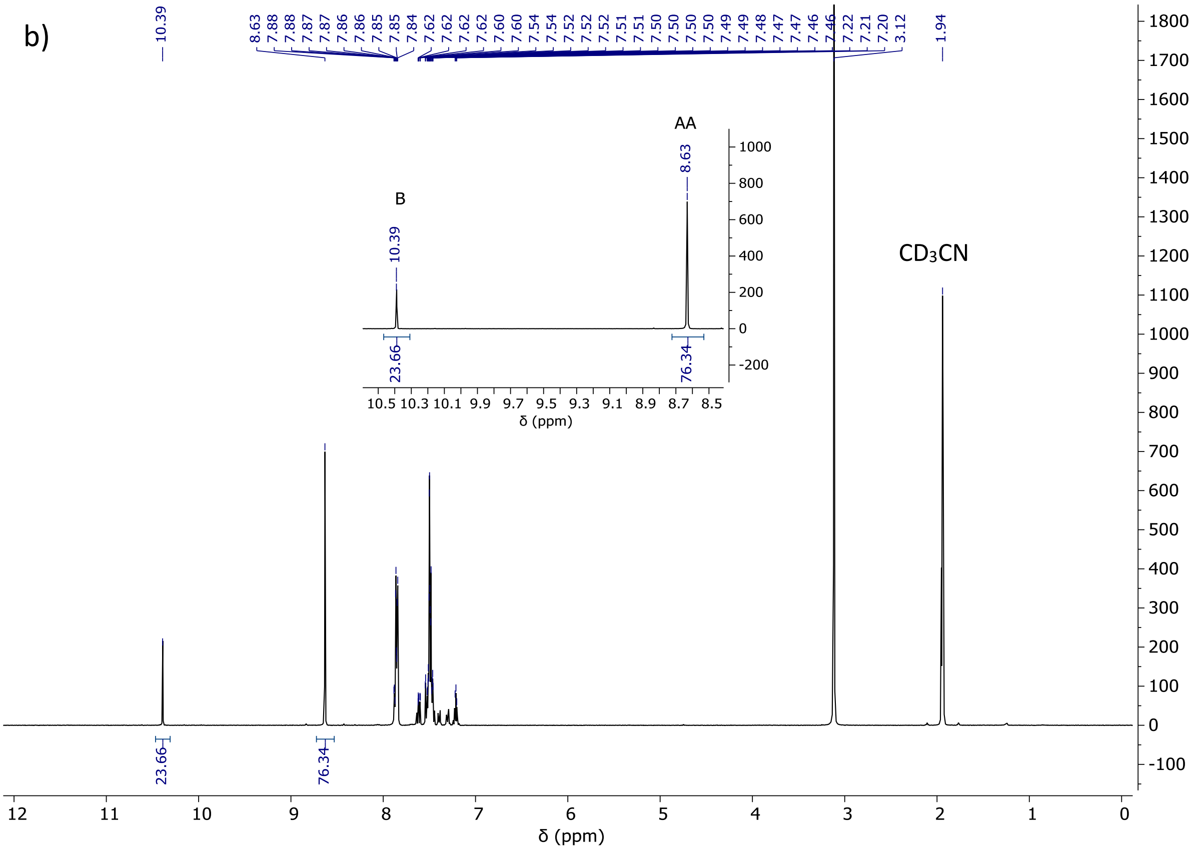

Figure S53: ^{1}H NMR (400 MHz, CD_3CN/D_2O, 90:10) spectra of equilibrium of **AA** + **B** (14 mM each), in the presence of K_2CO_3 (1 eq.), after a) 1 hour and b) 48 hours.

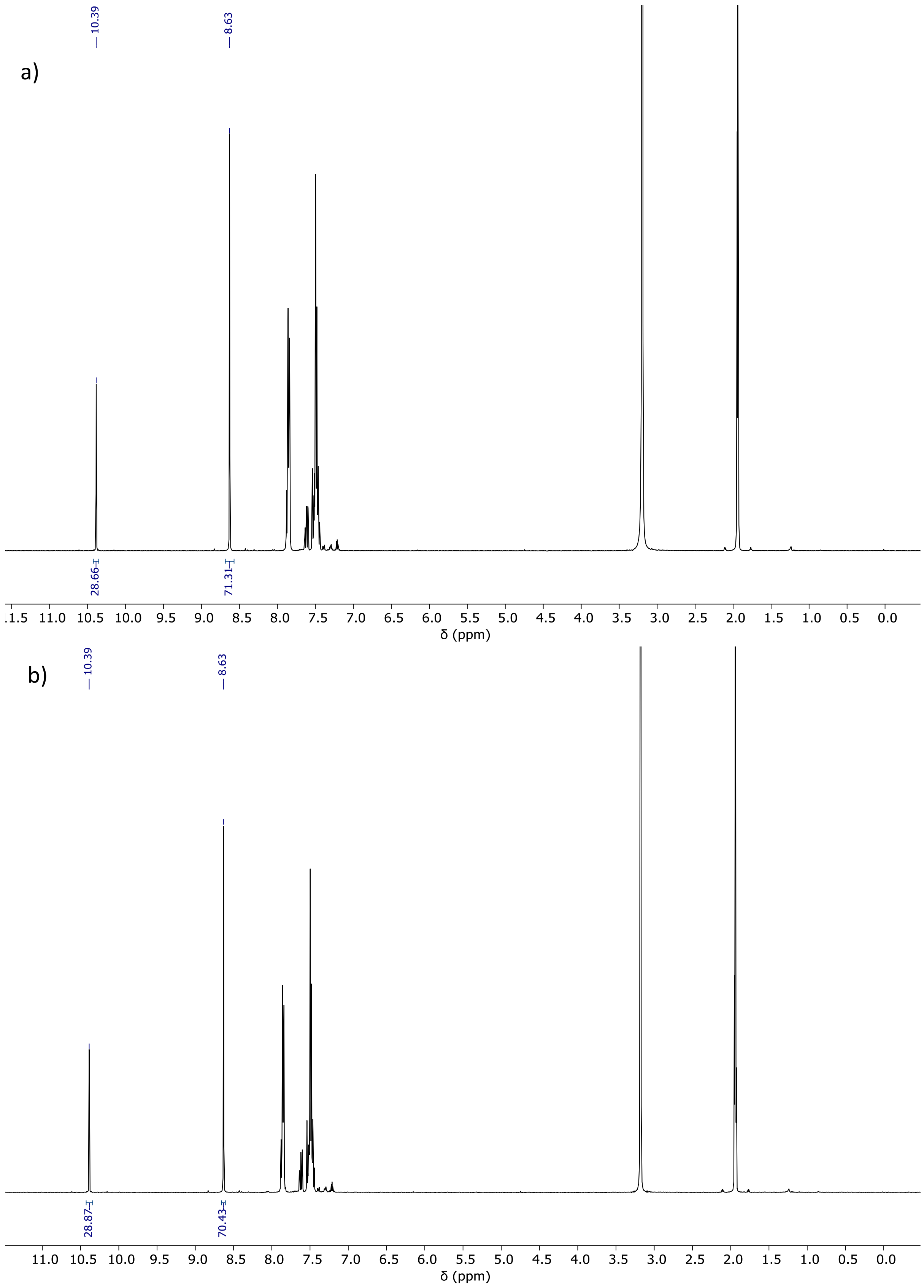

Figure S54: ^{1}H NMR (400 MHz, CD_3CN/D_2O, 90:10) spectra of equilibrium of **AA** + **B** (14 mM each), in the presence of K_2CO_3 (0.1 eq.), after a) 1 day and b) 21 days.

12. The effect of electron donating and withdrawing substituents on exchange

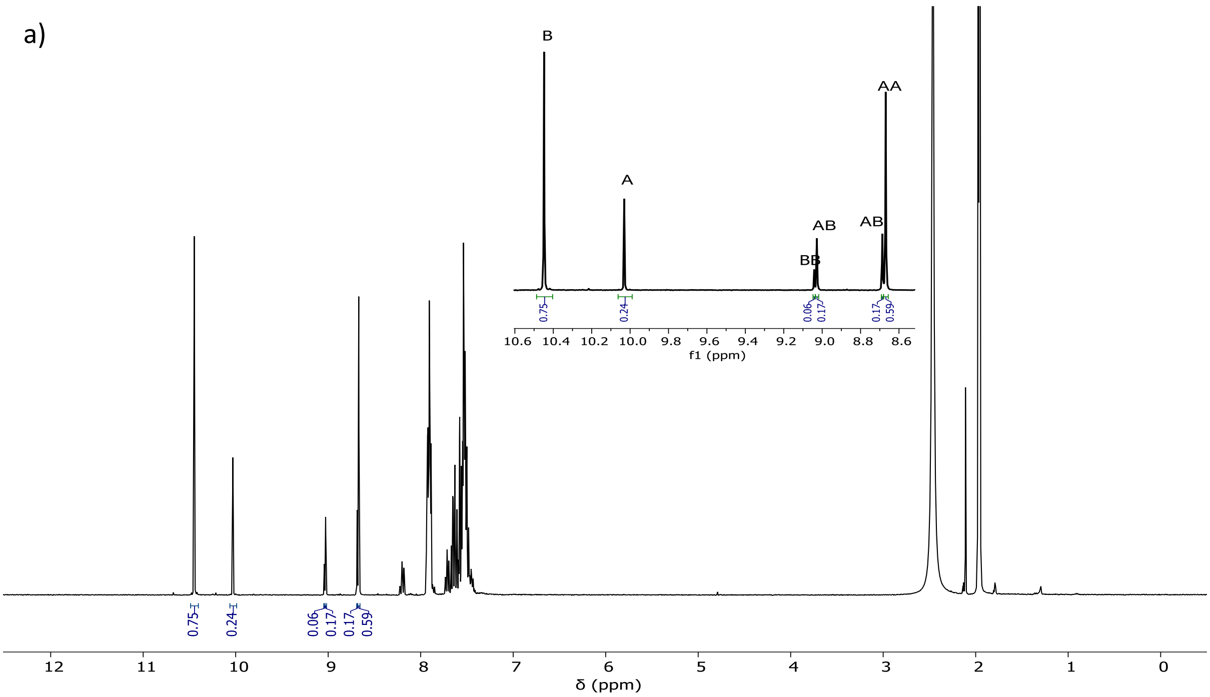

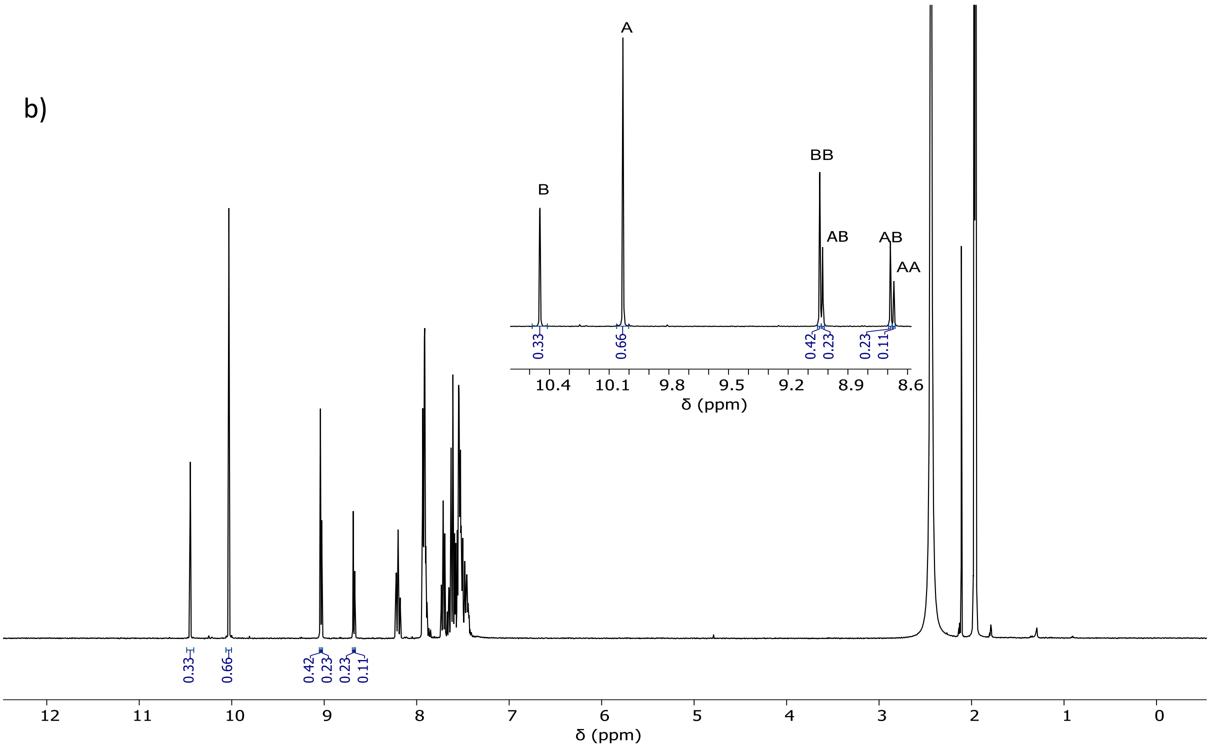

Figure S55: ^{1}H NMR (400 MHz, CD_3CN) spectra of **AA** + **B** equilibrium (7 mM **AA**, 14 mM **B**), in the presence of TFA (1 eq.), after a) 1 hour and b) 24 hours. No further change was observed upon longer equilibration times.

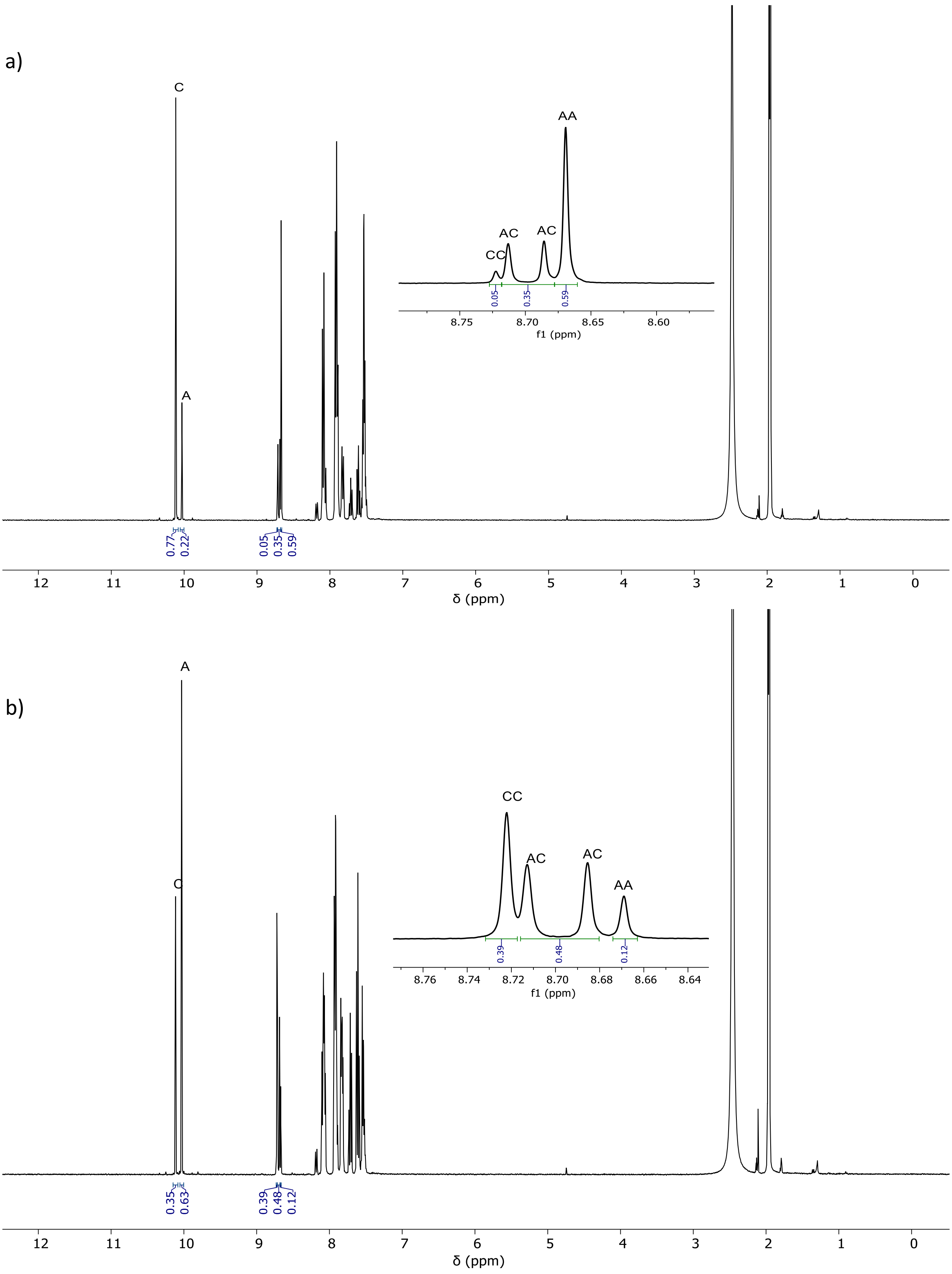

Figure S56: ^{1}H NMR (400 MHz, CD_3CN) spectra of **AA** + **C** equilibrium (7 mM **AA**, 14 mM **C**), in the presence of TFA (1 eq.), after a) 1 hour and b) 24 hours. No further change was observed upon longer equilibration times.

a)

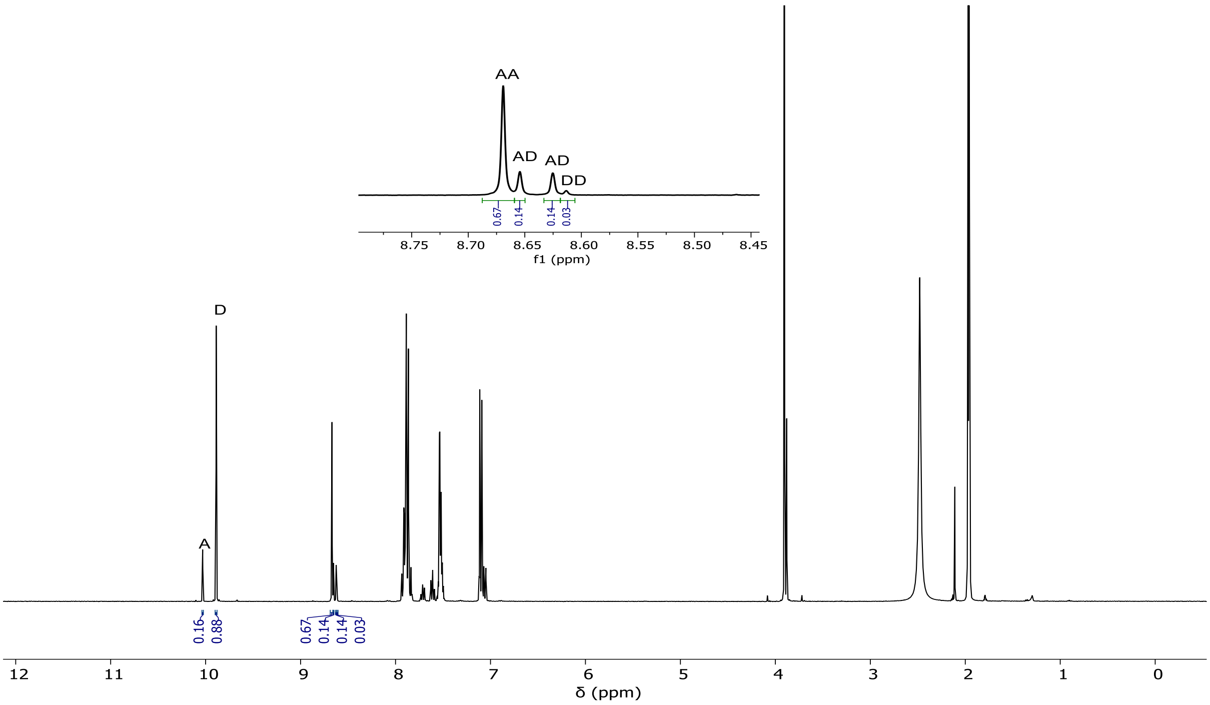

b)

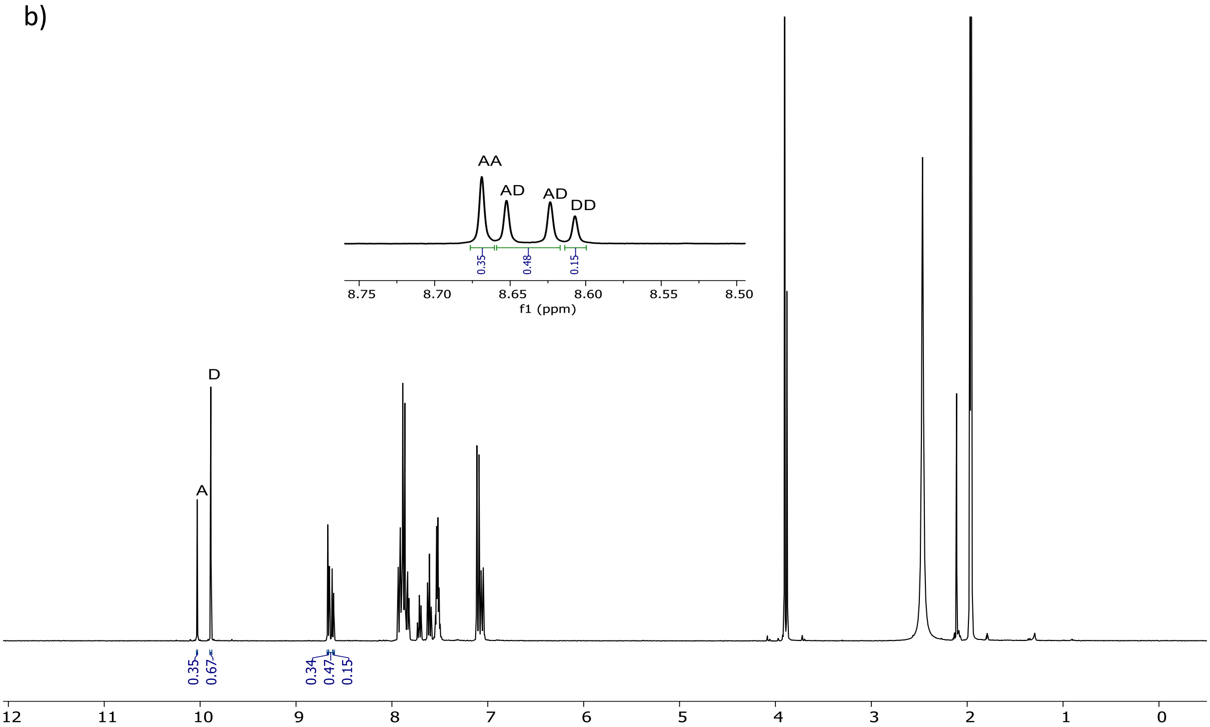

Figure S57: ^{1}H NMR (400 MHz, CD_3CN) spectra of **AA** + **D** equilibrium (7 mM **AA**, 14 mM **D**), in the presence of TFA (1 eq.), after a) 1 hour and b) 24 hours. No further change was observed upon longer equilibration times.

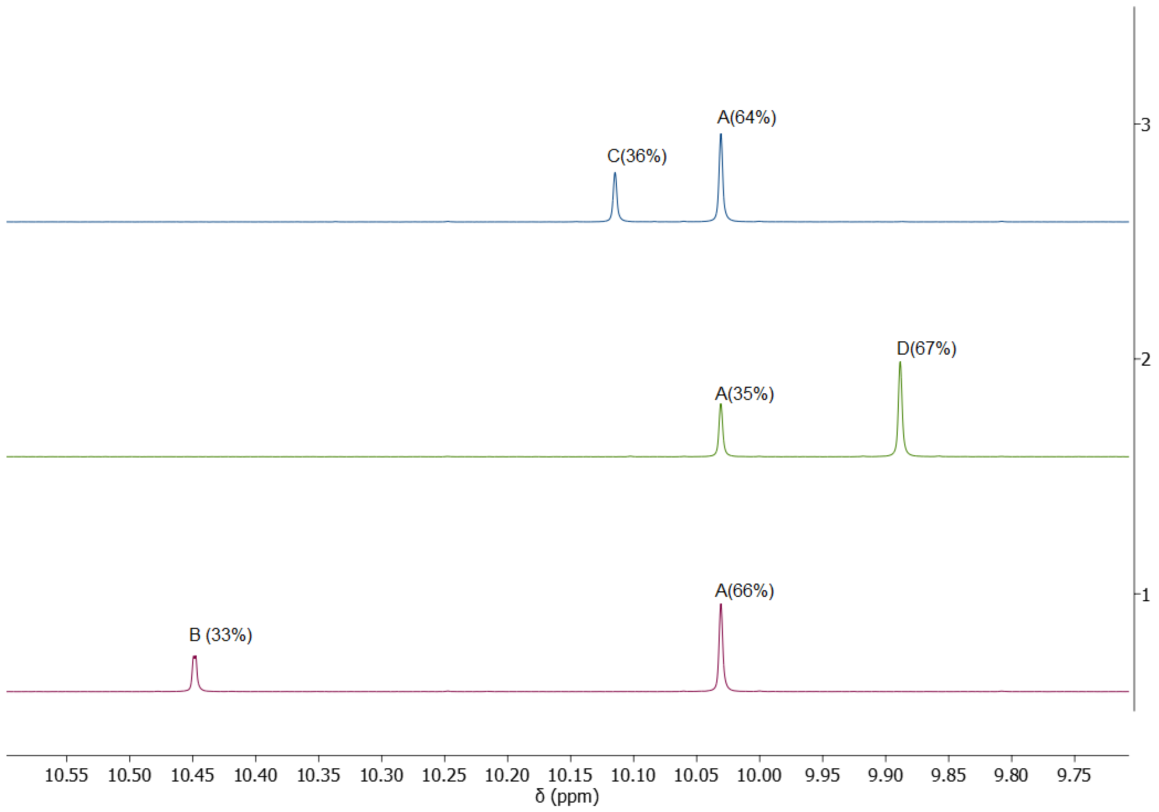

Figure S58: ^{1}H NMR (400 MHz, CD_3CN) spectra of the three above experiments after 24 hours, zoomed in at the aromatic region where the aldehyde carbonyl CH signals appear. The effect of substituents on Azine stability during exchange is exemplified by comparison of relative amounts of aldehydes present at equilibrium for the above three experiments. For electron withdrawing substituents (spectra 1 and 3) more benzaldehyde (**A**) is present in solution, indicating that azines formed from the aldehydes featuring electron withdrawing substituents (**B** and **C**) are more favoured. Accordingly, the electron donating substituent (spectrum 2) shows more anisaldehyde (**D**) than benzaldehyde (**A**) present at equilibrium, indicating that azines from benzaldehyde are more favoured in this case.

13. Azine metathesis reactions

In a typical experiment, the azines **AA** and **BB** or **CC** and **DD** (2 mM, 1 eq. of each one) were dissolved DMSO-d_6/H_2O and mixed in a 2 mL scintillation vial. The reaction proceeded by the addition of 1 equivalent of TFA, (V_{total}: 500 µL).

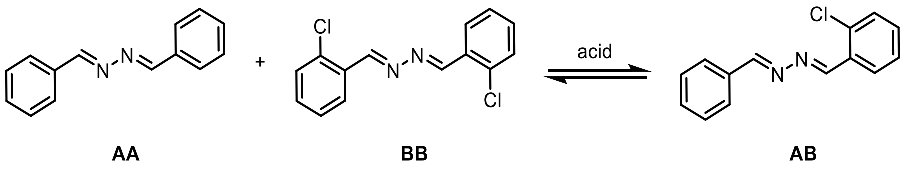

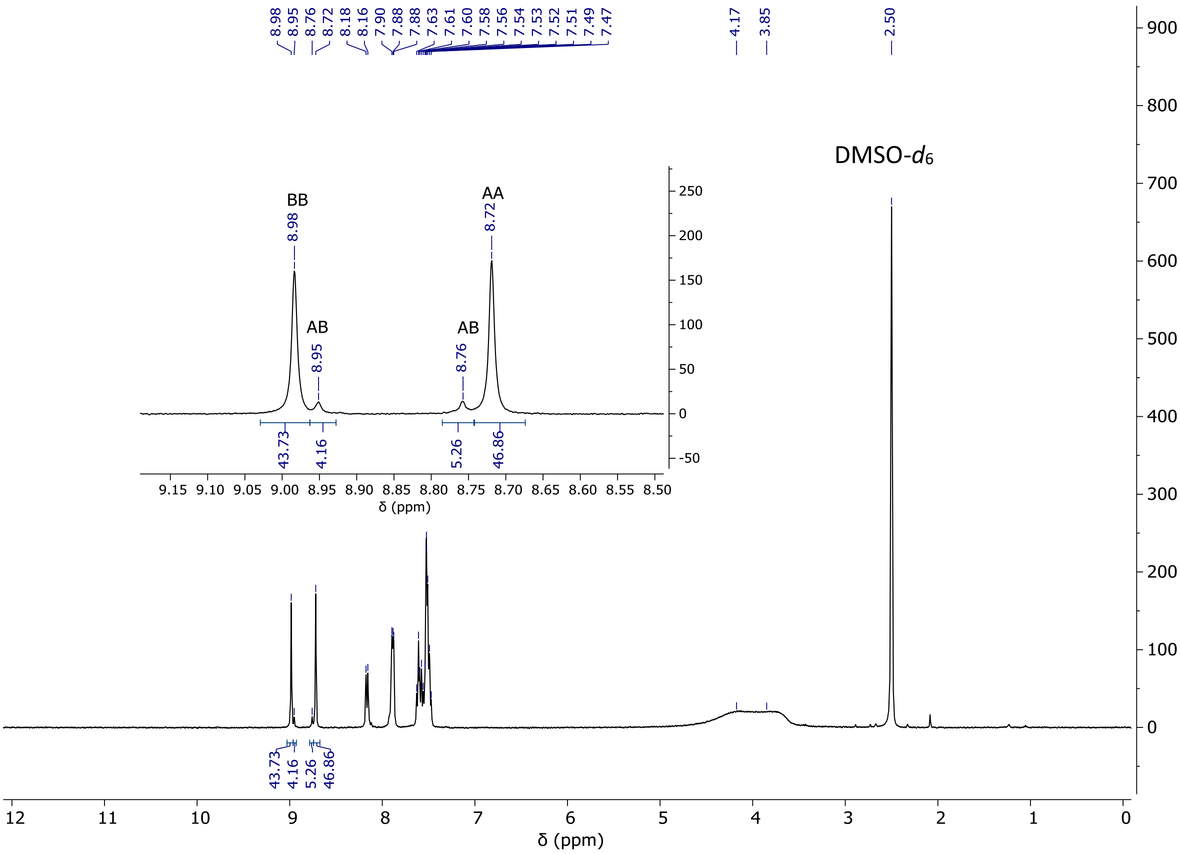

Figure S59: ^{1}H NMR spectrum of the **AA** and **BB** azine exchange reaction (2 mM in dry DMSO-d_6) in the presence of TFA (1 eq.), after 1 hour.

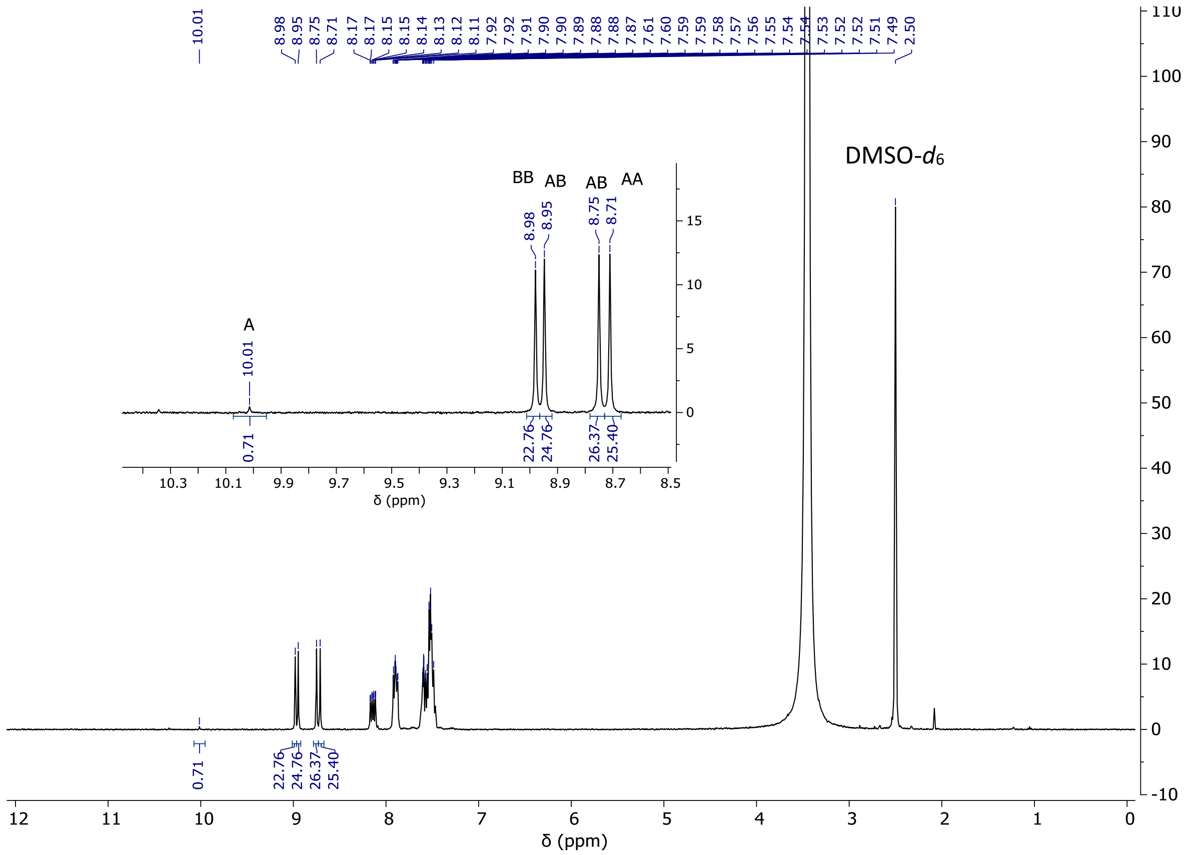

Figure S60: ^{1}H NMR spectrum of the **AA** and **BB** azine exchange reaction (2 mM in DMSO-d_6/H_2O, 95:5) in the presence of TFA (1 eq.), after 1 hour.

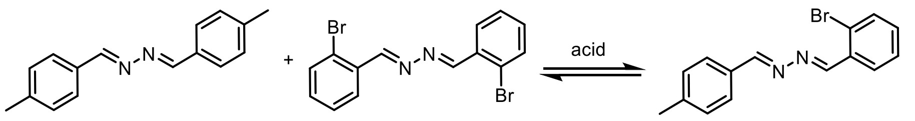

FF **GG** **FG**

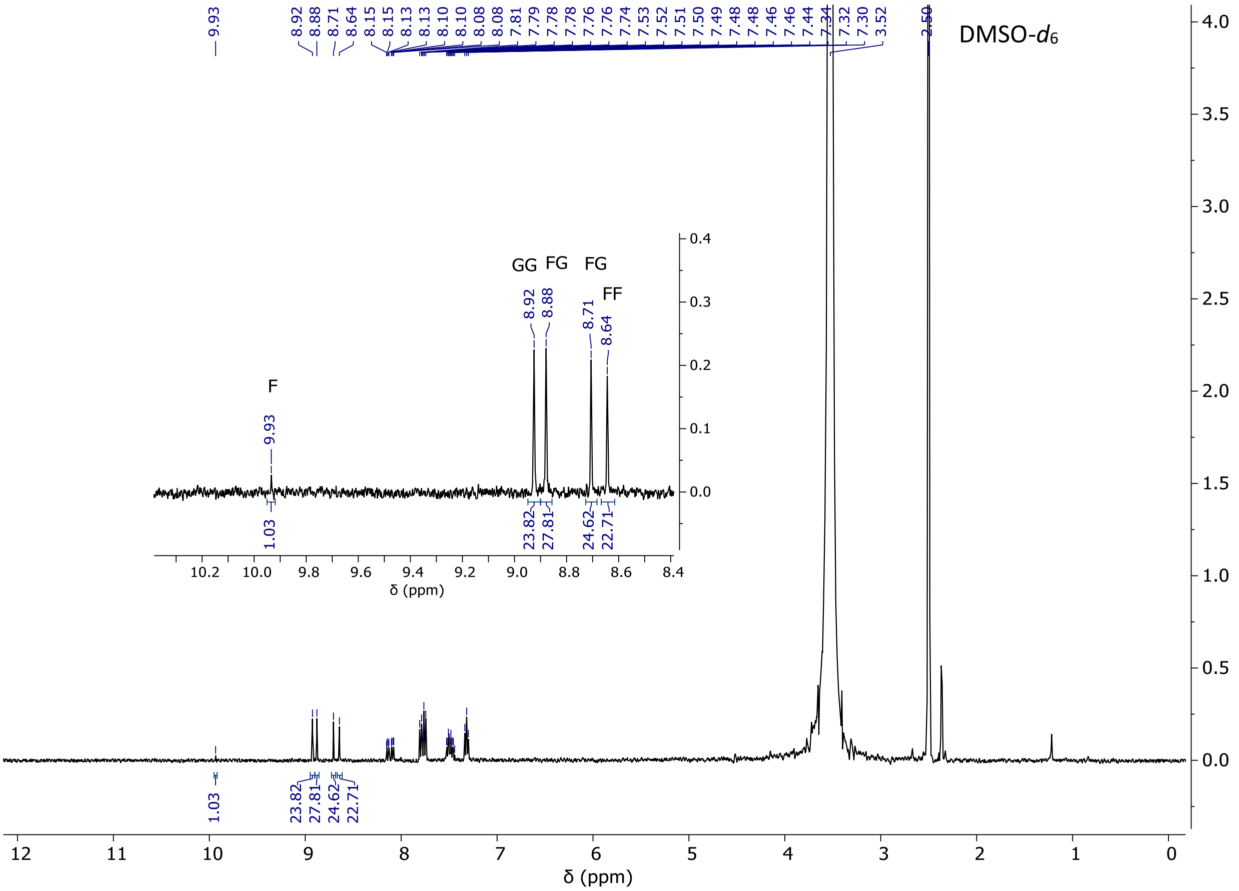

Figure S61: ^{1}H NMR spectrum of **FF** and **GG** azine exchange reaction (2 mM in DMSO-d_6/H_2O, 95:5) in the presence of TFA (1 eq.) after 1 hour.

14. Appendix 1

As a means of comparison to the stability studies carried out by Kalia and Raines[7] on oximes and hydrazones, azine **PP** was synthesized and its hydrolysis studied by a procedure adapted from the original one used for the oximes and hydrazones due to the solubility of azine **PP**.

Pivaldehyde azine (PP)

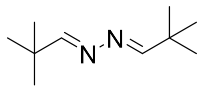

Pivaldehyde (1.26 mL, 11.61 mmol) was dissolved in 20 ml ethanol and hydrazine monohydrate (290 µL, 5.8 mmol, 0.5 eq.) was added. The resulting mixture was stirred for 2 hours, after which solvent was removed in vacuo until and oily solid was obtained. This solid was run through a plug of silica gel (3 cm in height) using pentane/Et_2O (95/5). The resulting solution was evaporated in vacuo and dried very briefly under high vacuum to yield the desired azine as a white crystalline solid (43 %, 423 mg, 3.69 mmol). Mp: 77.4-77.8. **^{1}H NMR** (300 MHz, $CDCl_3$) δ 7.66 (s, 2H), 1.13 (s, 18H). **^{13}C NMR** (101 MHz, $CDCl_3$) δ 170.85, 34.76, 27.27.

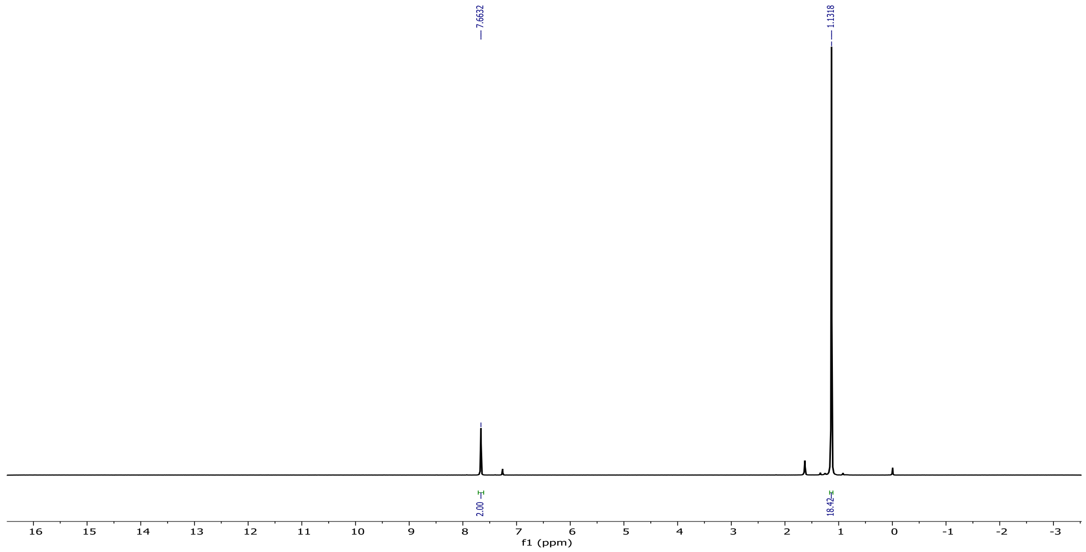

Figure S62: ^{1}H-NMR ($CDCl_3$, 300 MHz) of azine **PP**

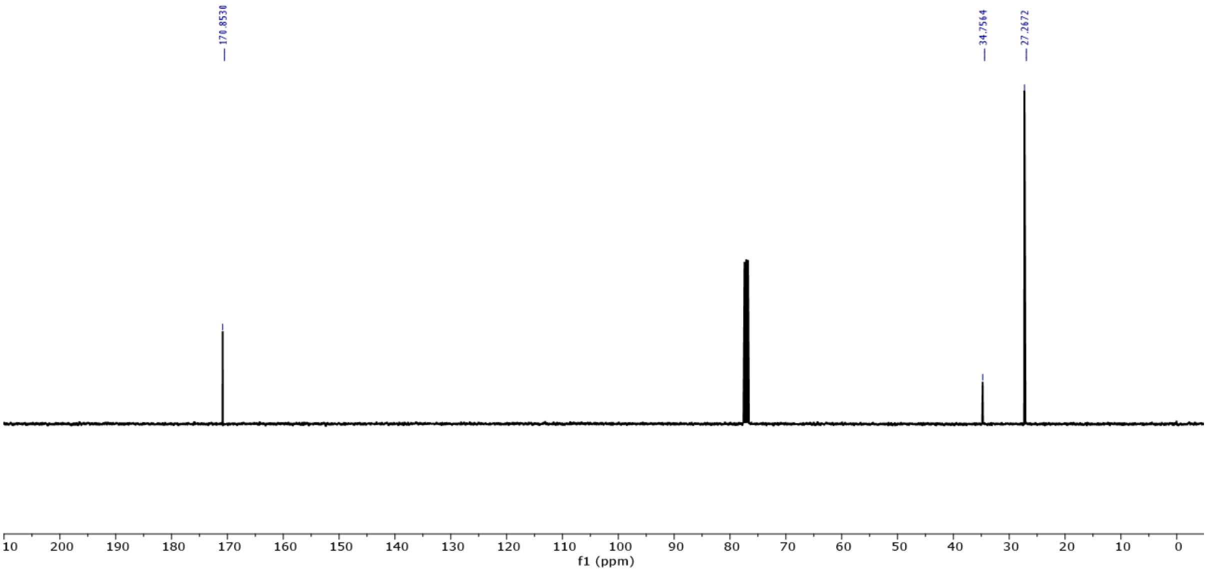

Figure S63: ^{13}C-NMR ($CDCl_3$, 101 MHz) of azine **PP**

Hydrolysis studies:

A 100 mM stock solution of **PP** in DMSO-d6 was added to a buffered D_2O solution containing formaldehyde (6 mM phosphate buffer, pD 5-9, 10 mM formaldehyde) to give a 1 mM solution of **PP**. The resulting solutions were monitored by ^{1}H NMR spectroscopy to yield kinetic traces for the hydrolysis of the azine from which first order rate constants and half-lifes could be obtained. The tBu signals at 1.10 to 0.72, originating from the azine, hydrazone, aldehyde and hydrated aldehyde were integrated and used as an internal reference. The integral of the azine azomethine CH signal was compared to the original amount of **PP** (based on the total tBu signal) to evaluate the degree of hydrolysis. The degree of hydrolysis versus time is plotted to give the kinetic trace of the hydrolysis reaction shown in Figure S64. These kinetic traces were then fitted to the equation below:

$$Y = Y_{max}(1 - e^{-kt})$$

Half-lives were calculated by the following equation:

$$\mathrm{t}_{1/2} = \frac{0.692}{\mathrm{k}}$$

Table S5 gives the first order rate constants as well as half-lifes at each pD. A sample of NMR spectra at selected times for the hydrolysis of azine **PP** at pD 5 can be seen in Figure S65

Table S5: First order rate constants and half-lives obtained from fits of the kinetic traces of the hydrolysis of azine **PP** at 1 mM in D_2O (1% DMSO, 6 mM phosphate buffer, 10 mM formaldehyde)

	**pD**	**k (min^{-1})**	**$\log_{10}$(k/min)**	**t$_{½}$ (min)**
**PP** hydrolysis	pD 5	0.02327	-1.63	30
	pD 6	0.01855	-1.73	37
	pD 7	0.01166	-1.92	58
	pD 8	0.00529	-2.27	131
	pD 9	0.00254	-2.59	269

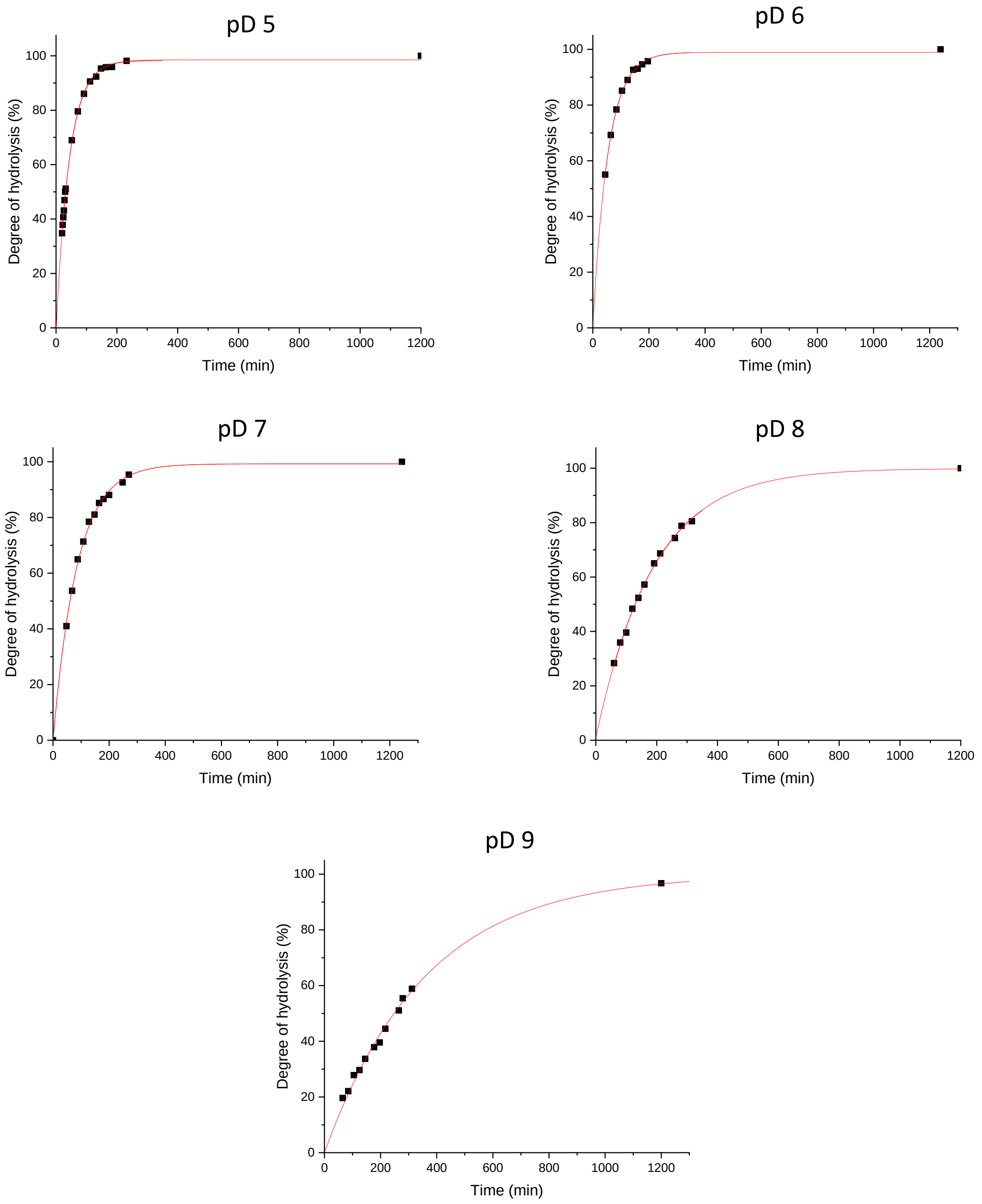

Figure S64: Kinetic traces of the hydrolysis of 1 mM **PP** in D_2O (1% DMSO, 6 mM phosphate buffer, 10 mM formaldehyde) at different pD's

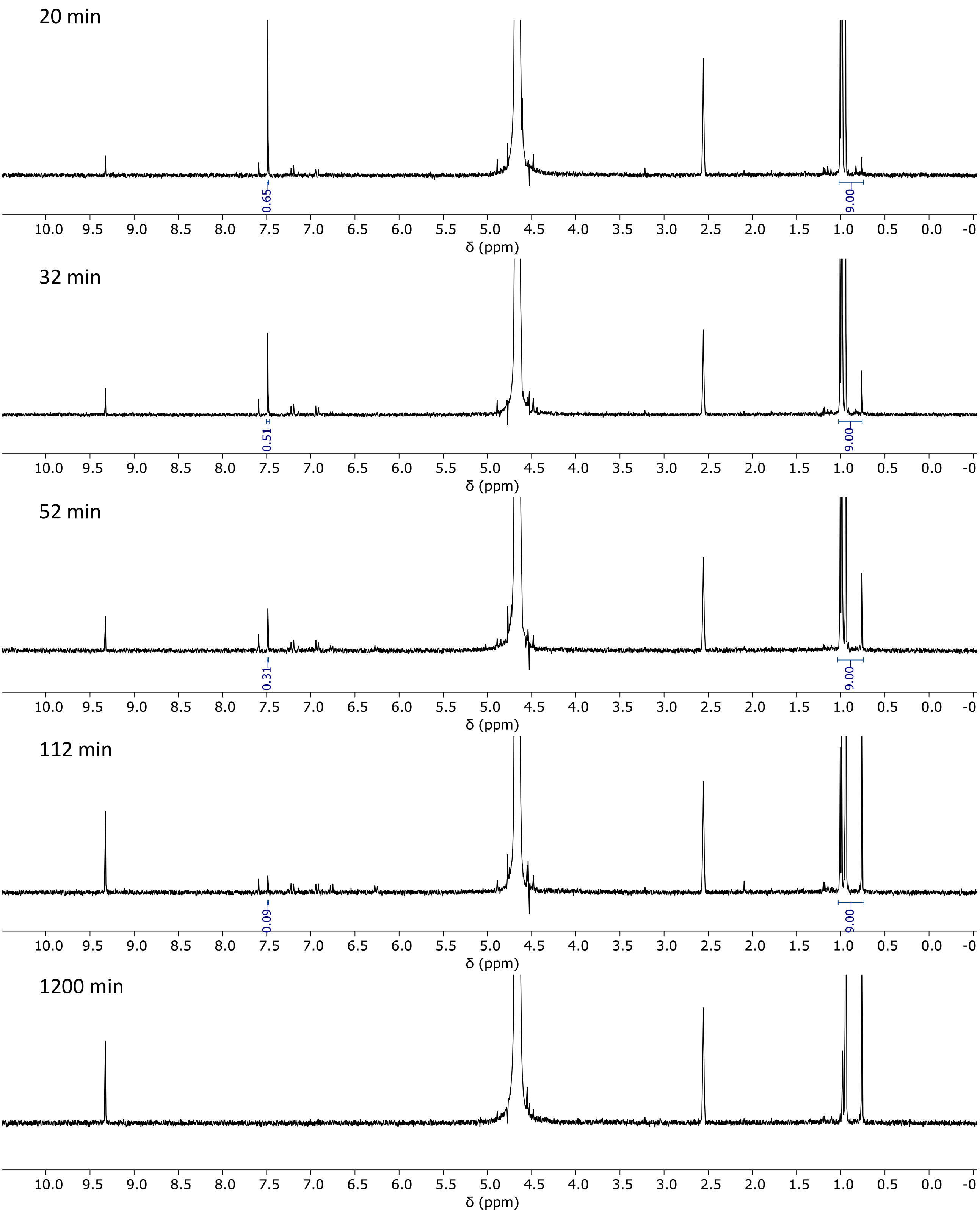

Figure S65: A selection of ^{1}H-NMR spectra (D_2O/DMSO-d6, 99:1, 6 mM pD 5 phosphate buffer, 10 mM formaldehyde) showing the hydrolysis of azine **PP** (1 mM) at different times.

15. References

[1] Bauer J. O., Leitus G., Ben-David Y., Milstein D., *ACS Catal.* 2016, *6*, 8415–8419.
[2] Shridevi D. D., Ningaiah S., Kuduva N.U, Yhya R. K., Lokanatha Rai K. M., *Synthetic Commun.* 2015, *45*, 2869-2875.
[3] Charistoudi, E.; Kallitsaki, M. G.; Charisteidis, I.; Kostas S. Triantafyllidis; Lykakisa, and I. N. *Adv.Synth. Catal.* 2017, No. 359, 2949 –2960.
[4] Dineshkumar, S.; Muthusamy, A. *Des. Monomers Polym.* 2017, *20* (1), 234–249
[5] Chakraborty M., Sengupta D., Saha T., Goswami S., *J. Org. Chem*. 2018, *83*, 7771–7778.
[6] McIlvaine, T. C. *J. Biol. Chem.* 1921, *49* (1), 183–186.
[7] Kalia, J. & Raines, R. T. *Angew. Chem. Int. Ed*. 2008, 47, 7523–7526.
